# Supplementary material for: Establishment and Characterization of OFT and OFO Cell Lines from Olive Flounder (Paralichthys olivaceus) for Use as Feeder Cells
Source: Biology (Basel). 2025 Feb 24;14(3):229. doi: 10.3390/biology14030229 (PMC11939788; doi:10.3390/biology14030229)
Supplement: Supplementary file 1 [file biology-14-00229-s001.zip › NCBI Blast_16s rRNA sequencing_OFT.pdf]

An official website of the United States government

Here's how you know

The .gov means it’s official.
Federal government websites often end in .gov or .mil. Before sharing sensitive information, make sure you’re on a federal government site.

Feedback

The site is secure.
The **https://** ensures that you are connecting to the official website and that any information you provide is encrypted and transmitted securely.
Access keys NCBI Homepage MyNCBI Homepage Main Content Main Navigation

BLAST ® » blastn suite » results for RID-09E1SU9T013

Job Title	FISHR2-01.ab1 ...
RID	09E1SU9T013 Search expires on 03-29 08:22 am
Results for	7:lcl|Query_20010 16SBR-01.ab1(542bp)
Program	BLASTN
Database	nt
Query ID	lcl|Query_20010
Description	16SBR-01.ab1 ...
Molecule type	dna
Query Length	542

Descriptions

Description	Scientific Name	Max Score	Total Score	Query Cover	E value	Per. Ident	Acc. Len	Accession
Paralichthys olivaceus x Verasper variegatus mitochondrion, complete genome	Paralichthys olivaceus x Verasper variegatus	1002	1002	100%	0.0	100.00%	16946	NC_082846.1
Teleost environmental sample mitochondrial gene for 16S ribosomal RNA, partial sequence, clone: OTU5	teleost environmental sample	1002	1002	100%	0.0	100.00%	594	LC074456.1
Paralichthys olivaceus mitochondrion, complete genome	Paralichthys olivaceus	996	996	100%	0.0	99.82%	17090	NC_002386.1
Paralichthys olivaceus 16S ribosomal RNA gene, partial sequence; mitochondrial gene for mitochondrial product	Paralichthys olivaceus	974	974	100%	0.0	99.08%	590	AY046953.1
Paralichthys adspersus mitochondrion, complete genome	Paralichthys adspersus	902	902	100%	0.0	96.68%	17060	NC_057273.1
Paralichthys adspersus 16S ribosomal RNA gene, partial sequence; mitochondrial	Paralichthys adspersus	902	902	100%	0.0	96.68%	637	HM211198.1
Paralichthys olivaceus PO1 mitochondrial gene for 16S rRNA, partial sequence	Paralichthys olivaceus	893	893	89%	0.0	99.79%	510	LC647901.1
Paralichthys californicus mitochondrion, complete genome	Paralichthys californicus	891	891	100%	0.0	96.32%	16858	MT859134.1
Paralichthys patagonicus voucher DAAPV F28 16S ribosomal RNA gene, partial sequence; mitochondrial	Paralichthys patagonicus	891	891	100%	0.0	96.32%	572	GU324157.1
Paralichthys californicus voucher KU 456 16S ribosomal RNA gene, partial sequence; mitochondrial	Paralichthys californicus	891	891	100%	0.0	96.32%	609	FJ870414.1
Paralichthys patagonicus 16S large subunit ribosomal RNA gene, partial sequence; mitochondrial	Paralichthys patagonicus	891	891	100%	0.0	96.32%	590	AY359657.1
Paralichthys californicus voucher UW:156295 16S ribosomal RNA gene, partial sequence; mitochondrial	Paralichthys californicus	889	889	99%	0.0	96.31%	590	MF134845.1
Paralichthys olivaceus PO3 mitochondrial gene for 16S rRNA, partial sequence	Paralichthys olivaceus	889	889	89%	0.0	99.79%	520	LC647903.1
Paralichthys albigutta voucher USNM:FISH:454751 mitochondrion, complete genome	Paralichthys albigutta	872	872	100%	0.0	95.76%	17035	NC_083031.1

Description	Scientific Name	Max Score	Total Score	Query Cover	E value	Per. Ident	Acc. Len	Accession
Paralichthys albigutta 16S large subunit ribosomal RNA gene, partial sequence; mitochondrial	Paralichthys albigutta	872	872	100%	0.0	95.76%	556	DQ532931.1
Paralichthys squamilentus voucher USNM:FISH:420936 mitochondrion, complete genome	Paralichthys squamilentus	867	867	100%	0.0	95.58%	16896	OP057002.2
Paralichthys lethostigma voucher KU 1 16S ribosomal RNA gene, partial sequence; mitochondrial	Paralichthys lethostigma	867	867	100%	0.0	95.58%	607	FJ870413.1
Paralichthys lethostigma clone 2 16S ribosomal RNA gene, partial sequence; mitochondrial	Paralichthys lethostigma	867	867	100%	0.0	95.58%	633	DQ450966.1
Paralichthys lethostigma clone 1 16S ribosomal RNA gene, partial sequence; mitochondrial	Paralichthys lethostigma	867	867	100%	0.0	95.58%	633	DQ450965.1
Paralichthys albigutta 16S ribosomal RNA gene, partial sequence; mitochondrial	Paralichthys albigutta	867	867	100%	0.0	95.57%	631	AY857937.2
Paralichthys californicus 16S ribosomal RNA gene, partial sequence; mitochondrial	Paralichthys californicus	857	857	96%	0.0	96.19%	561	AY952499.2
Paralichthys dentatus mitochondrion, complete genome	Paralichthys dentatus	856	856	100%	0.0	95.21%	17033	NC_029476.1
Paralichthys olivaceus mitochondrial 16S rRNA gene, isolate 197	Paralichthys olivaceus	837	837	83%	0.0	100.00%	1346	AM182432.1
Paralichthys dentatus voucher IOCASFY-Pdch01 16S ribosomal RNA gene, partial sequence; mitochondrial	Paralichthys dentatus	835	835	97%	0.0	95.10%	530	GU248346.1
Paralichthys lethostigma mitochondrion, complete genome	Paralichthys lethostigma	833	833	96%	0.0	95.43%	16843	NC_029223.1
Paralichthys dentatus 16S ribosomal RNA gene, partial sequence; mitochondrial gene for mitochondrial product	Paralichthys dentatus	832	832	99%	0.0	94.46%	549	AF488451.1
Ancylopsetta ommata voucher USNM:FISH:454750 mitochondrion, complete genome	Ancylopsetta ommata	824	824	100%	0.0	94.11%	16761	NC_083030.1
Paralichthys lethostigma voucher IOCASFY-Plch01 16S ribosomal RNA gene,	Paralichthys lethostigma	808	808	97%	0.0	94.17%	530	GU248348.1

Description	Scientific Name	Max Score	Total Score	Query Cover	E value	Per. Ident	Acc. Len	Accession
partial sequence; mitochondrial								
Hippoglossina oblonga voucher USNM:FISH:433277 mitochondrion, complete genome	Hippoglossina oblonga	802	802	100%	0.0	93.38%	17244	OP056997.2
Glyptocephalus zachirus mitochondrion, partial genome	Glyptocephalus zachirus	802	802	100%	0.0	93.37%	16549	OR499736.1
Psettichthys melanostictus voucher UW:47682 mitochondrion, complete genome	Psettichthys melanostictus	802	802	100%	0.0	93.37%	17149	NC_082806.1
Hippoglossus stenolepis voucher UW150612 16S ribosomal RNA gene, partial sequence; mitochondrial	Hippoglossus stenolepis	802	802	100%	0.0	93.39%	589	MT767343.1
Glyptocephalus zachirus voucher UW151770 16S ribosomal RNA gene, partial sequence; mitochondrial	Glyptocephalus zachirus	802	802	100%	0.0	93.37%	590	MT767340.1
Platichthys stellatus isolate 18-07 large subunit ribosomal RNA gene, partial sequence; mitochondrial	Platichthys stellatus	802	802	100%	0.0	93.38%	616	MN888903.1
Glyptocephalus zachirus voucher UW 047669 16S ribosomal RNA gene, partial sequence; mitochondrial	Glyptocephalus zachirus	802	802	100%	0.0	93.37%	611	FJ870388.1
Psettichthys melanostictus voucher WTU:047682 16S ribosomal RNA gene, partial sequence; mitochondrial	Psettichthys melanostictus	802	802	100%	0.0	93.37%	596	EF119311.1
Glyptocephalus zachirus voucher WTU:047669 16S ribosomal RNA gene, partial sequence; mitochondrial	Glyptocephalus zachirus	802	802	100%	0.0	93.37%	598	EF119300.1
Psettichthys melanostictus voucher WTU:047314 16S ribosomal RNA gene, partial sequence; mitochondrial	Psettichthys melanostictus	802	802	100%	0.0	93.37%	599	EF119260.1
Pleuronectes platessa isolate DM161a mitochondrion	Pleuronectes platessa	797	797	100%	0.0	93.19%	16805	MN122873.1
Hippoglossus stenolepis voucher UW157558 large subunit ribosomal RNA gene, partial sequence; mitochondrial	Hippoglossus stenolepis	797	797	100%	0.0	93.21%	585	MK804647.1
Platichthys environmental sample clone Pf4903 16S ribosomal RNA gene,	Platichthys environmental sample	797	797	100%	0.0	93.19%	588	KU510499.1

Description	Scientific Name	Max Score	Total Score	Query Cover	E value	Per. Ident	Acc. Len	Accession
partial sequence; mitochondrial								
Lepidopsetta mochigarei isolate PKU4824 16S ribosomal RNA gene, partial sequence; mitochondrial	Lepidopsetta mochigarei	797	797	100%	0.0	93.19%	603	KU936350.1
Glyptocephalus zachirus voucher UW:047670 mitochondrion, partial genome	Glyptocephalus zachirus	797	797	100%	0.0	93.19%	16688	OR575595.1
Lepidopsetta bilineata voucher UW:047661 mitochondrion, complete genome	Lepidopsetta bilineata	797	797	100%	0.0	93.19%	17054	OP035227.1
Lepidopsetta polyxystra voucher UW:48799 mitochondrion, complete genome	Lepidopsetta polyxystra	797	797	100%	0.0	93.19%	17055	NC_082812.1
Lepidopsetta bilineata voucher UW:110234 mitochondrion, complete genome	Lepidopsetta bilineata	797	797	100%	0.0	93.19%	17054	NC_082755.1
Pseudopleuronectes americanus voucher USNM:FISH:429777 mitochondrion, complete genome	Pseudopleuronectes americanus	797	797	100%	0.0	93.19%	17217	NC_082555.1
Platichthys stellatus x Verasper variegatus mitochondrion, complete genome	Platichthys stellatus x Verasper variegatus	797	797	100%	0.0	93.20%	16874	NC_082285.1
Hippoglossina oblonga isolate IFPGSR large subunit ribosomal RNA gene, partial sequence; mitochondrial	Hippoglossina oblonga	797	797	100%	0.0	93.20%	575	MW945438.1
Pleuronectes platessa isolate F30 large subunit ribosomal RNA gene, partial sequence; mitochondrial	Pleuronectes platessa	797	797	100%	0.0	93.19%	585	OM470926.1
Platichthys flesus voucher NRM:49642 16S ribosomal RNA gene, partial sequence; mitochondrial	Platichthys flesus	797	797	100%	0.0	93.19%	568	KJ128860.1
Lepidopsetta bilineata voucher UW151563 16S ribosomal RNA gene, partial sequence; mitochondrial	Lepidopsetta bilineata	797	797	100%	0.0	93.19%	590	MT767346.1
Lepidopsetta bilineata voucher UW153578 16S ribosomal RNA gene, partial sequence; mitochondrial	Lepidopsetta bilineata	797	797	100%	0.0	93.19%	590	MT767345.1
Glyptocephalus zachirus voucher UW155537 16S ribosomal RNA gene, partial sequence; mitochondrial	Glyptocephalus zachirus	797	797	100%	0.0	93.19%	590	MT767342.1
Glyptocephalus zachirus voucher UW151776 16S	Glyptocephalus zachirus	797	797	100%	0.0	93.19%	590	MT767341.1

Description	Scientific Name	Max Score	Total Score	Query Cover	E value	Per. Ident	Acc. Len	Accession
ribosomal RNA gene, partial sequence; mitochondrial								
Verasper moseri mitochondrion, complete genome	Verasper moseri	797	797	100%	0.0	93.21%	17443	LC583747.1
Hippoglossus stenolepis isolate HST12-2 large subunit ribosomal RNA gene, partial sequence; mitochondrial	Hippoglossus stenolepis	797	797	100%	0.0	93.21%	632	MN888916.1
Hippoglossus stenolepis isolate HST12-3 large subunit ribosomal RNA gene, partial sequence; mitochondrial	Hippoglossus stenolepis	797	797	100%	0.0	93.21%	616	MN888915.1
Platichthys stellatus isolate 16-07 large subunit ribosomal RNA gene, partial sequence; mitochondrial	Platichthys stellatus	797	797	100%	0.0	93.20%	623	MN888904.1
Platichthys stellatus isolate Ps2-011 large subunit ribosomal RNA gene, partial sequence; mitochondrial	Platichthys stellatus	797	797	100%	0.0	93.20%	630	MN888902.1
Platichthys stellatus isolate Ps3-011 large subunit ribosomal RNA gene, partial sequence; mitochondrial	Platichthys stellatus	797	797	100%	0.0	93.20%	610	MN888901.1
Platichthys stellatus isolate PS5-011 large subunit ribosomal RNA gene, partial sequence; mitochondrial	Platichthys stellatus	797	797	100%	0.0	93.20%	634	MN888899.1
Platichthys stellatus isolate PS6-011 large subunit ribosomal RNA gene, partial sequence; mitochondrial	Platichthys stellatus	797	797	100%	0.0	93.20%	629	MN888898.1
Lepidopsetta mochigarei isolate LMO12-2 large subunit ribosomal RNA gene, partial sequence; mitochondrial	Lepidopsetta mochigarei	797	797	100%	0.0	93.19%	632	MN888894.1
Pleuronectes platessa voucher DAAPV F51 16S ribosomal RNA gene, partial sequence; mitochondrial	Pleuronectes platessa	797	797	100%	0.0	93.19%	590	GU324168.1
Pleuronectes platessa voucher DAAPV F4 16S ribosomal RNA gene, partial sequence; mitochondrial	Pleuronectes platessa	797	797	100%	0.0	93.19%	590	GU324137.1
Reinhardtius hippoglossoides voucher UW 114782 16S ribosomal RNA gene, partial sequence; mitochondrial	Reinhardtius hippoglossoides	797	797	100%	0.0	93.21%	609	FJ870422.1

Description	Scientific Name	Max Score	Total Score	Query Cover	E value	Per. Ident	Acc. Len	Accession
Hippoglossus stenolepis voucher UW 048793 16S ribosomal RNA gene, partial sequence; mitochondrial	Hippoglossus stenolepis	797	797	100%	0.0	93.21%	609	FJ870421.1
Dexistes rikuzenius voucher FAKU 131267 16S ribosomal RNA gene, partial sequence; mitochondrial	Dexistes rikuzenius	797	797	100%	0.0	93.19%	607	FJ870420.1
Dexistes rikuzenius voucher FAKU 131266 16S ribosomal RNA gene, partial sequence; mitochondrial	Dexistes rikuzenius	797	797	100%	0.0	93.19%	602	FJ870419.1
Verasper moseri voucher UW 118096 16S ribosomal RNA gene, partial sequence; mitochondrial	Verasper moseri	797	797	100%	0.0	93.21%	611	FJ870415.1
Pseudopleuronectes americanus voucher KU 5419 16S ribosomal RNA gene, partial sequence; mitochondrial	Pseudopleuronectes americanus	797	797	100%	0.0	93.19%	611	FJ870412.1
Psettichthys melanostictus voucher UW 047314 16S ribosomal RNA gene, partial sequence; mitochondrial	Psettichthys melanostictus	797	797	100%	0.0	93.19%	608	FJ870404.1
Parophrys vetulus voucher UW 047297 16S ribosomal RNA gene, partial sequence; mitochondrial	Parophrys vetulus	797	797	100%	0.0	93.19%	609	FJ870399.1
Lepidopsetta bilineata voucher UW 048833 16S ribosomal RNA gene, partial sequence; mitochondrial	Lepidopsetta bilineata	797	797	100%	0.0	93.19%	611	FJ870392.1
Glyptocephalus zachirus voucher UW 047670 16S ribosomal RNA gene, partial sequence; mitochondrial	Glyptocephalus zachirus	797	797	100%	0.0	93.19%	611	FJ870389.1
Reinhardtius hippoglossoides complete mitochondrial genome, isolate Rh-4	Reinhardtius hippoglossoides	797	797	100%	0.0	93.21%	18078	AM749133.1
Reinhardtius hippoglossoides complete mitochondrial genome, isolate Rh-2	Reinhardtius hippoglossoides	797	797	100%	0.0	93.21%	18139	AM749131.1
Reinhardtius hippoglossoides mitochondrion, complete genome	Reinhardtius hippoglossoides	797	797	100%	0.0	93.21%	18017	NC_009711.1
Hippoglossus stenolepis complete mitochondrial genome, isolate Hs-4	Hippoglossus stenolepis	797	797	100%	0.0	93.21%	17902	AM749129.1
Hippoglossus stenolepis complete mitochondrial genome, isolate Hs-3	Hippoglossus stenolepis	797	797	100%	0.0	93.21%	17963	AM749128.1

Description	Scientific Name	Max Score	Total Score	Query Cover	E value	Per. Ident	Acc. Len	Accession
Hippoglossus stenolepis complete mitochondrial genome, isolate Hs-2	Hippoglossus stenolepis	797	797	100%	0.0	93.21%	17841	AM749127.1
Hippoglossus stenolepis mitochondrion, complete genome	Hippoglossus stenolepis	797	797	100%	0.0	93.21%	17841	NC_009710.1
Glyptocephalus zachirus voucher UW:049691 16S ribosomal RNA gene, partial sequence; mitochondrial	Glyptocephalus zachirus	797	797	100%	0.0	93.19%	598	EF458435.1
Hippoglossus stenolepis voucher UW:048793 16S ribosomal RNA gene, partial sequence; mitochondrial	Hippoglossus stenolepis	797	797	100%	0.0	93.21%	599	EF458356.1
Psettichthys melanostictus voucher WTU:047683 16S ribosomal RNA gene, partial sequence; mitochondrial	Psettichthys melanostictus	797	797	100%	0.0	93.19%	594	EF119312.1
Platichthys stellatus voucher WTU:047679 16S ribosomal RNA gene, partial sequence; mitochondrial	Platichthys stellatus	797	797	100%	0.0	93.19%	596	EF119308.1
Glyptocephalus zachirus voucher WTU:047670 16S ribosomal RNA gene, partial sequence; mitochondrial	Glyptocephalus zachirus	797	797	100%	0.0	93.19%	589	EF119301.1
Lepidopsetta bilineata voucher WTU:047662 16S ribosomal RNA gene, partial sequence; mitochondrial	Lepidopsetta bilineata	797	797	100%	0.0	93.19%	598	EF119293.1
Lepidopsetta bilineata voucher WTU:047661 16S ribosomal RNA gene, partial sequence; mitochondrial	Lepidopsetta bilineata	797	797	100%	0.0	93.19%	599	EF119292.1
Psettichthys melanostictus voucher WTU:047293 16S ribosomal RNA gene, partial sequence; mitochondrial	Psettichthys melanostictus	797	797	100%	0.0	93.19%	595	EF119241.1
Verasper moseri mitochondrion, complete genome	Verasper moseri	797	797	100%	0.0	93.21%	17588	NC_008461.1
Verasper moseri 16S ribosomal RNA gene, complete sequence; mitochondrial	Verasper moseri	797	797	100%	0.0	93.21%	1716	DQ834443.2
Verasper moseri 16S ribosomal RNA gene, partial sequence; mitochondrial	Verasper moseri	797	797	100%	0.0	93.21%	591	DQ242489.1
Platichthys flesus 16S large subunit ribosomal RNA gene, partial sequence; mitochondrial	Platichthys flesus	797	797	100%	0.0	93.19%	582	AY359670.1
Paralichthys oblongus 16S ribosomal RNA gene,	Hippoglossina oblonga	797	797	100%	0.0	93.20%	592	AF420448.1

Description	Scientific Name	Max Score	Total Score	Query Cover	E value	Per. Ident	Acc. Len	Accession
partial sequence; mitochondrial gene for mitochondrial product								
Glyptocephalus cynoglossus 16S ribosomal RNA gene, partial sequence; mitochondrial gene for mitochondrial product	Glyptocephalus cynoglossus	797	797	100%	0.0	93.20%	593	AF420447.1
Pleuronectes platessa 16S large subunit ribosomal RNA gene, partial sequence; mitochondrial gene for mitochondrial product	Pleuronectes platessa	797	797	100%	0.0	93.19%	569	AY157328.1
Kareius bicoloratus mitochondrion, complete genome	Kareius bicoloratus	797	797	100%	0.0	93.19%	15973	NC_003176.1
Lepidopsetta bilineata 16S ribosomal RNA gene, partial sequence; mitochondrial gene for mitochondrial product	Lepidopsetta bilineata	795	795	99%	0.0	93.16%	552	AF488429.1

Graphic Summary

Distribution of the top 100 Blast Hits on 100 subject sequences

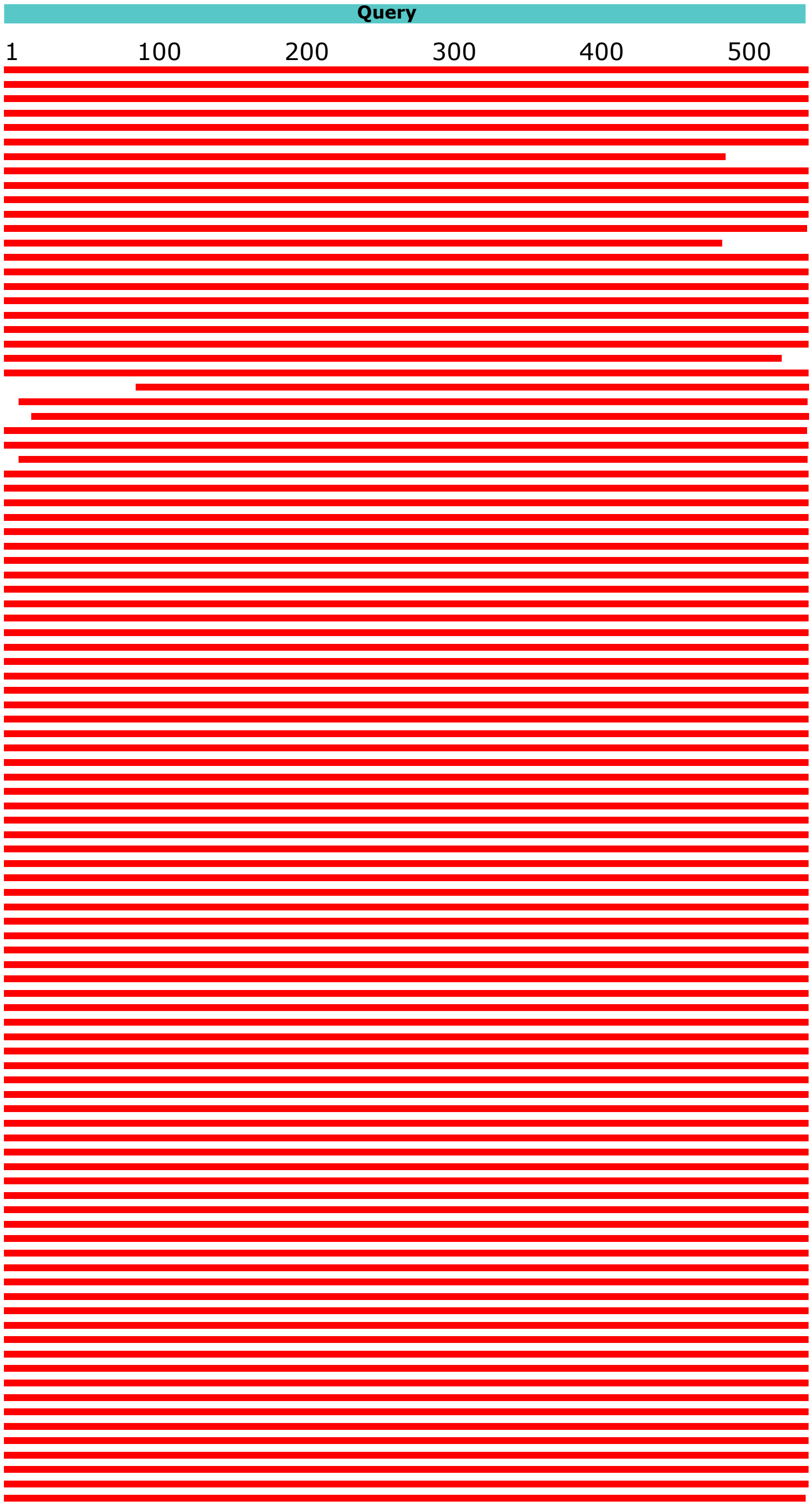

Alignments

Alignment view Pairwise ☐ CDS feature Restore defaults

Paralichthys olivaceus x Verasper variegatus mitochondrion, complete genome

Sequence ID: **NC_082846.1** Length: 16946 Number of Matches: 1

Range 1: 2062 to 2603

Score	Expect	Identities	Gaps	Strand	Frame
1002 bits(542)	0.0()	542/542(100%)	0/542(0%)	Plus/Minus	

```
Query  1     CGAACCCTTAATAGCGGCTGCACCATTAGGATGTCCTGATCCAACATCGAGGTCGTAAAC  60
             ||||||||||||||||||||||||||||||||||||||||||||||||||||||||||||
Sbjct  2603  CGAACCCTTAATAGCGGCTGCACCATTAGGATGTCCTGATCCAACATCGAGGTCGTAAAC  2544

Query  61    CCCCTTGTCGATATGGGCTCTAAAAGGGGATTGCGCTGTTATCCCTAGGGTAACTTGGTT  120
             ||||||||||||||||||||||||||||||||||||||||||||||||||||||||||||
Sbjct  2543  CCCCTTGTCGATATGGGCTCTAAAAGGGGATTGCGCTGTTATCCCTAGGGTAACTTGGTT  2484
```

```
Query  121   CGTTGATCGGCGTTGCCGGATCAGTTTGGTCAGAATTTCTGCTGATTAGAGCTGTCGCTC  180
             ||||||||||||||||||||||||||||||||||||||||||||||||||||||||||||
Sbjct  2483  CGTTGATCGGCGTTGCCGGATCAGTTTGGTCAGAATTTCTGCTGATTAGAGCTGTCGCTC  2424

Query  181   TAGCTTGTAGGAGGAGAGGAATGTAGGGGTGTACTCCTTTTCCACGTGGGGGTTTTGTGT  240
             ||||||||||||||||||||||||||||||||||||||||||||||||||||||||||||
Sbjct  2423  TAGCTTGTAGGAGGAGAGGAATGTAGGGGTGTACTCCTTTTCCACGTGGGGGTTTTGTGT  2364

Query  241   TCCCCATGGTCGCCCCAACCGAAGACATCAGGGCTGGTTTCATTTAGTTCAGGGCCCTTA  300
             ||||||||||||||||||||||||||||||||||||||||||||||||||||||||||||
Sbjct  2363  TCCCCATGGTCGCCCCAACCGAAGACATCAGGGCTGGTTTCATTTAGTTCAGGGCCCTTA  2304

Query  301   GCTGGGTGTATTTGACATGATCTGCCCTTGCGTCTAAAGCTCCATAGGGTCTTCTCGTCT  360
             ||||||||||||||||||||||||||||||||||||||||||||||||||||||||||||
Sbjct  2303  GCTGGGTGTATTTGACATGATCTGCCCTTGCGTCTAAAGCTCCATAGGGTCTTCTCGTCT  2244

Query  361   TATGAGCTTATCCCCGCTTCTGCACGGGGAGATCAATTTCATTGACTGGGGGAAGGAGAC  420
             ||||||||||||||||||||||||||||||||||||||||||||||||||||||||||||
Sbjct  2243  TATGAGCTTATCCCCGCTTCTGCACGGGGAGATCAATTTCATTGACTGGGGGAAGGAGAC  2184

Query  421   AGCTAAGCCCTCGTTATGCCATTCATACGGGTCTTCATTTAAAAGACAAGTGATTACGCT  480
             ||||||||||||||||||||||||||||||||||||||||||||||||||||||||||||
Sbjct  2183  AGCTAAGCCCTCGTTATGCCATTCATACGGGTCTTCATTTAAAAGACAAGTGATTACGCT  2124

Query  481   ACCTTTGCACGGTCAAAATACCGCGGCCGTTGAACTATGATGTCACTGGGCAGGCGGGAC  540
             ||||||||||||||||||||||||||||||||||||||||||||||||||||||||||||
Sbjct  2123  ACCTTTGCACGGTCAAAATACCGCGGCCGTTGAACTATGATGTCACTGGGCAGGCGGGAC  2064

Query  541   CT  542
             ||
Sbjct  2063  CT  2062
```

Teleost environmental sample mitochondrial gene for 16S ribosomal RNA, partial sequence, clone: OTU5

Sequence ID: **LC074456.1** Length: 594 Number of Matches: 1

Range 1: 28 to 569

Score	Expect	Identities	Gaps	Strand	Frame
1002 bits(542)	0.0()	542/542(100%)	0/542(0%)	Plus/Minus	

```
Query  1    CGAACCCTTAATAGCGGCTGCACCATTAGGATGTCCTGATCCAACATCGAGGTCGTAAAC  60
            ||||||||||||||||||||||||||||||||||||||||||||||||||||||||||||
Sbjct  569  CGAACCCTTAATAGCGGCTGCACCATTAGGATGTCCTGATCCAACATCGAGGTCGTAAAC  510

Query  61   CCCCTTGTCGATATGGGCTCTAAAAGGGGATTGCGCTGTTATCCCTAGGGTAACTTGGTT  120
            ||||||||||||||||||||||||||||||||||||||||||||||||||||||||||||
Sbjct  509  CCCCTTGTCGATATGGGCTCTAAAAGGGGATTGCGCTGTTATCCCTAGGGTAACTTGGTT  450

Query  121  CGTTGATCGGCGTTGCCGGATCAGTTTGGTCAGAATTTCTGCTGATTAGAGCTGTCGCTC  180
            ||||||||||||||||||||||||||||||||||||||||||||||||||||||||||||
Sbjct  449  CGTTGATCGGCGTTGCCGGATCAGTTTGGTCAGAATTTCTGCTGATTAGAGCTGTCGCTC  390

Query  181  TAGCTTGTAGGAGGAGAGGAATGTAGGGGTGTACTCCTTTTCCACGTGGGGGTTTTGTGT  240
            ||||||||||||||||||||||||||||||||||||||||||||||||||||||||||||
Sbjct  389  TAGCTTGTAGGAGGAGAGGAATGTAGGGGTGTACTCCTTTTCCACGTGGGGGTTTTGTGT  330

Query  241  TCCCCATGGTCGCCCCAACCGAAGACATCAGGGCTGGTTTCATTTAGTTCAGGGCCCTTA  300
            ||||||||||||||||||||||||||||||||||||||||||||||||||||||||||||
Sbjct  329  TCCCCATGGTCGCCCCAACCGAAGACATCAGGGCTGGTTTCATTTAGTTCAGGGCCCTTA  270

Query  301  GCTGGGTGTATTTGACATGATCTGCCCTTGCGTCTAAAGCTCCATAGGGTCTTCTCGTCT  360
            ||||||||||||||||||||||||||||||||||||||||||||||||||||||||||||
Sbjct  269  GCTGGGTGTATTTGACATGATCTGCCCTTGCGTCTAAAGCTCCATAGGGTCTTCTCGTCT  210

Query  361  TATGAGCTTATCCCCGCTTCTGCACGGGGAGATCAATTTCATTGACTGGGGGAAGGAGAC  420
            ||||||||||||||||||||||||||||||||||||||||||||||||||||||||||||
Sbjct  209  TATGAGCTTATCCCCGCTTCTGCACGGGGAGATCAATTTCATTGACTGGGGGAAGGAGAC  150

Query  421  AGCTAAGCCCTCGTTATGCCATTCATACGGGTCTTCATTTAAAAGACAAGTGATTACGCT  480
            ||||||||||||||||||||||||||||||||||||||||||||||||||||||||||||
Sbjct  149  AGCTAAGCCCTCGTTATGCCATTCATACGGGTCTTCATTTAAAAGACAAGTGATTACGCT  90

Query  481  ACCTTTGCACGGTCAAAATACCGCGGCCGTTGAACTATGATGTCACTGGGCAGGCGGGAC  540
            ||||||||||||||||||||||||||||||||||||||||||||||||||||||||||||
Sbjct  89   ACCTTTGCACGGTCAAAATACCGCGGCCGTTGAACTATGATGTCACTGGGCAGGCGGGAC  30

Query  541  CT  542
            ||
Sbjct  29   CT  28
```

Paralichthys olivaceus mitochondrion, complete genome

Sequence ID: **NC_002386.1** Length: 17090 Number of Matches: 1

Range 1: 2062 to 2603

Score	Expect	Identities	Gaps	Strand	Frame
996 bits(539)	0.0()	541/542(99%)	0/542(0%)	Plus/Minus	

```
Query  1     CGAACCCTTAATAGCGGCTGCACCATTAGGATGTCCTGATCCAACATCGAGGTCGTAAAC  60
             ||||||||||||||||||||||||||||||||||||||||||||||||||||||||||||
Sbjct  2603  CGAACCCTTAATAGCGGCTGCACCATTAGGATGTCCTGATCCAACATCGAGGTCGTAAAC  2544

Query  61    CCCCTTGTCGATATGGGCTCTAAAAGGGGATTGCGCTGTTATCCCTAGGGTAACTTGGTT  120
             ||||||||||||||||||||||||||||||||||||||||||||||||||||||||||||
```

```
Sbjct  2543  CCCCTTGTCGATATGGGCTCTAAAAGGGGATTGCGCTGTTATCCCTAGGGTAACTTGGTT  2484

Query  121   CGTTGATCGGCGTTGCCGGATCAGTTTGGTCAGAATTTCTGCTGATTAGAGCTGTCGCTC  180
             ||||||||||||||||||||||||||||||||||||||||||||||||||||||||||||
Sbjct  2483  CGTTGATCGGCGTTGCCGGATCAGTTTGGTCAGAATTTCTGCTGATTAGAGCTGTCGCTC  2424

Query  181   TAGCTTGTAGGAGGAGAGGAATGTAGGGGTGTACTCCTTTTCCACGTGGGGGTTTTGTGT  240
             ||||||||||||||||||||||||||||||||||||||||||||||||||||||||||||
Sbjct  2423  TAGCTTGTAGGAGGAGAGGAATGTAGGGGTGTACTCCTTTTCCACGTGGGGGTTTTGTGT  2364

Query  241   TCCCCATGGTCGCCCCAACCGAAGACATCAGGGCTGGTTTCATTTAGTTCAGGGCCCTTA  300
             ||||||||||||||||||||||||||||||||||||||||||||||||||||||||||||
Sbjct  2363  TCCCCATGGTCGCCCCAACCGAAGACATCAGGGCTGGTTTCATTTAGTTCAGGGCCCTTA  2304

Query  301   GCTGGGTGTATTTGACATGATCTGCCCTTGCGTCTAAAGCTCCATAGGGTCTTCTCGTCT  360
             ||||||||||||||||||||||||||||||||||||||||||||||||||||||||||||
Sbjct  2303  GCTGGGTGTATTTGACATGATCTGCCCTTGCGTCTAAAGCTCCATAGGGTCTTCTCGTCT  2244

Query  361   TATGAGCTTATCCCCGCTTCTGCACGGGGAGATCAATTTCATTGACTGGGGGAAGGAGAC  420
             |||||| |||||||||||||||||||||||||||||||||||||||||||||||||||||
Sbjct  2243  TATGAGGTTATCCCCGCTTCTGCACGGGGAGATCAATTTCATTGACTGGGGGAAGGAGAC  2184

Query  421   AGCTAAGCCCTCGTTATGCCATTCATACGGGTCTTCATTTAAAAGACAAGTGATTACGCT  480
             ||||||||||||||||||||||||||||||||||||||||||||||||||||||||||||
Sbjct  2183  AGCTAAGCCCTCGTTATGCCATTCATACGGGTCTTCATTTAAAAGACAAGTGATTACGCT  2124

Query  481   ACCTTTGCACGGTCAAAATACCGCGGCCGTTGAACTATGATGTCACTGGGCAGGCGGGAC  540
             ||||||||||||||||||||||||||||||||||||||||||||||||||||||||||||
Sbjct  2123  ACCTTTGCACGGTCAAAATACCGCGGCCGTTGAACTATGATGTCACTGGGCAGGCGGGAC  2064

Query  541   CT  542
             ||
Sbjct  2063  CT  2062
```

Paralichthys olivaceus 16S ribosomal RNA gene, partial sequence; mitochondrial gene for mitochondrial product

Sequence ID: **AY046953.1** Length: 590 Number of Matches: 1

Range 1: 23 to 564

Score	Expect	Identities	Gaps	Strand	Frame
974 bits(527)	0.0()	537/542(99%)	0/542(0%)	Plus/Plus	

```
Query  1    CGAACCCTTAATAGCGGCTGCACCATTAGGATGTCCTGATCCAACATCGAGGTCGTAAAC  60
            ||||||||||||||||||||||||||||||||||| ||||||||||||||||||||||||
Sbjct  23   CGAACCCTTAATAGCGGCTGCACCATTAGGATGTCGTGATCCAACATCGAGGTCGTAAAC  82

Query  61   CCCCTTGTCGATATGGGCTCTAAAAGGGGATTGCGCTGTTATCCCTAGGGTAACTTGGTT  120
            ||||||||||||||||||||||||||||||||||||||||||||||||||||||||||||
Sbjct  83   CCCCTTGTCGATATGGGCTCTAAAAGGGGATTGCGCTGTTATCCCTAGGGTAACTTGGTT  142

Query  121  CGTTGATCGGCGTTGCCGGATCAGTTTGGTCAGAATTTCTGCTGATTAGAGCTGTCGCTC  180
            ||||||| |||||||||||||||||||||||||||||||||||||||||||||||||| |
Sbjct  143  CGTTGATTGGCGTTGCCGGATCAGTTTGGTCAGAATTTCTGCTGATTAGAGCTGTCGCGC  202

Query  181  TAGCTTGTAGGAGGAGAGGAATGTAGGGGTGTACTCCTTTTCCACGTGGGGGTTTTGTGT  240
            ||| ||||||||||||||||||||||||||||||||||||||||||||||||||||||||
Sbjct  203  TAGTTTGTAGGAGGAGAGGAATGTAGGGGTGTACTCCTTTTCCACGTGGGGGTTTTGTGT  262

Query  241  TCCCCATGGTCGCCCCAACCGAAGACATCAGGGCTGGTTTCATTTAGTTCAGGGCCCTTA  300
            ||||||||||||||||||||||||||||||||||||||||||||||||||||||||||||
Sbjct  263  TCCCCATGGTCGCCCCAACCGAAGACATCAGGGCTGGTTTCATTTAGTTCAGGGCCCTTA  322

Query  301  GCTGGGTGTATTTGACATGATCTGCCCTTGCGTCTAAAGCTCCATAGGGTCTTCTCGTCT  360
            ||||||||||||||||||||||||||||||||||||||||||||||||||||||||||||
Sbjct  323  GCTGGGTGTATTTGACATGATCTGCCCTTGCGTCTAAAGCTCCATAGGGTCTTCTCGTCT  382

Query  361  TATGAGCTTATCCCCGCTTCTGCACGGGGAGATCAATTTCATTGACTGGGGGAAGGAGAC  420
            |||||| |||||||||||||||||||||||||||||||||||||||||||||||||||||
Sbjct  383  TATGAGGTTATCCCCGCTTCTGCACGGGGAGATCAATTTCATTGACTGGGGGAAGGAGAC  442

Query  421  AGCTAAGCCCTCGTTATGCCATTCATACGGGTCTTCATTTAAAAGACAAGTGATTACGCT  480
            ||||||||||||||||||||||||||||||||||||||||||||||||||||||||||||
Sbjct  443  AGCTAAGCCCTCGTTATGCCATTCATACGGGTCTTCATTTAAAAGACAAGTGATTACGCT  502

Query  481  ACCTTTGCACGGTCAAAATACCGCGGCCGTTGAACTATGATGTCACTGGGCAGGCGGGAC  540
            ||||||||||||||||||||||||||||||||||||||||||||||||||||||||||||
Sbjct  503  ACCTTTGCACGGTCAAAATACCGCGGCCGTTGAACTATGATGTCACTGGGCAGGCGGGAC  562

Query  541  CT  542
            ||
Sbjct  563  CT  564
```

Paralichthys adspersus mitochondrion, complete genome

Sequence ID: **NC_057273.1** Length: 17060 Number of Matches: 1

Range 1: 2063 to 2604

Score	Expect	Identities	Gaps	Strand	Frame
902 bits(488)	0.0()	524/542(97%)	0/542(0%)	Plus/Minus	

```
Query  1     CGAACCCTTAATAGCGGCTGCACCATTAGGATGTCCTGATCCAACATCGAGGTCGTAAAC  60
             ||||||||||||||||||||||||||||||||||||||||||||||||||||||||||||
Sbjct  2604  CGAACCCTTAATAGCGGCTGCACCATTAGGATGTCCTGATCCAACATCGAGGTCGTAAAC  2545

Query  61    CCCCTTGTCGATATGGGCTCTAAAAGGGGATTGCGCTGTTATCCCTAGGGTAACTTGGTT  120
```

```
             ||||||||||||||||||||||||||||||||||||||||||||||||||||||||| ||
Sbjct  2544  CCCCTTGTCGATATGGGCTCTAAAAGGGGATTGCGCTGTTATCCCTAGGGTAACTTGATT  2485

Query  121   CGTTGATCGGCGTTGCCGGATCAGTTTGGTCAGAATTTCTGCTGATTAGAGCTGTCGCTC  180
             ||||||||||| ||||||||||||| ||||||||||||||||||||||||||||||||||
Sbjct  2484  CGTTGATCGGCTTTGCCGGATCAGTCTGGTCAGAATTTCTGCTGATTAGAGCTGTCGCTC  2425

Query  181   TAGCTTGTAGGAGGAGAGGAATGTAGGGGTGTACTCCTTTTCCACGTGGGGGTTTTGTGT  240
             ||| |||||||||||| | |||||||||||||||||||||||||||||||||||||||||
Sbjct  2424  TAGTTTGTAGGAGGAGGGAAATGTAGGGGTGTACTCCTTTTCCACGTGGGGGTTTTGTGT  2365

Query  241   TCCCCATGGTCGCCCCAACCGAAGACATCAGGGCTGGTTTCATTTAGTTCAGGGCCCTTA  300
             |||||||||||||||||||||||||||||| |||||| ||||||||||||||| ||| ||
Sbjct  2364  TCCCCATGGTCGCCCCAACCGAAGACATCAAGGCTGGCTTCATTTAGTTCAGGTCCCATA  2305

Query  301   GCTGGGTGTATTTGACATGATCTGCCCTTGCGTCTAAAGCTCCATAGGGTCTTCTCGTCT  360
             |||||||||||||||||||||||||| |||||||||||||||||||||||||||||||||
Sbjct  2304  GCTGGGTGTATTTGACATGATCTGCCTTTGCGTCTAAAGCTCCATAGGGTCTTCTCGTCT  2245

Query  361   TATGAGCTTATCCCCGCTTCTGCACGGGGAGATCAATTTCATTGACTGGGGGAAGGAGAC  420
             ||||||| ||||||||||||||||||||||||||||||||||||||  ||||||||||||
Sbjct  2244  TATGAGCATATCCCCGCTTCTGCACGGGGAGATCAATTTCATTGACCAGGGGAAGGAGAC  2185

Query  421   AGCTAAGCCCTCGTTATGCCATTCATACGGGTCTTCATTTAAAAGACAAGTGATTACGCT  480
             || |||||||||||||||||||||||||||||||||||||||||||||||||||||||||
Sbjct  2184  AGTTAAGCCCTCGTTATGCCATTCATACGGGTCTTCATTTAAAAGACAAGTGATTACGCT  2125

Query  481   ACCTTTGCACGGTCAAAATACCGCGGCCGTTGAACTATGATGTCACTGGGCAGGCGGGAC  540
             ||||||||||||||||||||||||||||||| ||||||  ||||||||||||||||||||
Sbjct  2124  ACCTTTGCACGGTCAAAATACCGCGGCCGTTAAACTATATTGTCACTGGGCAGGCGGGAC  2065

Query  541   CT  542
             ||
Sbjct  2064  CT  2063
```

Taxonomy

Reports

- **Lineage**

Organism	Blast Name	Score	Number of Hits	Description
Teleostei	bony fishes		124	
.Pleuronectoidei	bony fishes		123	
..Paralichthys olivaceus x Verasper variegatus	bony fishes	1002	2	**Paralichthys olivaceus x Verasper variegatus hits**
..Paralichthys olivaceus	bony fishes	996	6	**Paralichthys olivaceus hits**
..Paralichthys adspersus	bony fishes	902	3	**Paralichthys adspersus hits**
..Paralichthys californicus	bony fishes	891	4	**Paralichthys californicus hits**
..Paralichthys patagonicus	bony fishes	891	3	**Paralichthys patagonicus hits**
..Paralichthys albigutta	bony fishes	872	4	**Paralichthys albigutta hits**
..Paralichthys squamilentus	bony fishes	867	1	**Paralichthys squamilentus hits**
..Paralichthys lethostigma	bony fishes	867	6	**Paralichthys lethostigma hits**
..Paralichthys dentatus	bony fishes	856	4	**Paralichthys dentatus hits**
..Ancylopsetta ommata	bony fishes	824	2	**Ancylopsetta ommata hits**
..Hippoglossina oblonga	bony fishes	802	3	**Hippoglossina oblonga hits**
..Glyptocephalus zachirus	bony fishes	802	10	**Glyptocephalus zachirus hits**
..Psettichthys melanostictus	bony fishes	802	7	**Psettichthys melanostictus hits**
..Hippoglossus stenolepis	bony fishes	802	11	**Hippoglossus stenolepis hits**
..Platichthys stellatus	bony fishes	802	8	**Platichthys stellatus hits**
..Pleuronectes platessa	bony fishes	797	7	**Pleuronectes platessa hits**
..Platichthys environmental sample	bony fishes	797	1	**Platichthys environmental sample hits**
..Lepidopsetta mochigarei	bony fishes	797	2	**Lepidopsetta mochigarei hits**
..Lepidopsetta bilineata	bony fishes	797	9	**Lepidopsetta bilineata hits**
..Lepidopsetta polyxystra	bony fishes	797	5	**Lepidopsetta polyxystra hits**
..Pseudopleuronectes americanus	bony fishes	797	3	**Pseudopleuronectes americanus hits**
..Platichthys stellatus x Verasper variegatus	bony fishes	797	2	**Platichthys stellatus x Verasper variegatus hits**
..Platichthys flesus	bony fishes	797	3	**Platichthys flesus hits**
..Verasper moseri	bony fishes	797	6	**Verasper moseri hits**
..Reinhardtius hippoglossoides	bony fishes	797	5	**Reinhardtius hippoglossoides hits**
..Dexistes rikuzenius	bony fishes	797	2	**Dexistes rikuzenius hits**

..Parophrys vetulus	bony fishes	797	1	Parophrys vetulus hits
..Glyptocephalus cynoglossus	bony fishes	797	1	Glyptocephalus cynoglossus hits
..Kareius bicoloratus	bony fishes	797	2	Kareius bicoloratus hits
.teleost environmental sample	bony fishes	1002	1	teleost environmental sample hits

- **Organism**

Description	Score	E value	Accession
Paralichthys olivaceus x Verasper variegatus [bony fishes]			
**Paralichthys olivaceus x Verasper variegatus mitochondrion, complete genome**	1002	0.0	**NC_082846**
**Paralichthys olivaceus x Verasper variegatus mitochondrion, complete genome**	1002	0.0	**OR353704**
teleost environmental sample [bony fishes]			
**Teleost environmental sample mitochondrial gene for 16S ribosomal RNA, partial sequence, clone: OTU5**	1002	0.0	**LC074456**
Paralichthys olivaceus (Japanese flounder) [bony fishes]			
**Paralichthys olivaceus mitochondrion, complete genome**	996	0.0	**NC_002386**
**Paralichthys olivaceus mitochondrial DNA, complete genome**	996	0.0	**AB028664**
**Paralichthys olivaceus 16S ribosomal RNA gene, partial sequence; mitochondrial gene for mitochondrial product**	974	0.0	**AY046953**
**Paralichthys olivaceus PO1 mitochondrial gene for 16S rRNA, partial sequence**	893	0.0	**LC647901**
**Paralichthys olivaceus PO3 mitochondrial gene for 16S rRNA, partial sequence**	889	0.0	**LC647903**
**Paralichthys olivaceus mitochondrial 16S rRNA gene, isolate 197**	837	0.0	**AM182432**
Paralichthys adspersus (fine flounder) [bony fishes]			
**Paralichthys adspersus mitochondrion, complete genome**	902	0.0	**NC_057273**
**Paralichthys adspersus mitochondrion, complete genome**	902	0.0	**MW288827**
**Paralichthys adspersus 16S ribosomal RNA gene, partial sequence; mitochondrial**	902	0.0	**HM211198**
Paralichthys californicus (California flounder) [bony fishes]			
**Paralichthys californicus mitochondrion, complete genome**	891	0.0	**MT859134**
**Paralichthys californicus voucher KU 456 16S ribosomal RNA gene, partial sequence; mitochondrial**	891	0.0	**FJ870414**
**Paralichthys californicus voucher UW:156295 16S ribosomal RNA gene, partial sequence; mitochondrial**	889	0.0	**MF134845**
**Paralichthys californicus 16S ribosomal RNA gene, partial sequence; mitochondrial**	857	0.0	**AY952499**
Paralichthys patagonicus (Patagonian flounder) [bony fishes]			
**Paralichthys patagonicus voucher DAAPV F28 16S ribosomal RNA gene, partial sequence; mitochondrial**	891	0.0	**GU324157**
**Paralichthys patagonicus 16S large subunit ribosomal RNA gene, partial sequence; mitochondrial**	891	0.0	**AY359657**
**Paralichthys patagonicus voucher LBP831 16S ribosomal RNA gene, partial sequence; mitochondrial**	891	0.0	**AY998028**
Paralichthys albigutta (Gulf flounder) [bony fishes]			
**Paralichthys albigutta voucher USNM:FISH:454751 mitochondrion, complete genome**	872	0.0	**NC_083031**
**Paralichthys albigutta voucher USNM:FISH:454751 mitochondrion, complete genome**	872	0.0	**OR546183**
**Paralichthys albigutta 16S large subunit ribosomal RNA gene, partial sequence; mitochondrial**	872	0.0	**DQ532931**
**Paralichthys albigutta 16S ribosomal RNA gene, partial sequence; mitochondrial**	867	0.0	**AY857937**
Paralichthys squamilentus (broad flounder) [bony fishes]			
**Paralichthys squamilentus voucher USNM:FISH:420936 mitochondrion, complete genome**	867	0.0	**OP057002**
Paralichthys lethostigma (southern flounder) [bony fishes]			
**Paralichthys lethostigma voucher KU 1 16S ribosomal RNA gene, partial sequence; mitochondrial**	867	0.0	**FJ870413**
**Paralichthys lethostigma clone 2 16S ribosomal RNA gene, partial sequence; mitochondrial**	867	0.0	**DQ450966**
**Paralichthys lethostigma clone 1 16S ribosomal RNA gene, partial sequence; mitochondrial**	867	0.0	**DQ450965**
**Paralichthys lethostigma mitochondrion, complete genome**	833	0.0	**NC_029223**
**Paralichthys lethostigma mitochondrion, complete genome**	833	0.0	**KT896534**
**Paralichthys lethostigma voucher IOCASFY-Plch01 16S ribosomal RNA gene, partial sequence; mitochondrial**	808	0.0	**GU248348**
Paralichthys dentatus (summer flounder) [bony fishes]			
**Paralichthys dentatus mitochondrion, complete genome**	856	0.0	**NC_029476**

Description	Score	E value	Accession
Paralichthys dentatus mitochondrion, complete genome	856	0.0	KU053334
Paralichthys dentatus voucher IOCASFY-Pdch01 16S ribosomal RNA gene, partial sequence; mitochondrial	835	0.0	GU248346
Paralichthys dentatus 16S ribosomal RNA gene, partial sequence; mitochondrial gene for mitochondrial product	832	0.0	AF488451
Ancylopsetta ommata (Gulf of Mexico ocellated flounder) [bony fishes]			
Ancylopsetta ommata voucher USNM:FISH:454750 mitochondrion, complete genome	824	0.0	NC_083030
Ancylopsetta ommata voucher USNM:FISH:454750 mitochondrion, complete genome	824	0.0	OR546182
Hippoglossina oblonga (American fourspot flounder) [bony fishes]			
Hippoglossina oblonga voucher USNM:FISH:433277 mitochondrion, complete genome	802	0.0	OP056997
Hippoglossina oblonga isolate IFPGSR large subunit ribosomal RNA gene, partial sequence; mitochondrial	797	0.0	MW945438
Paralichthys oblongus 16S ribosomal RNA gene, partial sequence; mitochondrial gene for mitochondrial product	797	0.0	AF420448
Glyptocephalus zachirus (Rex sole) [bony fishes]			
Glyptocephalus zachirus mitochondrion, partial genome	802	0.0	OR499736
Glyptocephalus zachirus voucher UW151770 16S ribosomal RNA gene, partial sequence; mitochondrial	802	0.0	MT767340
Glyptocephalus zachirus voucher UW 047669 16S ribosomal RNA gene, partial sequence; mitochondrial	802	0.0	FJ870388
Glyptocephalus zachirus voucher WTU:047669 16S ribosomal RNA gene, partial sequence; mitochondrial	802	0.0	EF119300
Glyptocephalus zachirus voucher UW:047670 mitochondrion, partial genome	797	0.0	OR575595
Glyptocephalus zachirus voucher UW155537 16S ribosomal RNA gene, partial sequence; mitochondrial	797	0.0	MT767342
Glyptocephalus zachirus voucher UW151776 16S ribosomal RNA gene, partial sequence; mitochondrial	797	0.0	MT767341
Glyptocephalus zachirus voucher UW 047670 16S ribosomal RNA gene, partial sequence; mitochondrial	797	0.0	FJ870389
Glyptocephalus zachirus voucher UW:049691 16S ribosomal RNA gene, partial sequence; mitochondrial	797	0.0	EF458435
Glyptocephalus zachirus voucher WTU:047670 16S ribosomal RNA gene, partial sequence; mitochondrial	797	0.0	EF119301
Psettichthys melanostictus (Pacific sand sole) [bony fishes]			
Psettichthys melanostictus voucher UW:47682 mitochondrion, complete genome	802	0.0	NC_082806
Psettichthys melanostictus voucher UW:47682 mitochondrion, complete genome	802	0.0	OR482564
Psettichthys melanostictus voucher WTU:047682 16S ribosomal RNA gene, partial sequence; mitochondrial	802	0.0	EF119311
Psettichthys melanostictus voucher WTU:047314 16S ribosomal RNA gene, partial sequence; mitochondrial	802	0.0	EF119260
Psettichthys melanostictus voucher UW 047314 16S ribosomal RNA gene, partial sequence; mitochondrial	797	0.0	FJ870404
Psettichthys melanostictus voucher WTU:047683 16S ribosomal RNA gene, partial sequence; mitochondrial	797	0.0	EF119312
Psettichthys melanostictus voucher WTU:047293 16S ribosomal RNA gene, partial sequence; mitochondrial	797	0.0	EF119241
Hippoglossus stenolepis (Pacific halibut) [bony fishes]			
Hippoglossus stenolepis voucher UW150612 16S ribosomal RNA gene, partial sequence; mitochondrial	802	0.0	MT767343
Hippoglossus stenolepis voucher UW157558 large subunit ribosomal RNA gene, partial sequence; mitochondrial	797	0.0	MK804647
Hippoglossus stenolepis isolate HST12-2 large subunit ribosomal RNA gene, partial sequence; mitochondrial	797	0.0	MN888916
Hippoglossus stenolepis isolate HST12-3 large subunit ribosomal RNA gene, partial sequence; mitochondrial	797	0.0	MN888915
Hippoglossus stenolepis voucher UW 048793 16S ribosomal RNA gene, partial sequence; mitochondrial	797	0.0	FJ870421
Hippoglossus stenolepis complete mitochondrial genome, isolate Hs-4	797	0.0	AM749129
Hippoglossus stenolepis complete mitochondrial genome, isolate Hs-3	797	0.0	AM749128

Description	Score	E value	Accession
Hippoglossus stenolepis complete mitochondrial genome, isolate Hs-2	797	0.0	AM749127
Hippoglossus stenolepis mitochondrion, complete genome	797	0.0	NC_009710
Hippoglossus stenolepis complete mitochondrial genome, isolate Hs-1	797	0.0	AM749126
Hippoglossus stenolepis voucher UW:048793 16S ribosomal RNA gene, partial sequence; mitochondrial	797	0.0	EF458356
Platichthys stellatus (starry flounder) [bony fishes]			
Platichthys stellatus isolate 18-07 large subunit ribosomal RNA gene, partial sequence; mitochondrial	802	0.0	MN888903
Platichthys stellatus isolate 16-07 large subunit ribosomal RNA gene, partial sequence; mitochondrial	797	0.0	MN888904
Platichthys stellatus isolate Ps2-011 large subunit ribosomal RNA gene, partial sequence; mitochondrial	797	0.0	MN888902
Platichthys stellatus isolate Ps3-011 large subunit ribosomal RNA gene, partial sequence; mitochondrial	797	0.0	MN888901
Platichthys stellatus isolate PS5-011 large subunit ribosomal RNA gene, partial sequence; mitochondrial	797	0.0	MN888899
Platichthys stellatus isolate PS6-011 large subunit ribosomal RNA gene, partial sequence; mitochondrial	797	0.0	MN888898
Platichthys stellatus voucher UW 047679 16S ribosomal RNA gene, partial sequence; mitochondrial	797	0.0	FJ870401
Platichthys stellatus voucher WTU:047679 16S ribosomal RNA gene, partial sequence; mitochondrial	797	0.0	EF119308
Pleuronectes platessa (European plaice) [bony fishes]			
Pleuronectes platessa isolate DM161a mitochondrion	797	0.0	MN122873
Pleuronectes platessa isolate F30 large subunit ribosomal RNA gene, partial sequence; mitochondrial	797	0.0	OM470926
Pleuronectes platessa voucher NRM:49437 16S ribosomal RNA gene, partial sequence; mitochondrial	797	0.0	KJ128864
Pleuronectes platessa voucher NRM:53959 16S ribosomal RNA gene, partial sequence; mitochondrial	797	0.0	KJ128865
Pleuronectes platessa voucher DAAPV F51 16S ribosomal RNA gene, partial sequence; mitochondrial	797	0.0	GU324168
Pleuronectes platessa voucher DAAPV F4 16S ribosomal RNA gene, partial sequence; mitochondrial	797	0.0	GU324137
Pleuronectes platessa 16S large subunit ribosomal RNA gene, partial sequence; mitochondrial gene for mitochondrial product	797	0.0	AY157328
Platichthys environmental sample [bony fishes]			
Platichthys environmental sample clone Pf4903 16S ribosomal RNA gene, partial sequence; mitochondrial	797	0.0	KU510499
Lepidopsetta mochigarei (dusky sole) [bony fishes]			
Lepidopsetta mochigarei isolate PKU4824 16S ribosomal RNA gene, partial sequence; mitochondrial	797	0.0	KU936350
Lepidopsetta mochigarei isolate LMO12-2 large subunit ribosomal RNA gene, partial sequence; mitochondrial	797	0.0	MN888894
Lepidopsetta bilineata (rock sole) [bony fishes]			
Lepidopsetta bilineata voucher UW:047661 mitochondrion, complete genome	797	0.0	OP035227
Lepidopsetta bilineata voucher UW:110234 mitochondrion, complete genome	797	0.0	NC_082755
Lepidopsetta bilineata voucher UW:110234 mitochondrion, complete genome	797	0.0	OR482508
Lepidopsetta bilineata voucher UW151563 16S ribosomal RNA gene, partial sequence; mitochondrial	797	0.0	MT767346
Lepidopsetta bilineata voucher UW153578 16S ribosomal RNA gene, partial sequence; mitochondrial	797	0.0	MT767345
Lepidopsetta bilineata voucher UW 048833 16S ribosomal RNA gene, partial sequence; mitochondrial	797	0.0	FJ870392
Lepidopsetta bilineata voucher WTU:047662 16S ribosomal RNA gene, partial sequence; mitochondrial	797	0.0	EF119293
Lepidopsetta bilineata voucher WTU:047661 16S ribosomal RNA gene, partial sequence; mitochondrial	797	0.0	EF119292
Lepidopsetta bilineata 16S ribosomal RNA gene, partial sequence; mitochondrial gene for mitochondrial product	795	0.0	AF488429
Lepidopsetta polyxystra (northern rock sole) [bony fishes]			
Lepidopsetta polyxystra voucher UW:48799 mitochondrion, complete genome	797	0.0	NC_082812
Lepidopsetta polyxystra voucher UW:48799 mitochondrion, complete genome	797	0.0	OR482570
Lepidopsetta polyxystra voucher UW:048789 16S ribosomal RNA gene, partial sequence; mitochondrial	797	0.0	EF458354
Lepidopsetta polyxystra voucher UW:048799 16S ribosomal RNA gene, partial sequence; mitochondrial	797	0.0	EF458361

Description	Score	E value	Accession
Lepidopsetta polyxystra voucher UW:048800 16S ribosomal RNA gene, partial sequence; mitochondrial	797	0.0	EF458362
Pseudopleuronectes americanus (winter flounder) [bony fishes]			
Pseudopleuronectes americanus voucher USNM:FISH:429777 mitochondrion, complete genome	797	0.0	NC_082555
Pseudopleuronectes americanus voucher USNM:FISH:429777 mitochondrion, complete genome	797	0.0	OR482463
Pseudopleuronectes americanus voucher KU 5419 16S ribosomal RNA gene, partial sequence; mitochondrial	797	0.0	FJ870412
Platichthys stellatus x Verasper variegatus [bony fishes]			
Platichthys stellatus x Verasper variegatus mitochondrion, complete genome	797	0.0	NC_082285
Platichthys stellatus x Verasper variegatus mitochondrion, complete genome	797	0.0	OR282488
Platichthys flesus (European flounder) [bony fishes]			
Platichthys flesus voucher NRM:49642 16S ribosomal RNA gene, partial sequence; mitochondrial	797	0.0	KJ128860
Platichthys flesus voucher NRM:47491 16S ribosomal RNA gene, partial sequence; mitochondrial	797	0.0	KJ128861
Platichthys flesus 16S large subunit ribosomal RNA gene, partial sequence; mitochondrial	797	0.0	AY359670
Verasper moseri (barfin flounder) [bony fishes]			
Verasper moseri mitochondrion, complete genome	797	0.0	LC583747
Verasper moseri voucher UW 118096 16S ribosomal RNA gene, partial sequence; mitochondrial	797	0.0	FJ870415
Verasper moseri mitochondrion, complete genome	797	0.0	NC_008461
Verasper moseri mitochondrion, complete genome	797	0.0	EF025506
Verasper moseri 16S ribosomal RNA gene, complete sequence; mitochondrial	797	0.0	DQ834443
Verasper moseri 16S ribosomal RNA gene, partial sequence; mitochondrial	797	0.0	DQ242489
Reinhardtius hippoglossoides (Greenland flounder) [bony fishes]			
Reinhardtius hippoglossoides voucher UW 114782 16S ribosomal RNA gene, partial sequence; mitochondrial	797	0.0	FJ870422
Reinhardtius hippoglossoides complete mitochondrial genome, isolate Rh-4	797	0.0	AM749133
Reinhardtius hippoglossoides complete mitochondrial genome, isolate Rh-2	797	0.0	AM749131
Reinhardtius hippoglossoides mitochondrion, complete genome	797	0.0	NC_009711
Reinhardtius hippoglossoides complete mitochondrial genome, isolate Rh-1	797	0.0	AM749130
Dexistes rikuzenius (Rikuzen flounder) [bony fishes]			
Dexistes rikuzenius voucher FAKU 131267 16S ribosomal RNA gene, partial sequence; mitochondrial	797	0.0	FJ870420
Dexistes rikuzenius voucher FAKU 131266 16S ribosomal RNA gene, partial sequence; mitochondrial	797	0.0	FJ870419
Parophrys vetulus (English sole) [bony fishes]			
Parophrys vetulus voucher UW 047297 16S ribosomal RNA gene, partial sequence; mitochondrial	797	0.0	FJ870399
Glyptocephalus cynoglossus (witch) [bony fishes]			
Glyptocephalus cynoglossus 16S ribosomal RNA gene, partial sequence; mitochondrial gene for mitochondrial product	797	0.0	AF420447
Kareius bicoloratus (stone flounder) [bony fishes]			
Kareius bicoloratus mitochondrion, complete genome	797	0.0	NC_003176
Kareius bicoloratus mitochondrial DNA, complete genome, except for D-loop	797	0.0	AP002951

- ## Taxonomy

Taxonomy	Number of hits	Number of Organisms	Description
Teleostei	124	30	
. Pleuronectoidei	123	29	
.. Paralichthys olivaceus x Verasper variegatus	2	1	Paralichthys olivaceus x Verasper variegatus hits
.. Paralichthyidae	36	10	
... Paralichthys	31	8	
.... Paralichthys olivaceus	6	1	Paralichthys olivaceus hits
.... Paralichthys adspersus	3	1	Paralichthys adspersus hits
.... Paralichthys californicus	4	1	Paralichthys californicus hits
.... Paralichthys patagonicus	3	1	Paralichthys patagonicus hits

.... Paralichthys albigutta	4	1	Paralichthys albigutta hits
.... Paralichthys squamilentus	1	1	Paralichthys squamilentus hits
.... Paralichthys lethostigma	6	1	Paralichthys lethostigma hits
.... Paralichthys dentatus	4	1	Paralichthys dentatus hits
... Ancylopsetta ommata	2	1	Ancylopsetta ommata hits
... Hippoglossina oblonga	3	1	Hippoglossina oblonga hits
.. Pleuronectidae	85	18	
... Glyptocephalus	11	2	
.... Glyptocephalus zachirus	10	1	Glyptocephalus zachirus hits
.... Glyptocephalus cynoglossus	1	1	Glyptocephalus cynoglossus hits
... Psettichthys melanostictus	7	1	Psettichthys melanostictus hits
... Hippoglossus stenolepis	11	1	Hippoglossus stenolepis hits
... Platichthys	14	4	
.... Platichthys stellatus	8	1	Platichthys stellatus hits
.... Platichthys environmental sample	1	1	Platichthys environmental sample hits
.... Platichthys stellatus x Verasper variegatus	2	1	Platichthys stellatus x Verasper variegatus hits
.... Platichthys flesus	3	1	Platichthys flesus hits
... Pleuronectes platessa	7	1	Pleuronectes platessa hits
... Lepidopsetta	16	3	
.... Lepidopsetta mochigarei	2	1	Lepidopsetta mochigarei hits
.... Lepidopsetta bilineata	9	1	Lepidopsetta bilineata hits
.... Lepidopsetta polyxystra	5	1	Lepidopsetta polyxystra hits
... Pseudopleuronectes americanus	3	1	Pseudopleuronectes americanus hits
... Verasper moseri	6	1	Verasper moseri hits
... Reinhardtius hippoglossoides	5	1	Reinhardtius hippoglossoides hits
... Dexistes rikuzenius	2	1	Dexistes rikuzenius hits
... Parophrys vetulus	1	1	Parophrys vetulus hits
... Kareius bicoloratus	2	1	Kareius bicoloratus hits
. teleost environmental sample	1	1	teleost environmental sample hits

Top

Follow NCBI

Connect with NLM

National Library of Medicine
8600 Rockville Pike
Bethesda, MD 20894

Web Policies
FOIA
HHS Vulnerability Disclosure

Help
Accessibility
Careers

- NLM
- NIH
- HHS
- USA.gov
