# Supplementary material for: Establishment and Characterization of OFT and OFO Cell Lines from Olive Flounder (Paralichthys olivaceus) for Use as Feeder Cells
Source: Biology (Basel). 2025 Feb 24;14(3):229. doi: 10.3390/biology14030229 (PMC11939788; doi:10.3390/biology14030229)
Supplement: Supplementary file 1 [file biology-14-00229-s001.zip › NCBI Blast_COI sequencing_OFO.pdf]

An official website of the United States government

**Here's how you know**

**The .gov means it’s official.**
Federal government websites often end in .gov or .mil. Before sharing sensitive information, make sure you’re on a federal government site.

Feedback

**The site is secure.**
The **https://** ensures that you are connecting to the official website and that any information you provide is encrypted and transmitted securely.
Access keys NCBI Homepage MyNCBI Homepage Main Content Main Navigation

## BLAST ® » blastn suite » results for RID-09E1SU9T013

| | |
|---|---|
| **Job Title** | FISHR2-01.ab1 ... |
| **RID** | 09E1SU9T013 Search expires on 03-29 08:22 am |
| **Results for** | 4:lcl\|Query_20007 VF2-02.ab1(650bp) |
| **Program** | BLASTN |
| **Database** | nt |
| **Query ID** | lcl\|Query_20007 |
| **Description** | VF2-02.ab1 ... |
| **Molecule type** | dna |
| **Query Length** | 650 |

## Descriptions

| Description | Scientific Name | Max Score | Total Score | Query Cover | E value | Per. Ident | Acc. Len | Accession |
|---|---|---|---|---|---|---|---|---|
| Paralichthys olivaceus x Verasper variegatus mitochondrion, complete genome | Paralichthys olivaceus x Verasper variegatus | 1201 | 1201 | 100% | 0.0 | 100.00% | 16946 | NC_082846.1 |
| Paralichthys olivaceus voucher XM073 cytochrome oxidase subunit I (COI) gene, partial cds; mitochondrial | Paralichthys olivaceus | 1201 | 1201 | 100% | 0.0 | 100.00% | 655 | KX254476.1 |
| Paralichthys olivaceus voucher HME1 cytochrome oxidase subunit 1 (COI) gene, partial cds; mitochondrial | Paralichthys olivaceus | 1201 | 1201 | 100% | 0.0 | 100.00% | 652 | JF952803.1 |
| Paralichthys olivaceus isolate CO1B7 cytochrome oxidase subunit I-like (COI) gene, partial sequence; mitochondrial | Paralichthys olivaceus | 1201 | 1201 | 100% | 0.0 | 100.00% | 713 | EU266369.1 |
| Paralichthys olivaceus voucher HUM I-00297 cytochrome c oxidase subunit 1 (COI) gene, complete cds; mitochondrial | Paralichthys olivaceus | 1195 | 1195 | 100% | 0.0 | 99.85% | 1551 | MH032483.1 |
| Paralichthys olivaceus voucher XM072 cytochrome oxidase subunit I (COI) gene, partial cds; mitochondrial | Paralichthys olivaceus | 1195 | 1195 | 100% | 0.0 | 99.85% | 655 | KX254475.1 |
| Paralichthys olivaceus voucher HUM I-00296 cytochrome c oxidase subunit 1 (COI) gene, complete cds; mitochondrial | Paralichthys olivaceus | 1190 | 1190 | 100% | 0.0 | 99.69% | 1551 | MH032482.1 |
| Paralichthys olivaceus voucher XM074 cytochrome oxidase subunit I (COI) gene, partial cds; mitochondrial | Paralichthys olivaceus | 1190 | 1190 | 100% | 0.0 | 99.69% | 655 | KX254477.1 |
| Paralichthys olivaceus voucher HME2 cytochrome oxidase subunit 1 (COI) gene, partial cds; mitochondrial | Paralichthys olivaceus | 1190 | 1190 | 100% | 0.0 | 99.69% | 652 | JF952804.1 |
| Paralichthys olivaceus isolate CO1B8 cytochrome oxidase subunit I (COI) gene, partial cds; mitochondrial | Paralichthys olivaceus | 1190 | 1190 | 100% | 0.0 | 99.69% | 710 | EU266368.1 |
| Paralichthys olivaceus mitochondrion, complete genome | Paralichthys olivaceus | 1190 | 1190 | 100% | 0.0 | 99.69% | 17090 | NC_002386.1 |
| Paralichthys olivaceus isolate WJC173 cytochrome oxidase subunit 1 (COI) gene, partial cds; mitochondrial | Paralichthys olivaceus | 1173 | 1173 | 98% | 0.0 | 99.69% | 642 | MK617161.1 |
| Paralichthys olivaceus strain West.5.3 cytochrome c | Paralichthys olivaceus | 1173 | 1173 | 99% | 0.0 | 99.38% | 647 | MK560569.1 |

| Description | Scientific Name | Max Score | Total Score | Query Cover | E value | Per. Ident | Acc. Len | Accession |
|---|---|---|---|---|---|---|---|---|
| oxidase subunit I (COI) gene, partial cds; mitochondrial | | | | | | | | |
| Paralichthys olivaceus voucher IOCASFY-RCB09-Po5 cytochrome oxidase subunit I (COI) gene, partial cds; mitochondrial | Paralichthys olivaceus | 1146 | 1146 | 95% | 0.0 | 100.00% | 620 | KU236831.1 |
| Paralichthys olivaceus voucher IOCASFY-RCB09-Po1 cytochrome oxidase subunit I (COI) gene, partial cds; mitochondrial | Paralichthys olivaceus | 1134 | 1134 | 95% | 0.0 | 99.68% | 620 | KU236827.1 |
| Paralichthys olivaceus voucher IOCASFY-RCB09-Po7 cytochrome oxidase subunit I (COI) gene, partial cds; mitochondrial | Paralichthys olivaceus | 1129 | 1129 | 95% | 0.0 | 99.52% | 620 | KU236833.1 |
| Paralichthys olivaceus isolate j4 cytochrome c oxidase subunit I (COX1) gene, partial cds; mitochondrial | Paralichthys olivaceus | 1109 | 1109 | 93% | 0.0 | 99.51% | 617 | MZ317453.1 |
| Paralichthys olivaceus isolate PKU_10872 cytochrome oxidase subunit I (COI) gene, partial cds; mitochondrial | Paralichthys olivaceus | 1101 | 1101 | 92% | 0.0 | 99.67% | 604 | KP835312.1 |
| Paralichthys olivaceus isolate ASIZP0914829 cytochrome oxidase subunit I (COI) gene, partial cds; mitochondrial | Paralichthys olivaceus | 1003 | 1003 | 84% | 0.0 | 99.46% | 552 | KU945107.1 |
| Paralichthys olivaceus isolate F00223 cytochrome oxidase subunit I (COI) gene, partial cds; mitochondrial | Paralichthys olivaceus | 1002 | 1002 | 92% | 0.0 | 96.83% | 612 | JQ738445.1 |
| Paralichthys olivaceus isolate sample_77 cytochrome c oxidase subunit I (COI) gene, partial cds; mitochondrial | Paralichthys olivaceus | 965 | 965 | 80% | 0.0 | 100.00% | 522 | MW027188.1 |
| Paralichthys olivaceus isolate PKU 4489 cytochrome oxidase subunit I (COI) gene, partial cds; mitochondrial | Paralichthys olivaceus | 946 | 946 | 79% | 0.0 | 99.81% | 515 | KR052269.1 |
| Paralichthys olivaceus mitochondrial COX1 gene for cytochrome c oxidase subunit 1, partial cds, isolate: 005_55 | Paralichthys olivaceus | 915 | 915 | 77% | 0.0 | 99.60% | 501 | LC126342.1 |
| Paralichthys olivaceus isolate PKU 4490 cytochrome oxidase subunit I gene, | Paralichthys olivaceus | 843 | 843 | 70% | 0.0 | 100.00% | 456 | KF965424.1 |

| Description | Scientific Name | Max Score | Total Score | Query Cover | E value | Per. Ident | Acc. Len | Accession |
|---|---|---|---|---|---|---|---|---|
| partial cds; mitochondrial | | | | | | | | |
| Paralichthys olivaceus isolate PKU 4489 cytochrome oxidase subunit I gene, partial cds; mitochondrial | Paralichthys olivaceus | 837 | 837 | 70% | 0.0 | 99.78% | 456 | KF965438.1 |
| Paralichthys aestuarius voucher SIO 07-157 cytochrome c oxidase subunit 1 (COI) gene, complete cds; mitochondrial | Paralichthys aestuarius | 819 | 819 | 100% | 0.0 | 89.38% | 1551 | MH032480.1 |
| Paralichthys olivaceus isolate F00222 cytochrome oxidase subunit I (COI) gene, partial cds; mitochondrial | Paralichthys olivaceus | 813 | 813 | 68% | 0.0 | 99.55% | 448 | JQ738444.1 |
| Paralichthys squamilentus voucher KUT 5205 cytochrome oxidase subunit 1 (COI) gene, partial cds; mitochondrial | Paralichthys squamilentus | 808 | 808 | 99% | 0.0 | 89.18% | 652 | KF930230.1 |
| Paralichthys patagonicus voucher HRCB:53035 cytochrome oxidase subunit 1 (COI) gene, partial cds; mitochondrial | Paralichthys patagonicus | 808 | 808 | 100% | 0.0 | 89.08% | 652 | JQ365477.1 |
| Paralichthys adspersus mitochondrion, complete genome | Paralichthys adspersus | 802 | 802 | 100% | 0.0 | 88.92% | 17060 | NC_057273.1 |
| Paralichthys patagonicus voucher DAAPV F28 cytochrome oxidase subunit I (COI) gene, partial cds; mitochondrial | Paralichthys patagonicus | 798 | 798 | 99% | 0.0 | 88.89% | 652 | GU324191.1 |
| Paralichthys patagonicus voucher HRCB:53034 cytochrome oxidase subunit 1 (COI) gene, partial cds; mitochondrial | Paralichthys patagonicus | 787 | 787 | 99% | 0.0 | 88.58% | 652 | JQ365478.1 |
| Paralichthys californicus voucher SIO 03-51 cytochrome c oxidase subunit 1 (COI) gene, complete cds; mitochondrial | Paralichthys californicus | 774 | 774 | 100% | 0.0 | 88.15% | 1551 | MH032481.1 |
| Paralichthys californicus mitochondrion, complete genome | Paralichthys californicus | 763 | 763 | 100% | 0.0 | 87.85% | 16858 | MT859134.1 |
| Paralichthys californicus voucher MFC144 cytochrome oxidase subunit 1 (COI) gene, partial cds; mitochondrial | Paralichthys californicus | 763 | 763 | 100% | 0.0 | 87.85% | 652 | GU440447.1 |
| Paralichthys dentatus voucher USNM:FISH:431132 cytochrome oxidase subunit 1 (COI) gene, | Paralichthys dentatus | 734 | 734 | 99% | 0.0 | 87.06% | 655 | MT455401.1 |

| Description | Scientific Name | Max Score | Total Score | Query Cover | E value | Per. Ident | Acc. Len | Accession |
|---|---|---|---|---|---|---|---|---|
| partial cds; mitochondrial | | | | | | | | |
| Actinopterygii environmental sample voucher DE1109058_05 cytochrome oxidase subunit 1 (COI) gene, partial cds; mitochondrial | Actinopterygii environmental sample | 734 | 734 | 99% | 0.0 | 87.06% | 652 | KP110988.1 |
| Paralichthys dentatus voucher 07-045 cytochrome oxidase subunit 1 (COI) gene, partial cds; mitochondrial | Paralichthys dentatus | 734 | 734 | 99% | 0.0 | 87.06% | 652 | KC015757.1 |
| Paralichthys dentatus voucher 07-123 cytochrome oxidase subunit 1 (COI) gene, partial cds; mitochondrial | Paralichthys dentatus | 730 | 730 | 99% | 0.0 | 86.90% | 652 | KC015758.1 |
| Paralichthys dentatus voucher USNM:FISH:423822 cytochrome oxidase subunit 1 (COI) gene, partial cds; mitochondrial | Paralichthys dentatus | 728 | 728 | 99% | 0.0 | 86.90% | 655 | MT455243.1 |
| Paralichthys woolmani voucher SIO-09-205 cytochrome c oxidase subunit 1 (COI) gene, complete cds; mitochondrial | Paralichthys woolmani | 728 | 728 | 99% | 0.0 | 86.90% | 1551 | MH032484.1 |
| Paralichthys dentatus mitochondrion, complete genome | Paralichthys dentatus | 728 | 728 | 99% | 0.0 | 86.90% | 17033 | NC_029476.1 |
| Actinopterygii environmental sample voucher DE1109058_01 cytochrome oxidase subunit 1 (COI) gene, partial cds; mitochondrial | Actinopterygii environmental sample | 728 | 728 | 99% | 0.0 | 86.90% | 652 | KP111879.1 |
| Paralichthys dentatus voucher 06-117 cytochrome oxidase subunit 1 (COI) gene, partial cds; mitochondrial | Paralichthys dentatus | 728 | 728 | 99% | 0.0 | 86.90% | 652 | KC015755.1 |
| Paralichthys dentatus voucher 09-400 cytochrome oxidase subunit 1 (COI) gene, partial cds; mitochondrial | Paralichthys dentatus | 728 | 728 | 99% | 0.0 | 86.90% | 652 | KC015756.1 |
| Paralichthys dentatus voucher USNM:FISH:423832 cytochrome oxidase subunit 1 (COI) gene, partial cds; mitochondrial | Paralichthys dentatus | 725 | 725 | 99% | 0.0 | 86.86% | 649 | MT456241.1 |
| Actinopterygii environmental sample voucher DL0706_054_04 cytochrome oxidase subunit 1 (COI) gene, partial cds; mitochondrial | Actinopterygii environmental sample | 725 | 725 | 99% | 0.0 | 86.96% | 646 | KP111797.1 |

| Description | Scientific Name | Max Score | Total Score | Query Cover | E value | Per. Ident | Acc. Len | Accession |
|---|---|---|---|---|---|---|---|---|
| Paralichthys albigutta voucher FDA 103 cytochrome oxidase subunit 1 (COI) gene, partial cds; mitochondrial | Paralichthys albigutta | 725 | 725 | 99% | 0.0 | 86.86% | 655 | KF461215.1 |
| Paralichthys dentatus voucher USNM:FISH:423933 cytochrome oxidase subunit 1 (COI) gene, partial cds; mitochondrial | Paralichthys dentatus | 723 | 723 | 99% | 0.0 | 86.75% | 655 | MT455927.1 |
| Paralichthys dentatus voucher USNM:FISH:423811 cytochrome oxidase subunit 1 (COI) gene, partial cds; mitochondrial | Paralichthys dentatus | 723 | 723 | 99% | 0.0 | 86.84% | 648 | MT455323.1 |
| Paralichthys albigutta voucher MXV0035 cytochrome oxidase subunit 1 (COI) gene, partial cds; mitochondrial | Paralichthys albigutta | 723 | 723 | 99% | 0.0 | 86.75% | 652 | MG837970.1 |
| Actinopterygii environmental sample voucher DE1109067_03 cytochrome oxidase subunit 1 (COI) gene, partial cds; mitochondrial | Actinopterygii environmental sample | 723 | 723 | 99% | 0.0 | 86.75% | 652 | KP111488.1 |
| Actinopterygii environmental sample voucher DL0706_054_03 cytochrome oxidase subunit 1 (COI) gene, partial cds; mitochondrial | Actinopterygii environmental sample | 721 | 721 | 99% | 0.0 | 86.82% | 647 | KP110873.1 |
| Paralichthys albigutta voucher USNM:FISH:447358 cytochrome oxidase subunit 1 (COI) gene, partial cds; mitochondrial | Paralichthys albigutta | 719 | 719 | 99% | 0.0 | 86.71% | 655 | MT455829.1 |
| Paralichthys albigutta voucher USNM:FISH:454751 mitochondrion, complete genome | Paralichthys albigutta | 719 | 719 | 99% | 0.0 | 86.71% | 17035 | NC_083031.1 |
| Paralichthys orbignyanus isolate FARG322-07 cytochrome oxidase subunit I (COI) gene, partial cds; mitochondrial | Paralichthys orbignyanus | 719 | 719 | 99% | 0.0 | 86.71% | 652 | EU074519.1 |
| Actinopterygii environmental sample voucher DE0210_011_05 cytochrome oxidase subunit 1 (COI) gene, partial cds; mitochondrial | Actinopterygii environmental sample | 715 | 715 | 98% | 0.0 | 86.76% | 644 | KP111710.1 |
| Paralichthys albigutta voucher USNM:FISH:451250 cytochrome oxidase subunit 1 (COI) gene, partial cds; mitochondrial | Paralichthys albigutta | 713 | 713 | 99% | 0.0 | 86.55% | 655 | MT455012.1 |

| Description | Scientific Name | Max Score | Total Score | Query Cover | E value | Per. Ident | Acc. Len | Accession |
|---|---|---|---|---|---|---|---|---|
| Actinopterygii environmental sample voucher DE0210_011_02 cytochrome oxidase subunit 1 (COI) gene, partial cds; mitochondrial | Actinopterygii environmental sample | 712 | 712 | 98% | 0.0 | 86.72% | 642 | KP111827.1 |
| Actinopterygii environmental sample voucher DE0210_011_06 cytochrome oxidase subunit 1 (COI) gene, partial cds; mitochondrial | Actinopterygii environmental sample | 712 | 712 | 98% | 0.0 | 86.72% | 642 | KP111283.1 |
| Actinopterygii environmental sample voucher DE0210_002_02 cytochrome oxidase subunit 1 (COI) gene, partial cds; mitochondrial | Actinopterygii environmental sample | 712 | 712 | 98% | 0.0 | 86.72% | 642 | KP111187.1 |
| Actinopterygii environmental sample voucher DE0210_011_03 cytochrome oxidase subunit 1 (COI) gene, partial cds; mitochondrial | Actinopterygii environmental sample | 710 | 710 | 97% | 0.0 | 86.89% | 635 | KP111956.1 |
| Actinopterygii environmental sample voucher DE0210_011_01 cytochrome oxidase subunit 1 (COI) gene, partial cds; mitochondrial | Actinopterygii environmental sample | 710 | 710 | 98% | 0.0 | 86.70% | 641 | KP111944.1 |
| Actinopterygii environmental sample voucher DE0911_037_01 cytochrome oxidase subunit 1 (COI) gene, partial cds; mitochondrial | Actinopterygii environmental sample | 710 | 710 | 97% | 0.0 | 86.89% | 635 | KP111360.1 |
| Actinopterygii environmental sample voucher DL0711_005_07 cytochrome oxidase subunit 1 (COI) gene, partial cds; mitochondrial | Actinopterygii environmental sample | 710 | 710 | 97% | 0.0 | 86.89% | 635 | KP111091.1 |
| Actinopterygii environmental sample voucher DE0210_011_07 cytochrome oxidase subunit 1 (COI) gene, partial cds; mitochondrial | Actinopterygii environmental sample | 710 | 710 | 98% | 0.0 | 86.60% | 644 | KP110799.1 |
| Paralichthys dentatus voucher 06-099 cytochrome oxidase subunit 1 (COI) gene, partial cds; mitochondrial | Paralichthys dentatus | 710 | 710 | 99% | 0.0 | 86.24% | 652 | KC015754.1 |
| Actinopterygii environmental sample voucher DL0711_005_05 cytochrome oxidase subunit 1 (COI) gene, partial cds; mitochondrial | Actinopterygii environmental sample | 706 | 706 | 96% | 0.0 | 87.06% | 629 | KP111700.1 |

| Description | Scientific Name | Max Score | Total Score | Query Cover | E value | Per. Ident | Acc. Len | Accession |
|---|---|---|---|---|---|---|---|---|
| Actinopterygii environmental sample voucher AL0410_053_01 cytochrome oxidase subunit 1 (COI) gene, partial cds; mitochondrial | Actinopterygii environmental sample | 706 | 706 | 96% | 0.0 | 87.06% | 633 | KP111235.1 |
| Paralichthyidae sp. BOLD:AAO1988 voucher SMSA7168 cytochrome oxidase subunit 1 (COI) gene, partial cds; mitochondrial | Paralichthyidae sp. BOLD:AAO1988 | 706 | 706 | 98% | 0.0 | 86.66% | 639 | JQ842632.1 |
| Actinopterygii environmental sample voucher DL0711_034_01 cytochrome oxidase subunit 1 (COI) gene, partial cds; mitochondrial | Actinopterygii environmental sample | 704 | 704 | 97% | 0.0 | 86.73% | 635 | KP111750.1 |
| Actinopterygii environmental sample voucher DL0711_005_06 cytochrome oxidase subunit 1 (COI) gene, partial cds; mitochondrial | Actinopterygii environmental sample | 704 | 704 | 97% | 0.0 | 86.73% | 635 | KP111041.1 |
| Actinopterygii environmental sample voucher DL0711_056_08 cytochrome oxidase subunit 1 (COI) gene, partial cds; mitochondrial | Actinopterygii environmental sample | 704 | 704 | 97% | 0.0 | 86.73% | 635 | KP110830.1 |
| Actinopterygii environmental sample voucher AL0410_028_04 cytochrome oxidase subunit 1 (COI) gene, partial cds; mitochondrial | Actinopterygii environmental sample | 702 | 702 | 97% | 0.0 | 86.71% | 634 | KP111841.1 |
| Actinopterygii environmental sample voucher AL0410_028_09 cytochrome oxidase subunit 1 (COI) gene, partial cds; mitochondrial | Actinopterygii environmental sample | 702 | 702 | 97% | 0.0 | 86.71% | 634 | KP110854.1 |
| Actinopterygii environmental sample voucher DL0711_056_01 cytochrome oxidase subunit 1 (COI) gene, partial cds; mitochondrial | Actinopterygii environmental sample | 701 | 701 | 96% | 0.0 | 86.90% | 631 | KP111741.1 |
| Actinopterygii environmental sample voucher DL0711_056_03 cytochrome oxidase subunit 1 (COI) gene, partial cds; mitochondrial | Actinopterygii environmental sample | 701 | 701 | 96% | 0.0 | 86.90% | 627 | KP111179.1 |
| Actinopterygii environmental sample voucher DL0711_056_07 cytochrome oxidase subunit 1 (COI) gene, | Actinopterygii environmental sample | 701 | 701 | 96% | 0.0 | 86.90% | 630 | KP111103.1 |

| Description | Scientific Name | Max Score | Total Score | Query Cover | E value | Per. Ident | Acc. Len | Accession |
|---|---|---|---|---|---|---|---|---|
| partial cds; mitochondrial | | | | | | | | |
| Actinopterygii environmental sample voucher AL0410_028_03 cytochrome oxidase subunit 1 (COI) gene, partial cds; mitochondrial | Actinopterygii environmental sample | 699 | 699 | 97% | 0.0 | 86.55% | 634 | KP112075.1 |
| Actinopterygii environmental sample voucher DL0711_056_06 cytochrome oxidase subunit 1 (COI) gene, partial cds; mitochondrial | Actinopterygii environmental sample | 699 | 699 | 97% | 0.0 | 86.57% | 635 | KP111884.1 |
| Actinopterygii environmental sample voucher DL0711_005_02 cytochrome oxidase subunit 1 (COI) gene, partial cds; mitochondrial | Actinopterygii environmental sample | 697 | 697 | 96% | 0.0 | 86.74% | 627 | KP111731.1 |
| Actinopterygii environmental sample voucher DE0210_011_04 cytochrome oxidase subunit 1 (COI) gene, partial cds; mitochondrial | Actinopterygii environmental sample | 697 | 697 | 96% | 0.0 | 86.74% | 633 | KP111115.1 |
| Paralichthys woolmani isolate LPZSF105 cytochrome c oxidase subunit I (COX1) gene, partial cds; mitochondrial | Paralichthys woolmani | 693 | 693 | 93% | 0.0 | 87.29% | 606 | MT311625.1 |
| Xystreurys rasile isolate FARG358-07 cytochrome oxidase subunit I (COI) gene, partial cds; mitochondrial | Xystreurys rasile | 691 | 691 | 99% | 0.0 | 85.94% | 652 | EU074623.1 |
| Paralichthys dentatus cytochrome oxidase subunit I (COI) gene, partial cds; mitochondrial | Paralichthys dentatus | 686 | 686 | 96% | 0.0 | 86.31% | 629 | KX164002.1 |
| Paralichthys californicus voucher JUPA-822 cytochrome oxidase subunit 1 (COI) gene, partial cds; mitochondrial | Paralichthys californicus | 686 | 686 | 93% | 0.0 | 87.11% | 605 | KM077536.1 |
| Paralichthys isosceles voucher HRCB:46871 cytochrome oxidase subunit 1 (COI) gene, partial cds; mitochondrial | Paralichthys isosceles | 686 | 686 | 99% | 0.0 | 85.78% | 652 | JQ365476.1 |
| Xystreurys rasile isolate FARG218-06 cytochrome oxidase subunit I (COI) gene, partial cds; mitochondrial | Xystreurys rasile | 686 | 686 | 99% | 0.0 | 85.78% | 652 | EU074629.1 |
| Xystreurys rasile isolate FARG220-06 cytochrome oxidase subunit I (COI) gene, | Xystreurys rasile | 686 | 686 | 99% | 0.0 | 85.78% | 652 | EU074627.1 |

| Description | Scientific Name | Max Score | Total Score | Query Cover | E value | Per. Ident | Acc. Len | Accession |
|---|---|---|---|---|---|---|---|---|
| partial cds; mitochondrial | | | | | | | | |
| Xystreurys rasile isolate FARG217-06 cytochrome oxidase subunit I (COI) gene, partial cds; mitochondrial | Xystreurys rasile | 686 | 686 | 99% | 0.0 | 85.78% | 652 | EU074624.1 |
| Paralichthys californicus voucher JUPA-823 cytochrome oxidase subunit 1 (COI) gene, partial cds; mitochondrial | Paralichthys californicus | 682 | 682 | 92% | 0.0 | 87.06% | 603 | KM019390.1 |
| Paralichthys californicus voucher JUPA-827 cytochrome oxidase subunit 1 (COI) gene, partial cds; mitochondrial | Paralichthys californicus | 682 | 682 | 92% | 0.0 | 87.17% | 602 | KM019385.1 |
| Litopenaeus vannamei voucher DAAPV F14 cytochrome oxidase subunit I (COI) gene, partial cds; mitochondrial | Penaeus vannamei | 680 | 680 | 99% | 0.0 | 85.63% | 652 | GU324180.1 |
| Xystreurys rasile isolate FARG219-06 cytochrome oxidase subunit I (COI) gene, partial cds; mitochondrial | Xystreurys rasile | 680 | 680 | 99% | 0.0 | 85.63% | 652 | EU074626.1 |
| Xystreurys rasile isolate FARG216-06 cytochrome oxidase subunit I (COI) gene, partial cds; mitochondrial | Xystreurys rasile | 680 | 680 | 99% | 0.0 | 85.63% | 652 | EU074625.1 |
| Xystreurys rasile isolate FARG359-07 cytochrome oxidase subunit I (COI) gene, partial cds; mitochondrial | Xystreurys rasile | 680 | 680 | 99% | 0.0 | 85.63% | 652 | EU074622.1 |
| Verasper moseri voucher UW 118097 cytochrome c oxidase subunit 1 (COI) gene, complete cds; mitochondrial | Verasper moseri | 678 | 678 | 99% | 0.0 | 85.60% | 1557 | MH032541.1 |
| Verasper moseri voucher UW 118096 cytochrome c oxidase subunit 1 (COI) gene, complete cds; mitochondrial | Verasper moseri | 678 | 678 | 99% | 0.0 | 85.60% | 1557 | MH032540.1 |
| Verasper moseri mitochondrion, complete genome | Verasper moseri | 678 | 678 | 99% | 0.0 | 85.60% | 17443 | LC583747.1 |
| Verasper moseri mitochondrion, complete genome | Verasper moseri | 678 | 678 | 99% | 0.0 | 85.60% | 17588 | NC_008461.1 |

## Graphic Summary

**Distribution of the top 100 Blast Hits on 100 subject sequences**

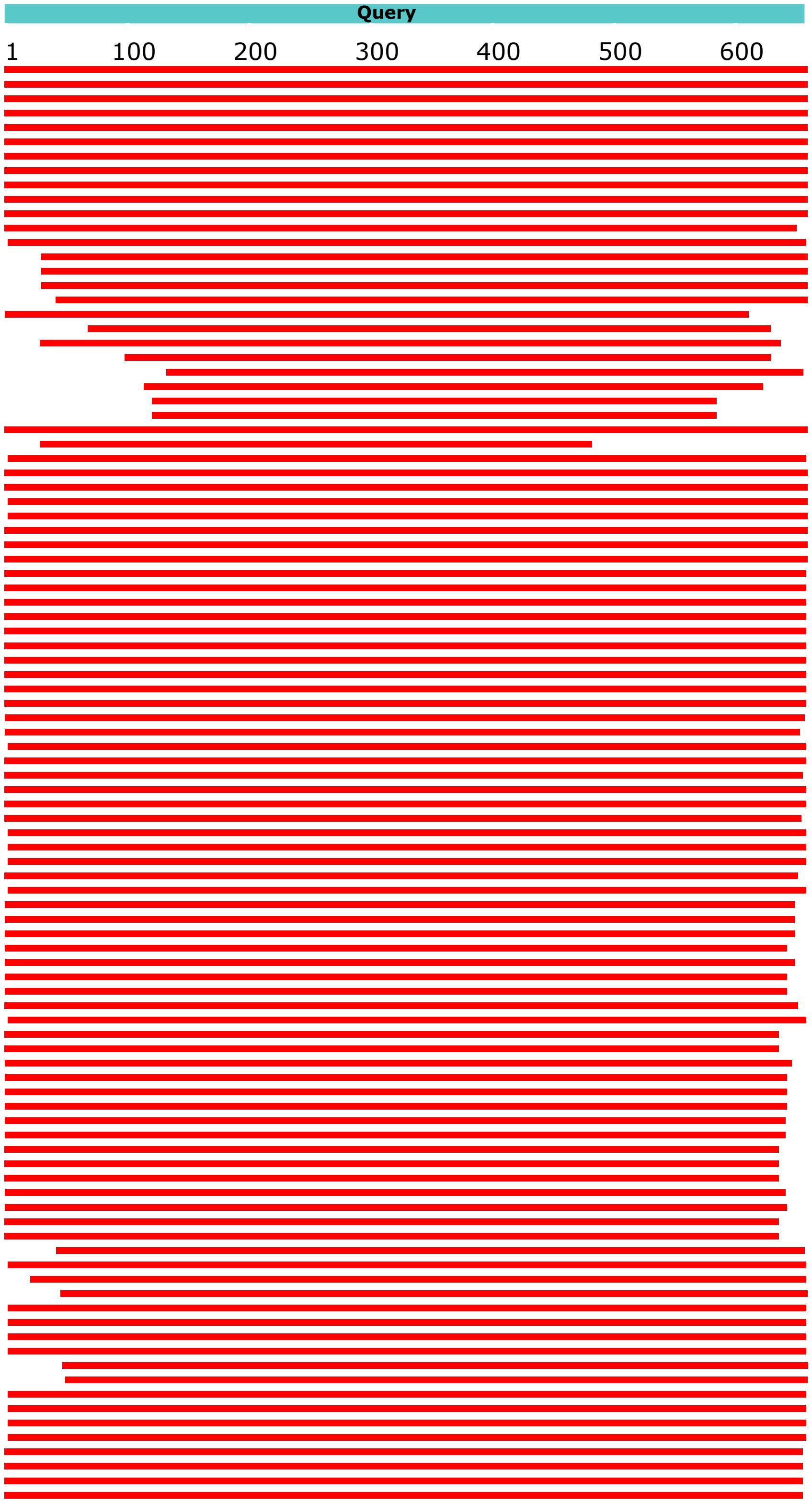


## Alignments

Alignment view Pairwise ☐ CDS feature Restore defaults

Paralichthys olivaceus x Verasper variegatus mitochondrion, complete genome
Sequence ID: **NC_082846.1** Length: 16946 Number of Matches: 1
Range 1: 5552 to 6201

| Score | Expect | Identities | Gaps | Strand | Frame |
|---|---|---|---|---|---|
| 1201 bits(650) | 0.0() | 650/650(100%) | 0/650(0%) | Plus/Plus | |

```
Query  1     TCTATCTCGTATTTGGTGCCTGAGCCGGAATAGTGGGGACAGCCCTAAGCCTCCTCATTC  60
             ||||||||||||||||||||||||||||||||||||||||||||||||||||||||||||
Sbjct  5552  TCTATCTCGTATTTGGTGCCTGAGCCGGAATAGTGGGGACAGCCCTAAGCCTCCTCATTC  5611

Query  61    GGGCAGAACTCAGCCAACCTGGTGCTCTCCTAGGGGACGACCAGATTTATAACGTAATCG  120
             ............................................................
```

```
                  ||||||||||||||||||||||||||||||||||||||||||||||||||||||||||||
Sbjct  5612  GGGCAGAACTCAGCCAACCTGGTGCTCTCCTAGGGGACGACCAGATTTATAACGTAATCG  5671

Query  121   TTACCGCACACGCCTTTGTAATAATCTTTTTCATAGTTATACCAATTATGATTGGAGGCT  180
             ||||||||||||||||||||||||||||||||||||||||||||||||||||||||||||
Sbjct  5672  TTACCGCACACGCCTTTGTAATAATCTTTTTCATAGTTATACCAATTATGATTGGAGGCT  5731

Query  181   TTGGCAACTGACTTATCCCCCTGATAATCGGTGCCCCAGACATAGCATTCCCTCGAATAA  240
             ||||||||||||||||||||||||||||||||||||||||||||||||||||||||||||
Sbjct  5732  TTGGCAACTGACTTATCCCCCTGATAATCGGTGCCCCAGACATAGCATTCCCTCGAATAA  5791

Query  241   ATAACATAAGCTTCTGACTTCTACCCCCTTCATTCCTTCTTCTCCTGGCTTCTTCAGGTG  300
             ||||||||||||||||||||||||||||||||||||||||||||||||||||||||||||
Sbjct  5792  ATAACATAAGCTTCTGACTTCTACCCCCTTCATTCCTTCTTCTCCTGGCTTCTTCAGGTG  5851

Query  301   TCGAAGCTGGTGCCGGTACCGGGTGGACTGTCTACCCTCCCCTAGCTAGCAACCTCGCCC  360
             ||||||||||||||||||||||||||||||||||||||||||||||||||||||||||||
Sbjct  5852  TCGAAGCTGGTGCCGGTACCGGGTGGACTGTCTACCCTCCCCTAGCTAGCAACCTCGCCC  5911

Query  361   ATGCTGGAGCCTCAGTAGATCTAACCATCTTTTCACTGCACCTTGCAGGTATTTCATCAA  420
             ||||||||||||||||||||||||||||||||||||||||||||||||||||||||||||
Sbjct  5912  ATGCTGGAGCCTCAGTAGATCTAACCATCTTTTCACTGCACCTTGCAGGTATTTCATCAA  5971

Query  421   TTCTGGGAGCTATCAACTTCATTACTACCATTATTAACATGAAACCCACAACTGTCACAA  480
             ||||||||||||||||||||||||||||||||||||||||||||||||||||||||||||
Sbjct  5972  TTCTGGGAGCTATCAACTTCATTACTACCATTATTAACATGAAACCCACAACTGTCACAA  6031

Query  481   TATACCAAATTCCCCTGTTTGTCTGAGCCGTCCTAATTACGGCTGTCCTGCTGCTCCTCT  540
             ||||||||||||||||||||||||||||||||||||||||||||||||||||||||||||
Sbjct  6032  TATACCAAATTCCCCTGTTTGTCTGAGCCGTCCTAATTACGGCTGTCCTGCTGCTCCTCT  6091

Query  541   CGCTGCCAGTTTTAGCCGCCGGTATTACAATACTGCTTACAGACCGAAACCTTAATACAA  600
             ||||||||||||||||||||||||||||||||||||||||||||||||||||||||||||
Sbjct  6092  CGCTGCCAGTTTTAGCCGCCGGTATTACAATACTGCTTACAGACCGAAACCTTAATACAA  6151

Query  601   CATTCTTTGACCCTGCAGGAGGAGGGGATCCAATCCTCTACCAACACCTG  650
             ||||||||||||||||||||||||||||||||||||||||||||||||||
Sbjct  6152  CATTCTTTGACCCTGCAGGAGGAGGGGATCCAATCCTCTACCAACACCTG  6201
```

Paralichthys olivaceus voucher XM073 cytochrome oxidase subunit I (COI) gene, partial cds; mitochondrial

Sequence ID: **KX254476.1** Length: 655 Number of Matches: 1

Range 1: 6 to 655

| Score | Expect | Identities | Gaps | Strand | Frame |
|---|---|---|---|---|---|
| 1201 bits(650) | 0.0() | 650/650(100%) | 0/650(0%) | Plus/Plus | |

```
Query  1    TCTATCTCGTATTTGGTGCCTGAGCCGGAATAGTGGGGACAGCCCTAAGCCTCCTCATTC  60
            ||||||||||||||||||||||||||||||||||||||||||||||||||||||||||||
Sbjct  6    TCTATCTCGTATTTGGTGCCTGAGCCGGAATAGTGGGGACAGCCCTAAGCCTCCTCATTC  65

Query  61   GGGCAGAACTCAGCCAACCTGGTGCTCTCCTAGGGGACGACCAGATTTATAACGTAATCG  120
            ||||||||||||||||||||||||||||||||||||||||||||||||||||||||||||
Sbjct  66   GGGCAGAACTCAGCCAACCTGGTGCTCTCCTAGGGGACGACCAGATTTATAACGTAATCG  125

Query  121  TTACCGCACACGCCTTTGTAATAATCTTTTTCATAGTTATACCAATTATGATTGGAGGCT  180
            ||||||||||||||||||||||||||||||||||||||||||||||||||||||||||||
Sbjct  126  TTACCGCACACGCCTTTGTAATAATCTTTTTCATAGTTATACCAATTATGATTGGAGGCT  185

Query  181  TTGGCAACTGACTTATCCCCCTGATAATCGGTGCCCCAGACATAGCATTCCCTCGAATAA  240
            ||||||||||||||||||||||||||||||||||||||||||||||||||||||||||||
Sbjct  186  TTGGCAACTGACTTATCCCCCTGATAATCGGTGCCCCAGACATAGCATTCCCTCGAATAA  245

Query  241  ATAACATAAGCTTCTGACTTCTACCCCCTTCATTCCTTCTTCTCCTGGCTTCTTCAGGTG  300
            ||||||||||||||||||||||||||||||||||||||||||||||||||||||||||||
Sbjct  246  ATAACATAAGCTTCTGACTTCTACCCCCTTCATTCCTTCTTCTCCTGGCTTCTTCAGGTG  305

Query  301  TCGAAGCTGGTGCCGGTACCGGGTGGACTGTCTACCCTCCCCTAGCTAGCAACCTCGCCC  360
            ||||||||||||||||||||||||||||||||||||||||||||||||||||||||||||
Sbjct  306  TCGAAGCTGGTGCCGGTACCGGGTGGACTGTCTACCCTCCCCTAGCTAGCAACCTCGCCC  365

Query  361  ATGCTGGAGCCTCAGTAGATCTAACCATCTTTTCACTGCACCTTGCAGGTATTTCATCAA  420
            ||||||||||||||||||||||||||||||||||||||||||||||||||||||||||||
Sbjct  366  ATGCTGGAGCCTCAGTAGATCTAACCATCTTTTCACTGCACCTTGCAGGTATTTCATCAA  425

Query  421  TTCTGGGAGCTATCAACTTCATTACTACCATTATTAACATGAAACCCACAACTGTCACAA  480
            ||||||||||||||||||||||||||||||||||||||||||||||||||||||||||||
Sbjct  426  TTCTGGGAGCTATCAACTTCATTACTACCATTATTAACATGAAACCCACAACTGTCACAA  485

Query  481  TATACCAAATTCCCCTGTTTGTCTGAGCCGTCCTAATTACGGCTGTCCTGCTGCTCCTCT  540
            ||||||||||||||||||||||||||||||||||||||||||||||||||||||||||||
Sbjct  486  TATACCAAATTCCCCTGTTTGTCTGAGCCGTCCTAATTACGGCTGTCCTGCTGCTCCTCT  545

Query  541  CGCTGCCAGTTTTAGCCGCCGGTATTACAATACTGCTTACAGACCGAAACCTTAATACAA  600
            ||||||||||||||||||||||||||||||||||||||||||||||||||||||||||||
Sbjct  546  CGCTGCCAGTTTTAGCCGCCGGTATTACAATACTGCTTACAGACCGAAACCTTAATACAA  605

Query  601  CATTCTTTGACCCTGCAGGAGGAGGGGATCCAATCCTCTACCAACACCTG  650
            ||||||||||||||||||||||||||||||||||||||||||||||||||
Sbjct  606  CATTCTTTGACCCTGCAGGAGGAGGGGATCCAATCCTCTACCAACACCTG  655
```

Paralichthys olivaceus voucher HME1 cytochrome oxidase subunit 1 (COI) gene, partial cds; mitochondrial

Sequence ID: **JF952803.1** Length: 652 Number of Matches: 1

Range 1: 3 to 652

| Score | Expect | Identities | Gaps | Strand | Frame |
|---|---|---|---|---|---|
| 1201 bits(650) | 0.0() | 650/650(100%) | 0/650(0%) | Plus/Plus | |

```
Query  1    TCTATCTCGTATTTGGTGCCTGAGCCGGAATAGTGGGGACAGCCCTAAGCCTCCTCATTC  60
            ||||||||||||||||||||||||||||||||||||||||||||||||||||||||||||
Sbjct  3    TCTATCTCGTATTTGGTGCCTGAGCCGGAATAGTGGGGACAGCCCTAAGCCTCCTCATTC  62

Query  61   GGGCAGAACTCAGCCAACCTGGTGCTCTCCTAGGGGACGACCAGATTTATAACGTAATCG  120
            ||||||||||||||||||||||||||||||||||||||||||||||||||||||||||||
Sbjct  63   GGGCAGAACTCAGCCAACCTGGTGCTCTCCTAGGGGACGACCAGATTTATAACGTAATCG  122

Query  121  TTACCGCACACGCCTTTGTAATAATCTTTTTCATAGTTATACCAATTATGATTGGAGGCT  180
            ||||||||||||||||||||||||||||||||||||||||||||||||||||||||||||
Sbjct  123  TTACCGCACACGCCTTTGTAATAATCTTTTTCATAGTTATACCAATTATGATTGGAGGCT  182

Query  181  TTGGCAACTGACTTATCCCCCTGATAATCGGTGCCCCAGACATAGCATTCCCTCGAATAA  240
            ||||||||||||||||||||||||||||||||||||||||||||||||||||||||||||
Sbjct  183  TTGGCAACTGACTTATCCCCCTGATAATCGGTGCCCCAGACATAGCATTCCCTCGAATAA  242

Query  241  ATAACATAAGCTTCTGACTTCTACCCCCTTCATTCCTTCTTCTCCTGGCTTCTTCAGGTG  300
            ||||||||||||||||||||||||||||||||||||||||||||||||||||||||||||
Sbjct  243  ATAACATAAGCTTCTGACTTCTACCCCCTTCATTCCTTCTTCTCCTGGCTTCTTCAGGTG  302

Query  301  TCGAAGCTGGTGCCGGTACCGGGTGGACTGTCTACCCTCCCCTAGCTAGCAACCTCGCCC  360
            ||||||||||||||||||||||||||||||||||||||||||||||||||||||||||||
Sbjct  303  TCGAAGCTGGTGCCGGTACCGGGTGGACTGTCTACCCTCCCCTAGCTAGCAACCTCGCCC  362

Query  361  ATGCTGGAGCCTCAGTAGATCTAACCATCTTTTCACTGCACCTTGCAGGTATTTCATCAA  420
            ||||||||||||||||||||||||||||||||||||||||||||||||||||||||||||
Sbjct  363  ATGCTGGAGCCTCAGTAGATCTAACCATCTTTTCACTGCACCTTGCAGGTATTTCATCAA  422

Query  421  TTCTGGGAGCTATCAACTTCATTACTACCATTATTAACATGAAACCCACAACTGTCACAA  480
            ||||||||||||||||||||||||||||||||||||||||||||||||||||||||||||
Sbjct  423  TTCTGGGAGCTATCAACTTCATTACTACCATTATTAACATGAAACCCACAACTGTCACAA  482

Query  481  TATACCAAATTCCCCTGTTTGTCTGAGCCGTCCTAATTACGGCTGTCCTGCTGCTCCTCT  540
            ||||||||||||||||||||||||||||||||||||||||||||||||||||||||||||
Sbjct  483  TATACCAAATTCCCCTGTTTGTCTGAGCCGTCCTAATTACGGCTGTCCTGCTGCTCCTCT  542

Query  541  CGCTGCCAGTTTTAGCCGCCGGTATTACAATACTGCTTACAGACCGAAACCTTAATACAA  600
            ||||||||||||||||||||||||||||||||||||||||||||||||||||||||||||
Sbjct  543  CGCTGCCAGTTTTAGCCGCCGGTATTACAATACTGCTTACAGACCGAAACCTTAATACAA  602

Query  601  CATTCTTTGACCCTGCAGGAGGAGGGGATCCAATCCTCTACCAACACCTG  650
            ||||||||||||||||||||||||||||||||||||||||||||||||||
Sbjct  603  CATTCTTTGACCCTGCAGGAGGAGGGGATCCAATCCTCTACCAACACCTG  652
```

Paralichthys olivaceus isolate CO1B7 cytochrome oxidase subunit I-like (COI) gene, partial sequence; mitochondrial
Sequence ID: **EU266369.1** Length: 713 Number of Matches: 1
Range 1: 31 to 680

| Score | Expect | Identities | Gaps | Strand | Frame |
|---|---|---|---|---|---|
| 1201 bits(650) | 0.0() | 650/650(100%) | 0/650(0%) | Plus/Plus | |

```
Query  1    TCTATCTCGTATTTGGTGCCTGAGCCGGAATAGTGGGGACAGCCCTAAGCCTCCTCATTC  60
            ||||||||||||||||||||||||||||||||||||||||||||||||||||||||||||
Sbjct  31   TCTATCTCGTATTTGGTGCCTGAGCCGGAATAGTGGGGACAGCCCTAAGCCTCCTCATTC  90

Query  61   GGGCAGAACTCAGCCAACCTGGTGCTCTCCTAGGGGACGACCAGATTTATAACGTAATCG  120
            ||||||||||||||||||||||||||||||||||||||||||||||||||||||||||||
Sbjct  91   GGGCAGAACTCAGCCAACCTGGTGCTCTCCTAGGGGACGACCAGATTTATAACGTAATCG  150

Query  121  TTACCGCACACGCCTTTGTAATAATCTTTTTCATAGTTATACCAATTATGATTGGAGGCT  180
            ||||||||||||||||||||||||||||||||||||||||||||||||||||||||||||
Sbjct  151  TTACCGCACACGCCTTTGTAATAATCTTTTTCATAGTTATACCAATTATGATTGGAGGCT  210

Query  181  TTGGCAACTGACTTATCCCCCTGATAATCGGTGCCCCAGACATAGCATTCCCTCGAATAA  240
            ||||||||||||||||||||||||||||||||||||||||||||||||||||||||||||
Sbjct  211  TTGGCAACTGACTTATCCCCCTGATAATCGGTGCCCCAGACATAGCATTCCCTCGAATAA  270

Query  241  ATAACATAAGCTTCTGACTTCTACCCCCTTCATTCCTTCTTCTCCTGGCTTCTTCAGGTG  300
            ||||||||||||||||||||||||||||||||||||||||||||||||||||||||||||
Sbjct  271  ATAACATAAGCTTCTGACTTCTACCCCCTTCATTCCTTCTTCTCCTGGCTTCTTCAGGTG  330

Query  301  TCGAAGCTGGTGCCGGTACCGGGTGGACTGTCTACCCTCCCCTAGCTAGCAACCTCGCCC  360
            ||||||||||||||||||||||||||||||||||||||||||||||||||||||||||||
Sbjct  331  TCGAAGCTGGTGCCGGTACCGGGTGGACTGTCTACCCTCCCCTAGCTAGCAACCTCGCCC  390

Query  361  ATGCTGGAGCCTCAGTAGATCTAACCATCTTTTCACTGCACCTTGCAGGTATTTCATCAA  420
            ||||||||||||||||||||||||||||||||||||||||||||||||||||||||||||
Sbjct  391  ATGCTGGAGCCTCAGTAGATCTAACCATCTTTTCACTGCACCTTGCAGGTATTTCATCAA  450

Query  421  TTCTGGGAGCTATCAACTTCATTACTACCATTATTAACATGAAACCCACAACTGTCACAA  480
            ||||||||||||||||||||||||||||||||||||||||||||||||||||||||||||
Sbjct  451  TTCTGGGAGCTATCAACTTCATTACTACCATTATTAACATGAAACCCACAACTGTCACAA  510

Query  481  TATACCAAATTCCCCTGTTTGTCTGAGCCGTCCTAATTACGGCTGTCCTGCTGCTCCTCT  540
            ||||||||||||||||||||||||||||||||||||||||||||||||||||||||||||
Sbjct  511  TATACCAAATTCCCCTGTTTGTCTGAGCCGTCCTAATTACGGCTGTCCTGCTGCTCCTCT  570

Query  541  CGCTGCCAGTTTTAGCCGCCGGTATTACAATACTGCTTACAGACCGAAACCTTAATACAA  600
            ||||||||||||||||||||||||||||||||||||||||||||||||||||||||||||
Sbjct  571  CGCTGCCAGTTTTAGCCGCCGGTATTACAATACTGCTTACAGACCGAAACCTTAATACAA  630

Query  601  CATTCTTTGACCCTGCAGGAGGAGGGGATCCAATCCTCTACCAACACCTG  650
```

```
                ||||||||||||||||||||||||||||||||||||||||||||||||||
Sbjct  631   CATTCTTTGACCCTGCAGGAGGAGGGGATCCAATCCTCTACCAACACCTG  680
```

Paralichthys olivaceus voucher HUM I-00297 cytochrome c oxidase subunit 1 (COI) gene, complete cds; mitochondrial

Sequence ID: **MH032483.1** Length: 1551 Number of Matches: 1

Range 1: 53 to 702

| Score | Expect | Identities | Gaps | Strand | Frame |
|---|---|---|---|---|---|
| 1195 bits(647) | 0.0() | 649/650(99%) | 0/650(0%) | Plus/Plus | |

```
Query  1     TCTATCTCGTATTTGGTGCCTGAGCCGGAATAGTGGGGACAGCCCTAAGCCTCCTCATTC  60
             ||||||||||||||||||||||||||||||||||||||||||||||||||||||||||||
Sbjct  53    TCTATCTCGTATTTGGTGCCTGAGCCGGAATAGTGGGGACAGCCCTAAGCCTCCTCATTC  112

Query  61    GGGCAGAACTCAGCCAACCTGGTGCTCTCCTAGGGGACGACCAGATTTATAACGTAATCG  120
             ||||||||||||||||||||||||||||||||||||||||||||||||||||||||||||
Sbjct  113   GGGCAGAACTCAGCCAACCTGGTGCTCTCCTAGGGGACGACCAGATTTATAACGTAATCG  172

Query  121   TTACCGCACACGCCTTTGTAATAATCTTTTTCATAGTTATACCAATTATGATTGGAGGCT  180
             ||||||||||||||||||||||||||||||||||||||||||||||||||||||||||||
Sbjct  173   TTACCGCACACGCCTTTGTAATAATCTTTTTCATAGTTATACCAATTATGATTGGAGGCT  232

Query  181   TTGGCAACTGACTTATCCCCCTGATAATCGGTGCCCCAGACATAGCATTCCCTCGAATAA  240
             ||||||||||||||||||||||||||||||||||||||||||||||||||||||||||||
Sbjct  233   TTGGCAACTGACTTATCCCCCTGATAATCGGTGCCCCAGACATAGCATTCCCTCGAATAA  292

Query  241   ATAACATAAGCTTCTGACTTCTACCCCCTTCATTCCTTCTTCTCCTGGCTTCTTCAGGTG  300
             ||||||||||||||||||||||||||||||||||||||||||||||||||||||||||||
Sbjct  293   ATAACATAAGCTTCTGACTTCTACCCCCTTCATTCCTTCTTCTCCTGGCTTCTTCAGGTG  352

Query  301   TCGAAGCTGGTGCCGGTACCGGGTGGACTGTCTACCCTCCCCTAGCTAGCAACCTCGCCC  360
             ||||||||||||||||||||||||||||||||||||||||||||||||||||||||||||
Sbjct  353   TCGAAGCTGGTGCCGGTACCGGGTGGACTGTCTACCCTCCCCTAGCTAGCAACCTCGCCC  412

Query  361   ATGCTGGAGCCTCAGTAGATCTAACCATCTTTTCACTGCACCTTGCAGGTATTTCATCAA  420
             ||||||||||||||||||||||||||||||||||||||||||||||||||||||||||||
Sbjct  413   ATGCTGGAGCCTCAGTAGATCTAACCATCTTTTCACTGCACCTTGCAGGTATTTCATCAA  472

Query  421   TTCTGGGAGCTATCAACTTCATTACTACCATTATTAACATGAAACCCACAACTGTCACAA  480
             ||||||||||||||||||| ||||||||||||||||||||||||||||||||||||||||
Sbjct  473   TTCTGGGAGCTATCAACTTTATTACTACCATTATTAACATGAAACCCACAACTGTCACAA  532

Query  481   TATACCAAATTCCCCTGTTTGTCTGAGCCGTCCTAATTACGGCTGTCCTGCTGCTCCTCT  540
             ||||||||||||||||||||||||||||||||||||||||||||||||||||||||||||
Sbjct  533   TATACCAAATTCCCCTGTTTGTCTGAGCCGTCCTAATTACGGCTGTCCTGCTGCTCCTCT  592

Query  541   CGCTGCCAGTTTTAGCCGCCGGTATTACAATACTGCTTACAGACCGAAACCTTAATACAA  600
             ||||||||||||||||||||||||||||||||||||||||||||||||||||||||||||
Sbjct  593   CGCTGCCAGTTTTAGCCGCCGGTATTACAATACTGCTTACAGACCGAAACCTTAATACAA  652

Query  601   CATTCTTTGACCCTGCAGGAGGAGGGGATCCAATCCTCTACCAACACCTG  650
             ||||||||||||||||||||||||||||||||||||||||||||||||||
Sbjct  653   CATTCTTTGACCCTGCAGGAGGAGGGGATCCAATCCTCTACCAACACCTG  702
```

Paralichthys olivaceus voucher XM072 cytochrome oxidase subunit I (COI) gene, partial cds; mitochondrial

Sequence ID: **KX254475.1** Length: 655 Number of Matches: 1

Range 1: 6 to 655

| Score | Expect | Identities | Gaps | Strand | Frame |
|---|---|---|---|---|---|
| 1195 bits(647) | 0.0() | 649/650(99%) | 0/650(0%) | Plus/Plus | |

```
Query  1     TCTATCTCGTATTTGGTGCCTGAGCCGGAATAGTGGGGACAGCCCTAAGCCTCCTCATTC  60
             ||||||||||||||||||||||||||||||||||||||||||||||||||||||||||||
Sbjct  6     TCTATCTCGTATTTGGTGCCTGAGCCGGAATAGTGGGGACAGCCCTAAGCCTCCTCATTC  65

Query  61    GGGCAGAACTCAGCCAACCTGGTGCTCTCCTAGGGGACGACCAGATTTATAACGTAATCG  120
             ||||||||||||||||||||||||||||||||||||||||||||||||||||||||||||
Sbjct  66    GGGCAGAACTCAGCCAACCTGGTGCTCTCCTAGGGGACGACCAGATTTATAACGTAATCG  125

Query  121   TTACCGCACACGCCTTTGTAATAATCTTTTTCATAGTTATACCAATTATGATTGGAGGCT  180
             ||||||||||||||||||||||||||||||||||||||||||||||||||||||||||||
Sbjct  126   TTACCGCACACGCCTTTGTAATAATCTTTTTCATAGTTATACCAATTATGATTGGAGGCT  185

Query  181   TTGGCAACTGACTTATCCCCCTGATAATCGGTGCCCCAGACATAGCATTCCCTCGAATAA  240
             |||||||||||||||||||||| |||||||||||||||||||||||||||||||||||||
Sbjct  186   TTGGCAACTGACTTATCCCCCTAATAATCGGTGCCCCAGACATAGCATTCCCTCGAATAA  245

Query  241   ATAACATAAGCTTCTGACTTCTACCCCCTTCATTCCTTCTTCTCCTGGCTTCTTCAGGTG  300
             ||||||||||||||||||||||||||||||||||||||||||||||||||||||||||||
Sbjct  246   ATAACATAAGCTTCTGACTTCTACCCCCTTCATTCCTTCTTCTCCTGGCTTCTTCAGGTG  305

Query  301   TCGAAGCTGGTGCCGGTACCGGGTGGACTGTCTACCCTCCCCTAGCTAGCAACCTCGCCC  360
             ||||||||||||||||||||||||||||||||||||||||||||||||||||||||||||
Sbjct  306   TCGAAGCTGGTGCCGGTACCGGGTGGACTGTCTACCCTCCCCTAGCTAGCAACCTCGCCC  365

Query  361   ATGCTGGAGCCTCAGTAGATCTAACCATCTTTTCACTGCACCTTGCAGGTATTTCATCAA  420
             ||||||||||||||||||||||||||||||||||||||||||||||||||||||||||||
Sbjct  366   ATGCTGGAGCCTCAGTAGATCTAACCATCTTTTCACTGCACCTTGCAGGTATTTCATCAA  425

Query  421   TTCTGGGAGCTATCAACTTCATTACTACCATTATTAACATGAAACCCACAACTGTCACAA  480
             ||||||||||||||||||||||||||||||||||||||||||||||||||||||||||||
```

```
Sbjct  426   TTCTGGGAGCTATCAACTTCATTACTACCATTATTAACATGAAACCCACAACTGTCACAA  485

Query  481   TATACCAAATTCCCCTGTTTGTCTGAGCCGTCCTAATTACGGCTGTCCTGCTGCTCCTCT  540
             ||||||||||||||||||||||||||||||||||||||||||||||||||||||||||||
Sbjct  486   TATACCAAATTCCCCTGTTTGTCTGAGCCGTCCTAATTACGGCTGTCCTGCTGCTCCTCT  545

Query  541   CGCTGCCAGTTTTAGCCGCCGGTATTACAATACTGCTTACAGACCGAAACCTTAATACAA  600
             ||||||||||||||||||||||||||||||||||||||||||||||||||||||||||||
Sbjct  546   CGCTGCCAGTTTTAGCCGCCGGTATTACAATACTGCTTACAGACCGAAACCTTAATACAA  605

Query  601   CATTCTTTGACCCTGCAGGAGGAGGGGATCCAATCCTCTACCAACACCTG  650
             ||||||||||||||||||||||||||||||||||||||||||||||||||
Sbjct  606   CATTCTTTGACCCTGCAGGAGGAGGGGATCCAATCCTCTACCAACACCTG  655
```

Paralichthys olivaceus voucher HUM I-00296 cytochrome c oxidase subunit 1 (COI) gene, complete cds; mitochondrial

Sequence ID: **MH032482.1** Length: 1551 Number of Matches: 1

Range 1: 53 to 702

| Score | Expect | Identities | Gaps | Strand | Frame |
|---|---|---|---|---|---|
| 1190 bits(644) | 0.0() | 648/650(99%) | 0/650(0%) | Plus/Plus | |

```
Query  1     TCTATCTCGTATTTGGTGCCTGAGCCGGAATAGTGGGGACAGCCCTAAGCCTCCTCATTC  60
             ||||||||||||||||||||||||||||||||||||||||||||||||||||||||||||
Sbjct  53    TCTATCTCGTATTTGGTGCCTGAGCCGGAATAGTGGGGACAGCCCTAAGCCTCCTCATTC  112

Query  61    GGGCAGAACTCAGCCAACCTGGTGCTCTCCTAGGGGACGACCAGATTTATAACGTAATCG  120
             ||||||||||||||||||||||||||||||||||||||||||||||||||||||||||||
Sbjct  113   GGGCAGAACTCAGCCAACCTGGTGCTCTCCTAGGGGACGACCAGATTTATAACGTAATCG  172

Query  121   TTACCGCACACGCCTTTGTAATAATCTTTTTCATAGTTATACCAATTATGATTGGAGGCT  180
             ||||||||||||||||||||||||||||||||||||||||||||||||||||||||||||
Sbjct  173   TTACCGCACACGCCTTTGTAATAATCTTTTTCATAGTTATACCAATTATGATTGGAGGCT  232

Query  181   TTGGCAACTGACTTATCCCCCTGATAATCGGTGCCCCAGACATAGCATTCCCTCGAATAA  240
             ||||||||||||||||||||||||||||||||||||||||||||||||||||||||||||
Sbjct  233   TTGGCAACTGACTTATCCCCCTGATAATCGGTGCCCCAGACATAGCATTCCCTCGAATAA  292

Query  241   ATAACATAAGCTTCTGACTTCTACCCCCTTCATTCCTTCTTCTCCTGGCTTCTTCAGGTG  300
             |||| |||||||||||||||||||||||||||||||||||||||||||||||||||||||
Sbjct  293   ATAATATAAGCTTCTGACTTCTACCCCCTTCATTCCTTCTTCTCCTGGCTTCTTCAGGTG  352

Query  301   TCGAAGCTGGTGCCGGTACCGGGTGGACTGTCTACCCTCCCCTAGCTAGCAACCTCGCCC  360
             ||||||||||||||||||||||||||||||||||||||||||||||||||||||||||||
Sbjct  353   TCGAAGCTGGTGCCGGTACCGGGTGGACTGTCTACCCTCCCCTAGCTAGCAACCTCGCCC  412

Query  361   ATGCTGGAGCCTCAGTAGATCTAACCATCTTTTCACTGCACCTTGCAGGTATTTCATCAA  420
             ||||||||||||||||||||||||||||||||||||| ||||||||||||||||||||||
Sbjct  413   ATGCTGGAGCCTCAGTAGATCTAACCATCTTTTCACTACACCTTGCAGGTATTTCATCAA  472

Query  421   TTCTGGGAGCTATCAACTTCATTACTACCATTATTAACATGAAACCCACAACTGTCACAA  480
             ||||||||||||||||||||||||||||||||||||||||||||||||||||||||||||
Sbjct  473   TTCTGGGAGCTATCAACTTCATTACTACCATTATTAACATGAAACCCACAACTGTCACAA  532

Query  481   TATACCAAATTCCCCTGTTTGTCTGAGCCGTCCTAATTACGGCTGTCCTGCTGCTCCTCT  540
             ||||||||||||||||||||||||||||||||||||||||||||||||||||||||||||
Sbjct  533   TATACCAAATTCCCCTGTTTGTCTGAGCCGTCCTAATTACGGCTGTCCTGCTGCTCCTCT  592

Query  541   CGCTGCCAGTTTTAGCCGCCGGTATTACAATACTGCTTACAGACCGAAACCTTAATACAA  600
             ||||||||||||||||||||||||||||||||||||||||||||||||||||||||||||
Sbjct  593   CGCTGCCAGTTTTAGCCGCCGGTATTACAATACTGCTTACAGACCGAAACCTTAATACAA  652

Query  601   CATTCTTTGACCCTGCAGGAGGAGGGGATCCAATCCTCTACCAACACCTG  650
             ||||||||||||||||||||||||||||||||||||||||||||||||||
Sbjct  653   CATTCTTTGACCCTGCAGGAGGAGGGGATCCAATCCTCTACCAACACCTG  702
```

Paralichthys olivaceus voucher XM074 cytochrome oxidase subunit I (COI) gene, partial cds; mitochondrial

Sequence ID: **KX254477.1** Length: 655 Number of Matches: 1

Range 1: 6 to 655

| Score | Expect | Identities | Gaps | Strand | Frame |
|---|---|---|---|---|---|
| 1190 bits(644) | 0.0() | 648/650(99%) | 0/650(0%) | Plus/Plus | |

```
Query  1     TCTATCTCGTATTTGGTGCCTGAGCCGGAATAGTGGGGACAGCCCTAAGCCTCCTCATTC  60
             ||||||||||||||||||||||||||||||||||||||||||||||||||||||||||||
Sbjct  6     TCTATCTCGTATTTGGTGCCTGAGCCGGAATAGTGGGGACAGCCCTAAGCCTCCTCATTC  65

Query  61    GGGCAGAACTCAGCCAACCTGGTGCTCTCCTAGGGGACGACCAGATTTATAACGTAATCG  120
             ||||||||||||||||||||||||||||||||||||||||||||||||||||||||||||
Sbjct  66    GGGCAGAACTCAGCCAACCTGGTGCTCTCCTAGGGGACGACCAGATTTATAACGTAATCG  125

Query  121   TTACCGCACACGCCTTTGTAATAATCTTTTTCATAGTTATACCAATTATGATTGGAGGCT  180
             ||||||||||||||||||||||||||||||||||||||||||||||||||||||||||||
Sbjct  126   TTACCGCACACGCCTTTGTAATAATCTTTTTCATAGTTATACCAATTATGATTGGAGGCT  185

Query  181   TTGGCAACTGACTTATCCCCCTGATAATCGGTGCCCCAGACATAGCATTCCCTCGAATAA  240
             |||||||||||||||||||||| |||||||||||||||||||||||||||||||||||||
Sbjct  186   TTGGCAACTGACTTATCCCCCTAATAATCGGTGCCCCAGACATAGCATTCCCTCGAATAA  245

Query  241   ATAACATAAGCTTCTGACTTCTACCCCCTTCATTCCTTCTTCTCCTGGCTTCTTCAGGTG  300
             ||||||||||||||||||||||||||||||||||||||||||||||||||||||||||||
Sbjct  246   ATAACATAAGCTTCTGACTTCTACCCCCTTCATTCCTTCTTCTCCTGGCTTCTTCAGGTG  305
```

```
Query  301   TCGAAGCTGGTGCCGGTACCGGGTGGACTGTCTACCCTCCCCTAGCTAGCAACCTCGCCC  360
             ||||||||||||||||||||||||||||||||||||||||||||||||||||||||||||
Sbjct  306   TCGAAGCTGGTGCCGGTACCGGGTGGACTGTCTACCCTCCCCTAGCTAGCAACCTCGCCC  365

Query  361   ATGCTGGAGCCTCAGTAGATCTAACCATCTTTTCACTGCACCTTGCAGGTATTTCATCAA  420
             ||||||||||||||||||||||||||||||||||||| ||||||||||||||||||||||
Sbjct  366   ATGCTGGAGCCTCAGTAGATCTAACCATCTTTTCACTACACCTTGCAGGTATTTCATCAA  425

Query  421   TTCTGGGAGCTATCAACTTCATTACTACCATTATTAACATGAAACCCACAACTGTCACAA  480
             ||||||||||||||||||||||||||||||||||||||||||||||||||||||||||||
Sbjct  426   TTCTGGGAGCTATCAACTTCATTACTACCATTATTAACATGAAACCCACAACTGTCACAA  485

Query  481   TATACCAAATTCCCCTGTTTGTCTGAGCCGTCCTAATTACGGCTGTCCTGCTGCTCCTCT  540
             ||||||||||||||||||||||||||||||||||||||||||||||||||||||||||||
Sbjct  486   TATACCAAATTCCCCTGTTTGTCTGAGCCGTCCTAATTACGGCTGTCCTGCTGCTCCTCT  545

Query  541   CGCTGCCAGTTTTAGCCGCCGGTATTACAATACTGCTTACAGACCGAAACCTTAATACAA  600
             ||||||||||||||||||||||||||||||||||||||||||||||||||||||||||||
Sbjct  546   CGCTGCCAGTTTTAGCCGCCGGTATTACAATACTGCTTACAGACCGAAACCTTAATACAA  605

Query  601   CATTCTTTGACCCTGCAGGAGGAGGGGATCCAATCCTCTACCAACACCTG  650
             ||||||||||||||||||||||||||||||||||||||||||||||||||
Sbjct  606   CATTCTTTGACCCTGCAGGAGGAGGGGATCCAATCCTCTACCAACACCTG  655
```

Paralichthys olivaceus voucher HME2 cytochrome oxidase subunit 1 (COI) gene, partial cds; mitochondrial

Sequence ID: **JF952804.1** Length: 652 Number of Matches: 1

Range 1: 3 to 652

| Score | Expect | Identities | Gaps | Strand | Frame |
|---|---|---|---|---|---|
| 1190 bits(644) | 0.0() | 648/650(99%) | 0/650(0%) | Plus/Plus | |

```
Query  1     TCTATCTCGTATTTGGTGCCTGAGCCGGAATAGTGGGGACAGCCCTAAGCCTCCTCATTC  60
             ||||||||||||||||||||||||||||||||||||||||||||||||||||||||||||
Sbjct  3     TCTATCTCGTATTTGGTGCCTGAGCCGGAATAGTGGGGACAGCCCTAAGCCTCCTCATTC  62

Query  61    GGGCAGAACTCAGCCAACCTGGTGCTCTCCTAGGGGACGACCAGATTTATAACGTAATCG  120
             ||||||||||||||||||||||||||||||||||||||||||||||||||||||||||||
Sbjct  63    GGGCAGAACTCAGCCAACCTGGTGCTCTCCTAGGGGACGACCAGATTTATAACGTAATCG  122

Query  121   TTACCGCACACGCCTTTGTAATAATCTTTTTCATAGTTATACCAATTATGATTGGAGGCT  180
             ||||||||||||||||||||||||||||||||||||||||||||||||||||||||||||
Sbjct  123   TTACCGCACACGCCTTTGTAATAATCTTTTTCATAGTTATACCAATTATGATTGGAGGCT  182

Query  181   TTGGCAACTGACTTATCCCCCTGATAATCGGTGCCCCAGACATAGCATTCCCTCGAATAA  240
             ||||||||||||||||||||||||||||||||||||||||||||||||||||||||||||
Sbjct  183   TTGGCAACTGACTTATCCCCCTGATAATCGGTGCCCCAGACATAGCATTCCCTCGAATAA  242

Query  241   ATAACATAAGCTTCTGACTTCTACCCCCTTCATTCCTTCTTCTCCTGGCTTCTTCAGGTG  300
             |||| |||||||||||||||||||||||||||||||||||||||||||||||||||||||
Sbjct  243   ATAATATAAGCTTCTGACTTCTACCCCCTTCATTCCTTCTTCTCCTGGCTTCTTCAGGTG  302

Query  301   TCGAAGCTGGTGCCGGTACCGGGTGGACTGTCTACCCTCCCCTAGCTAGCAACCTCGCCC  360
             ||||||||||||||||||||||||||||||||||||||||||||||||||||||||||||
Sbjct  303   TCGAAGCTGGTGCCGGTACCGGGTGGACTGTCTACCCTCCCCTAGCTAGCAACCTCGCCC  362

Query  361   ATGCTGGAGCCTCAGTAGATCTAACCATCTTTTCACTGCACCTTGCAGGTATTTCATCAA  420
             ||||||||||||||||||||||||||||||||||||| ||||||||||||||||||||||
Sbjct  363   ATGCTGGAGCCTCAGTAGATCTAACCATCTTTTCACTACACCTTGCAGGTATTTCATCAA  422

Query  421   TTCTGGGAGCTATCAACTTCATTACTACCATTATTAACATGAAACCCACAACTGTCACAA  480
             ||||||||||||||||||||||||||||||||||||||||||||||||||||||||||||
Sbjct  423   TTCTGGGAGCTATCAACTTCATTACTACCATTATTAACATGAAACCCACAACTGTCACAA  482

Query  481   TATACCAAATTCCCCTGTTTGTCTGAGCCGTCCTAATTACGGCTGTCCTGCTGCTCCTCT  540
             ||||||||||||||||||||||||||||||||||||||||||||||||||||||||||||
Sbjct  483   TATACCAAATTCCCCTGTTTGTCTGAGCCGTCCTAATTACGGCTGTCCTGCTGCTCCTCT  542

Query  541   CGCTGCCAGTTTTAGCCGCCGGTATTACAATACTGCTTACAGACCGAAACCTTAATACAA  600
             ||||||||||||||||||||||||||||||||||||||||||||||||||||||||||||
Sbjct  543   CGCTGCCAGTTTTAGCCGCCGGTATTACAATACTGCTTACAGACCGAAACCTTAATACAA  602

Query  601   CATTCTTTGACCCTGCAGGAGGAGGGGATCCAATCCTCTACCAACACCTG  650
             ||||||||||||||||||||||||||||||||||||||||||||||||||
Sbjct  603   CATTCTTTGACCCTGCAGGAGGAGGGGATCCAATCCTCTACCAACACCTG  652
```

Paralichthys olivaceus isolate CO1B8 cytochrome oxidase subunit I (COI) gene, partial cds; mitochondrial

Sequence ID: **EU266368.1** Length: 710 Number of Matches: 1

Range 1: 30 to 679

| Score | Expect | Identities | Gaps | Strand | Frame |
|---|---|---|---|---|---|
| 1190 bits(644) | 0.0() | 648/650(99%) | 0/650(0%) | Plus/Plus | |

```
Query  1     TCTATCTCGTATTTGGTGCCTGAGCCGGAATAGTGGGGACAGCCCTAAGCCTCCTCATTC  60
             ||||||||||||||||||||||||||||||||||||||||||||||||||||||||||||
Sbjct  30    TCTATCTCGTATTTGGTGCCTGAGCCGGAATAGTGGGGACAGCCCTAAGCCTCCTCATTC  89

Query  61    GGGCAGAACTCAGCCAACCTGGTGCTCTCCTAGGGGACGACCAGATTTATAACGTAATCG  120
             ||||||||||||||||||||||||||||||||||||||||||||||||||||||||||||
Sbjct  90    GGGCAGAACTCAGCCAACCTGGTGCTCTCCTAGGGGACGACCAGATTTATAACGTAATCG  149
```

```
Query  121  TTACCGCACACGCCTTTGTAATAATCTTTTTCATAGTTATACCAATTATGATTGGAGGCT  180
            ||||||||||||||||||||||||||||||||||||||||||||||||||||||||||||
Sbjct  150  TTACCGCACACGCCTTTGTAATAATCTTTTTCATAGTTATACCAATTATGATTGGAGGCT  209

Query  181  TTGGCAACTGACTTATCCCCCTGATAATCGGTGCCCCAGACATAGCATTCCCTCGAATAA  240
            ||||||||||||||||||||||||||||||||||||||||||||||||||||||||||||
Sbjct  210  TTGGCAACTGACTTATCCCCCTGATAATCGGTGCCCCAGACATAGCATTCCCTCGAATAA  269

Query  241  ATAACATAAGCTTCTGACTTCTACCCCCTTCATTCCTTCTTCTCCTGGCTTCTTCAGGTG  300
            |||| |||||||||||||||||||||||||||||||||||||||||||||||||||||||
Sbjct  270  ATAATATAAGCTTCTGACTTCTACCCCCTTCATTCCTTCTTCTCCTGGCTTCTTCAGGTG  329

Query  301  TCGAAGCTGGTGCCGGTACCGGGTGGACTGTCTACCCTCCCCTAGCTAGCAACCTCGCCC  360
            ||||||||||||||||||||||||||||||||||||||||||||||||||||||||||||
Sbjct  330  TCGAAGCTGGTGCCGGTACCGGGTGGACTGTCTACCCTCCCCTAGCTAGCAACCTCGCCC  389

Query  361  ATGCTGGAGCCTCAGTAGATCTAACCATCTTTTCACTGCACCTTGCAGGTATTTCATCAA  420
            |||||||||||||||||||||||||||||||||||||| |||||||||||||||||||||
Sbjct  390  ATGCTGGAGCCTCAGTAGATCTAACCATCTTTTCACTACACCTTGCAGGTATTTCATCAA  449

Query  421  TTCTGGGAGCTATCAACTTCATTACTACCATTATTAACATGAAACCCACAACTGTCACAA  480
            ||||||||||||||||||||||||||||||||||||||||||||||||||||||||||||
Sbjct  450  TTCTGGGAGCTATCAACTTCATTACTACCATTATTAACATGAAACCCACAACTGTCACAA  509

Query  481  TATACCAAATTCCCCTGTTTGTCTGAGCCGTCCTAATTACGGCTGTCCTGCTGCTCCTCT  540
            ||||||||||||||||||||||||||||||||||||||||||||||||||||||||||||
Sbjct  510  TATACCAAATTCCCCTGTTTGTCTGAGCCGTCCTAATTACGGCTGTCCTGCTGCTCCTCT  569

Query  541  CGCTGCCAGTTTTAGCCGCCGGTATTACAATACTGCTTACAGACCGAAACCTTAATACAA  600
            ||||||||||||||||||||||||||||||||||||||||||||||||||||||||||||
Sbjct  570  CGCTGCCAGTTTTAGCCGCCGGTATTACAATACTGCTTACAGACCGAAACCTTAATACAA  629

Query  601  CATTCTTTGACCCTGCAGGAGGAGGGGATCCAATCCTCTACCAACACCTG  650
            ||||||||||||||||||||||||||||||||||||||||||||||||||
Sbjct  630  CATTCTTTGACCCTGCAGGAGGAGGGGATCCAATCCTCTACCAACACCTG  679
```

## Taxonomy

### Reports

- #### Lineage

| Organism | Blast Name | Score | Number of Hits | Description |
|---|---|---|---|---|
| Bilateria | animals | | 126 | |
| .Actinopterygii | bony fishes | | 125 | |
| ..Pleuronectoidei | bony fishes | | 92 | |
| ...Paralichthys olivaceus x Verasper variegatus | bony fishes | 1201 | 2 | **Paralichthys olivaceus x Verasper variegatus hits** |
| ...Paralichthys olivaceus | bony fishes | 1201 | 31 | **Paralichthys olivaceus hits** |
| ...Paralichthys aestuarius | bony fishes | 819 | 1 | **Paralichthys aestuarius hits** |
| ...Paralichthys squamilentus | bony fishes | 808 | 1 | **Paralichthys squamilentus hits** |
| ...Paralichthys patagonicus | bony fishes | 808 | 4 | **Paralichthys patagonicus hits** |
| ...Paralichthys adspersus | bony fishes | 802 | 2 | **Paralichthys adspersus hits** |
| ...Paralichthys californicus | bony fishes | 774 | 7 | **Paralichthys californicus hits** |
| ...Paralichthys dentatus | bony fishes | 734 | 18 | **Paralichthys dentatus hits** |
| ...Paralichthys woolmani | bony fishes | 728 | 2 | **Paralichthys woolmani hits** |
| ...Paralichthys albigutta | bony fishes | 725 | 6 | **Paralichthys albigutta hits** |
| ...Paralichthys orbignyanus | bony fishes | 719 | 2 | **Paralichthys orbignyanus hits** |
| ...Paralichthyidae sp. BOLD:AAO1988 | bony fishes | 706 | 1 | **Paralichthyidae sp. BOLD:AAO1988 hits** |
| ...Xystreurys rasile | bony fishes | 691 | 8 | **Xystreurys rasile hits** |
| ...Paralichthys isosceles | bony fishes | 686 | 2 | **Paralichthys isosceles hits** |
| ...Verasper moseri | bony fishes | 678 | 5 | **Verasper moseri hits** |
| ..Actinopterygii environmental sample | bony fishes | 734 | 33 | **Actinopterygii environmental sample hits** |
| .Penaeus vannamei | crustaceans | 680 | 1 | **Penaeus vannamei hits** |

- #### Organism

| Description | Score | E value | Accession |
|---|---|---|---|
| Paralichthys olivaceus x Verasper variegatus [bony fishes ] | | | |

| Description | Score | E value | Accession |
|---|---|---|---|
| Paralichthys olivaceus x Verasper variegatus mitochondrion, complete genome | 1201 | 0.0 | NC_082846 |
| Paralichthys olivaceus x Verasper variegatus mitochondrion, complete genome | 1201 | 0.0 | OR353704 |
| Paralichthys olivaceus (Japanese flounder) [bony fishes ] | | | |
| Paralichthys olivaceus voucher XM073 cytochrome oxidase subunit I (COI) gene, partial cds; mitochondrial | 1201 | 0.0 | KX254476 |
| Paralichthys olivaceus voucher HME1 cytochrome oxidase subunit 1 (COI) gene, partial cds; mitochondrial | 1201 | 0.0 | JF952803 |
| Paralichthys olivaceus isolate CO1B7 cytochrome oxidase subunit I-like (COI) gene, partial sequence; mitochondrial | 1201 | 0.0 | EU266369 |
| Paralichthys olivaceus voucher HUM I-00297 cytochrome c oxidase subunit 1 (COI) gene, complete cds; mitochondrial | 1195 | 0.0 | MH032483 |
| Paralichthys olivaceus voucher XM072 cytochrome oxidase subunit I (COI) gene, partial cds; mitochondrial | 1195 | 0.0 | KX254475 |
| Paralichthys olivaceus voucher TWS 029 cytochrome oxidase subunit I (COI) gene, partial cds; mitochondrial | 1195 | 0.0 | MG220576 |
| Paralichthys olivaceus voucher HUM I-00296 cytochrome c oxidase subunit 1 (COI) gene, complete cds; mitochondrial | 1190 | 0.0 | MH032482 |
| Paralichthys olivaceus voucher XM074 cytochrome oxidase subunit I (COI) gene, partial cds; mitochondrial | 1190 | 0.0 | KX254477 |
| Paralichthys olivaceus voucher HME2 cytochrome oxidase subunit 1 (COI) gene, partial cds; mitochondrial | 1190 | 0.0 | JF952804 |
| Paralichthys olivaceus isolate CO1B8 cytochrome oxidase subunit I (COI) gene, partial cds; mitochondrial | 1190 | 0.0 | EU266368 |
| Paralichthys olivaceus mitochondrion, complete genome | 1190 | 0.0 | NC_002386 |
| Paralichthys olivaceus mitochondrial DNA, complete genome | 1190 | 0.0 | AB028664 |
| Paralichthys olivaceus isolate WJC173 cytochrome oxidase subunit 1 (COI) gene, partial cds; mitochondrial | 1173 | 0.0 | MK617161 |
| Paralichthys olivaceus strain West.5.3 cytochrome c oxidase subunit I (COI) gene, partial cds; mitochondrial | 1173 | 0.0 | MK560569 |
| Paralichthys olivaceus voucher IOCASFY-RCB09-Po5 cytochrome oxidase subunit I (COI) gene, partial cds; mitochondrial | 1146 | 0.0 | KU236831 |
| Paralichthys olivaceus voucher IOCASFY-RCB09-Po1 cytochrome oxidase subunit I (COI) gene, partial cds; mitochondrial | 1134 | 0.0 | KU236827 |
| Paralichthys olivaceus voucher IOCASFY-RCB09-Po2 cytochrome oxidase subunit I (COI) gene, partial cds; mitochondrial | 1134 | 0.0 | KU236828 |
| Paralichthys olivaceus voucher IOCASFY-RCB09-Po3 cytochrome oxidase subunit I (COI) gene, partial cds; mitochondrial | 1134 | 0.0 | KU236829 |
| Paralichthys olivaceus voucher IOCASFY-RCB09-Po4 cytochrome oxidase subunit I (COI) gene, partial cds; mitochondrial | 1134 | 0.0 | KU236830 |
| Paralichthys olivaceus voucher IOCASFY-RCB09-Po6 cytochrome oxidase subunit I (COI) gene, partial cds; mitochondrial | 1134 | 0.0 | KU236832 |
| Paralichthys olivaceus voucher IOCASFY-RCB09-Po7 cytochrome oxidase subunit I (COI) gene, partial cds; mitochondrial | 1129 | 0.0 | KU236833 |
| Paralichthys olivaceus isolate j4 cytochrome c oxidase subunit I (COX1) gene, partial cds; mitochondrial | 1109 | 0.0 | MZ317453 |
| Paralichthys olivaceus isolate PKU_10872 cytochrome oxidase subunit I (COI) gene, partial cds; mitochondrial | 1101 | 0.0 | KP835312 |
| Paralichthys olivaceus isolate ASIZP0914829 cytochrome oxidase subunit I (COI) gene, partial cds; mitochondrial | 1003 | 0.0 | KU945107 |
| Paralichthys olivaceus isolate F00223 cytochrome oxidase subunit I (COI) gene, partial cds; mitochondrial | 1002 | 0.0 | JQ738445 |
| Paralichthys olivaceus isolate sample_77 cytochrome c oxidase subunit I (COI) gene, partial cds; mitochondrial | 965 | 0.0 | MW027188 |
| Paralichthys olivaceus isolate PKU 4489 cytochrome oxidase subunit I (COI) gene, partial cds; mitochondrial | 946 | 0.0 | KR052269 |
| Paralichthys olivaceus mitochondrial COX1 gene for cytochrome c oxidase subunit 1, partial cds, isolate: 005_55 | 915 | 0.0 | LC126342 |
| Paralichthys olivaceus isolate PKU 4490 cytochrome oxidase subunit I gene, partial cds; mitochondrial | 843 | 0.0 | KF965424 |

| Description | Score | E value | Accession |
|---|---|---|---|
| Paralichthys olivaceus isolate PKU 4489 cytochrome oxidase subunit I gene, partial cds; mitochondrial | 837 | 0.0 | KF965438 |
| Paralichthys olivaceus isolate F00222 cytochrome oxidase subunit I (COI) gene, partial cds; mitochondrial | 813 | 0.0 | JQ738444 |
| Paralichthys aestuarius (Cortez flounder) [bony fishes ] | | | |
| Paralichthys aestuarius voucher SIO 07-157 cytochrome c oxidase subunit 1 (COI) gene, complete cds; mitochondrial | 819 | 0.0 | MH032480 |
| Paralichthys squamilentus (broad flounder) [bony fishes ] | | | |
| Paralichthys squamilentus voucher KUT 5205 cytochrome oxidase subunit 1 (COI) gene, partial cds; mitochondrial | 808 | 0.0 | KF930230 |
| Paralichthys patagonicus (Patagonian flounder) [bony fishes ] | | | |
| Paralichthys patagonicus voucher HRCB:53035 cytochrome oxidase subunit 1 (COI) gene, partial cds; mitochondrial | 808 | 0.0 | JQ365477 |
| Paralichthys patagonicus voucher DAAPV F28 cytochrome oxidase subunit I (COI) gene, partial cds; mitochondrial | 798 | 0.0 | GU324191 |
| Paralichthys patagonicus voucher HRCB:53034 cytochrome oxidase subunit 1 (COI) gene, partial cds; mitochondrial | 787 | 0.0 | JQ365478 |
| Paralichthys patagonicus voucher LBPV53033 cytochrome oxidase subunit 1 (COI) gene, partial cds; mitochondrial | 787 | 0.0 | JX124847 |
| Paralichthys adspersus (fine flounder) [bony fishes ] | | | |
| Paralichthys adspersus mitochondrion, complete genome | 802 | 0.0 | NC_057273 |
| Paralichthys adspersus mitochondrion, complete genome | 802 | 0.0 | MW288827 |
| Paralichthys californicus (California flounder) [bony fishes ] | | | |
| Paralichthys californicus voucher SIO 03-51 cytochrome c oxidase subunit 1 (COI) gene, complete cds; mitochondrial | 774 | 0.0 | MH032481 |
| Paralichthys californicus mitochondrion, complete genome | 763 | 0.0 | MT859134 |
| Paralichthys californicus voucher MFC144 cytochrome oxidase subunit 1 (COI) gene, partial cds; mitochondrial | 763 | 0.0 | GU440447 |
| Paralichthys californicus voucher KUT 456 cytochrome oxidase subunit 1 (COI) gene, partial cds; mitochondrial | 763 | 0.0 | KF930225 |
| Paralichthys californicus voucher JUPA-822 cytochrome oxidase subunit 1 (COI) gene, partial cds; mitochondrial | 686 | 0.0 | KM077536 |
| Paralichthys californicus voucher JUPA-823 cytochrome oxidase subunit 1 (COI) gene, partial cds; mitochondrial | 682 | 0.0 | KM019390 |
| Paralichthys californicus voucher JUPA-827 cytochrome oxidase subunit 1 (COI) gene, partial cds; mitochondrial | 682 | 0.0 | KM019385 |
| Paralichthys dentatus (summer flounder) [bony fishes ] | | | |
| Paralichthys dentatus voucher USNM:FISH:431132 cytochrome oxidase subunit 1 (COI) gene, partial cds; mitochondrial | 734 | 0.0 | MT455401 |
| Paralichthys dentatus voucher 07-045 cytochrome oxidase subunit 1 (COI) gene, partial cds; mitochondrial | 734 | 0.0 | KC015757 |
| Paralichthys dentatus voucher 09-399 cytochrome oxidase subunit 1 (COI) gene, partial cds; mitochondrial | 734 | 0.0 | KC015760 |
| Paralichthys dentatus voucher 07-123 cytochrome oxidase subunit 1 (COI) gene, partial cds; mitochondrial | 730 | 0.0 | KC015758 |
| Paralichthys dentatus voucher USNM:FISH:423822 cytochrome oxidase subunit 1 (COI) gene, partial cds; mitochondrial | 728 | 0.0 | MT455243 |
| Paralichthys dentatus voucher USNM:FISH:423835 cytochrome oxidase subunit 1 (COI) gene, partial cds; mitochondrial | 728 | 0.0 | MT455651 |
| Paralichthys dentatus voucher USNM:FISH:423812 cytochrome oxidase subunit 1 (COI) gene, partial cds; mitochondrial | 728 | 0.0 | MT455665 |
| Paralichthys dentatus voucher USNM:FISH:423932 cytochrome oxidase subunit 1 (COI) gene, partial cds; mitochondrial | 728 | 0.0 | MT455883 |
| Paralichthys dentatus mitochondrion, complete genome | 728 | 0.0 | NC_029476 |
| Paralichthys dentatus mitochondrion, complete genome | 728 | 0.0 | KU053334 |
| Paralichthys dentatus voucher 06-117 cytochrome oxidase subunit 1 (COI) gene, partial cds; mitochondrial | 728 | 0.0 | KC015755 |
| Paralichthys dentatus voucher 07-092 cytochrome oxidase subunit 1 (COI) gene, partial cds; mitochondrial | 728 | 0.0 | KC015759 |

| Description | Score | E value | Accession |
|---|---|---|---|
| Paralichthys dentatus voucher 09-400 cytochrome oxidase subunit 1 (COI) gene, partial cds; mitochondrial | 728 | 0.0 | KC015756 |
| Paralichthys dentatus voucher USNM:FISH:423832 cytochrome oxidase subunit 1 (COI) gene, partial cds; mitochondrial | 725 | 0.0 | MT456241 |
| Paralichthys dentatus voucher USNM:FISH:423933 cytochrome oxidase subunit 1 (COI) gene, partial cds; mitochondrial | 723 | 0.0 | MT455927 |
| Paralichthys dentatus voucher USNM:FISH:423811 cytochrome oxidase subunit 1 (COI) gene, partial cds; mitochondrial | 723 | 0.0 | MT455323 |
| Paralichthys dentatus voucher 06-099 cytochrome oxidase subunit 1 (COI) gene, partial cds; mitochondrial | 710 | 0.0 | KC015754 |
| Paralichthys dentatus cytochrome oxidase subunit I (COI) gene, partial cds; mitochondrial | 686 | 0.0 | KX164002 |
| Actinopterygii environmental sample [bony fishes ] | | | |
| Actinopterygii environmental sample voucher DE1109058_05 cytochrome oxidase subunit 1 (COI) gene, partial cds; mitochondrial | 734 | 0.0 | KP110988 |
| Actinopterygii environmental sample voucher DE1109067_02 cytochrome oxidase subunit 1 (COI) gene, partial cds; mitochondrial | 734 | 0.0 | KP111602 |
| Actinopterygii environmental sample voucher DE1109057_01 cytochrome oxidase subunit 1 (COI) gene, partial cds; mitochondrial | 734 | 0.0 | KP111781 |
| Actinopterygii environmental sample voucher DE1109057_03 cytochrome oxidase subunit 1 (COI) gene, partial cds; mitochondrial | 734 | 0.0 | KP112034 |
| Actinopterygii environmental sample voucher DE1109058_01 cytochrome oxidase subunit 1 (COI) gene, partial cds; mitochondrial | 728 | 0.0 | KP111879 |
| Actinopterygii environmental sample voucher DE1109058_02 cytochrome oxidase subunit 1 (COI) gene, partial cds; mitochondrial | 728 | 0.0 | KP110848 |
| Actinopterygii environmental sample voucher DE1109067_01 cytochrome oxidase subunit 1 (COI) gene, partial cds; mitochondrial | 728 | 0.0 | KP111573 |
| Actinopterygii environmental sample voucher DL0706_054_04 cytochrome oxidase subunit 1 (COI) gene, partial cds; mitochondrial | 725 | 0.0 | KP111797 |
| Actinopterygii environmental sample voucher DE1109067_03 cytochrome oxidase subunit 1 (COI) gene, partial cds; mitochondrial | 723 | 0.0 | KP111488 |
| Actinopterygii environmental sample voucher DL0706_054_03 cytochrome oxidase subunit 1 (COI) gene, partial cds; mitochondrial | 721 | 0.0 | KP110873 |
| Actinopterygii environmental sample voucher DE0210_011_05 cytochrome oxidase subunit 1 (COI) gene, partial cds; mitochondrial | 715 | 0.0 | KP111710 |
| Actinopterygii environmental sample voucher DE0210_011_02 cytochrome oxidase subunit 1 (COI) gene, partial cds; mitochondrial | 712 | 0.0 | KP111827 |
| Actinopterygii environmental sample voucher DE0210_011_06 cytochrome oxidase subunit 1 (COI) gene, partial cds; mitochondrial | 712 | 0.0 | KP111283 |
| Actinopterygii environmental sample voucher DE0210_002_02 cytochrome oxidase subunit 1 (COI) gene, partial cds; mitochondrial | 712 | 0.0 | KP111187 |
| Actinopterygii environmental sample voucher DE0210_011_03 cytochrome oxidase subunit 1 (COI) gene, partial cds; mitochondrial | 710 | 0.0 | KP111956 |
| Actinopterygii environmental sample voucher DE0210_011_01 cytochrome oxidase subunit 1 (COI) gene, partial cds; mitochondrial | 710 | 0.0 | KP111944 |
| Actinopterygii environmental sample voucher DE0911_037_01 cytochrome oxidase subunit 1 (COI) gene, partial cds; mitochondrial | 710 | 0.0 | KP111360 |
| Actinopterygii environmental sample voucher DL0711_005_07 cytochrome oxidase subunit 1 (COI) gene, partial cds; mitochondrial | 710 | 0.0 | KP111091 |
| Actinopterygii environmental sample voucher DE0210_011_07 cytochrome oxidase subunit 1 (COI) gene, partial cds; mitochondrial | 710 | 0.0 | KP110799 |
| Actinopterygii environmental sample voucher DL0711_005_05 cytochrome oxidase subunit 1 (COI) gene, partial cds; mitochondrial | 706 | 0.0 | KP111700 |
| Actinopterygii environmental sample voucher AL0410_053_01 cytochrome oxidase subunit 1 (COI) gene, partial cds; mitochondrial | 706 | 0.0 | KP111235 |
| Actinopterygii environmental sample voucher DL0711_034_01 cytochrome oxidase subunit 1 (COI) gene, partial cds; mitochondrial | 704 | 0.0 | KP111750 |
| Actinopterygii environmental sample voucher DL0711_005_06 cytochrome oxidase subunit 1 (COI) gene, partial cds; mitochondrial | 704 | 0.0 | KP111041 |
| Actinopterygii environmental sample voucher DL0711_056_08 cytochrome oxidase subunit 1 (COI) gene, partial cds; mitochondrial | 704 | 0.0 | KP110830 |

| Description | Score | E value | Accession |
|---|---|---|---|
| Actinopterygii environmental sample voucher AL0410_028_04 cytochrome oxidase subunit 1 (COI) gene, partial cds; mitochondrial | 702 | 0.0 | KP111841 |
| Actinopterygii environmental sample voucher AL0410_028_09 cytochrome oxidase subunit 1 (COI) gene, partial cds; mitochondrial | 702 | 0.0 | KP110854 |
| Actinopterygii environmental sample voucher DL0711_056_01 cytochrome oxidase subunit 1 (COI) gene, partial cds; mitochondrial | 701 | 0.0 | KP111741 |
| Actinopterygii environmental sample voucher DL0711_056_03 cytochrome oxidase subunit 1 (COI) gene, partial cds; mitochondrial | 701 | 0.0 | KP111179 |
| Actinopterygii environmental sample voucher DL0711_056_07 cytochrome oxidase subunit 1 (COI) gene, partial cds; mitochondrial | 701 | 0.0 | KP111103 |
| Actinopterygii environmental sample voucher AL0410_028_03 cytochrome oxidase subunit 1 (COI) gene, partial cds; mitochondrial | 699 | 0.0 | KP112075 |
| Actinopterygii environmental sample voucher DL0711_056_06 cytochrome oxidase subunit 1 (COI) gene, partial cds; mitochondrial | 699 | 0.0 | KP111884 |
| Actinopterygii environmental sample voucher DL0711_005_02 cytochrome oxidase subunit 1 (COI) gene, partial cds; mitochondrial | 697 | 0.0 | KP111731 |
| Actinopterygii environmental sample voucher DE0210_011_04 cytochrome oxidase subunit 1 (COI) gene, partial cds; mitochondrial | 697 | 0.0 | KP111115 |
| Paralichthys woolmani (speckled flounder) [bony fishes ] | | | |
| Paralichthys woolmani voucher SIO-09-205 cytochrome c oxidase subunit 1 (COI) gene, complete cds; mitochondrial | 728 | 0.0 | MH032484 |
| Paralichthys woolmani isolate LPZSF105 cytochrome c oxidase subunit I (COX1) gene, partial cds; mitochondrial | 693 | 0.0 | MT311625 |
| Paralichthys albigutta (Gulf flounder) [bony fishes ] | | | |
| Paralichthys albigutta voucher FDA 103 cytochrome oxidase subunit 1 (COI) gene, partial cds; mitochondrial | 725 | 0.0 | KF461215 |
| Paralichthys albigutta voucher MXV0035 cytochrome oxidase subunit 1 (COI) gene, partial cds; mitochondrial | 723 | 0.0 | MG837970 |
| Paralichthys albigutta voucher USNM:FISH:447358 cytochrome oxidase subunit 1 (COI) gene, partial cds; mitochondrial | 719 | 0.0 | MT455829 |
| Paralichthys albigutta voucher USNM:FISH:454751 mitochondrion, complete genome | 719 | 0.0 | NC_083031 |
| Paralichthys albigutta voucher USNM:FISH:454751 mitochondrion, complete genome | 719 | 0.0 | OR546183 |
| Paralichthys albigutta voucher USNM:FISH:451250 cytochrome oxidase subunit 1 (COI) gene, partial cds; mitochondrial | 713 | 0.0 | MT455012 |
| Paralichthys orbignyanus [bony fishes ] | | | |
| Paralichthys orbignyanus isolate FARG322-07 cytochrome oxidase subunit I (COI) gene, partial cds; mitochondrial | 719 | 0.0 | EU074519 |
| Paralichthys orbignyanus isolate FARG286-07 cytochrome oxidase subunit I (COI) gene, partial cds; mitochondrial | 719 | 0.0 | EU074520 |
| Paralichthyidae sp. BOLD:AAO1988 [bony fishes ] | | | |
| Paralichthyidae sp. BOLD:AAO1988 voucher SMSA7168 cytochrome oxidase subunit 1 (COI) gene, partial cds; mitochondrial | 706 | 0.0 | JQ842632 |
| Xystreurys rasile [bony fishes ] | | | |
| Xystreurys rasile isolate FARG358-07 cytochrome oxidase subunit I (COI) gene, partial cds; mitochondrial | 691 | 0.0 | EU074623 |
| Xystreurys rasile isolate FARG218-06 cytochrome oxidase subunit I (COI) gene, partial cds; mitochondrial | 686 | 0.0 | EU074629 |
| Xystreurys rasile isolate FARG220-06 cytochrome oxidase subunit I (COI) gene, partial cds; mitochondrial | 686 | 0.0 | EU074627 |
| Xystreurys rasile isolate FARG217-06 cytochrome oxidase subunit I (COI) gene, partial cds; mitochondrial | 686 | 0.0 | EU074624 |
| Xystreurys rasile isolate FARG219-06 cytochrome oxidase subunit I (COI) gene, partial cds; mitochondrial | 680 | 0.0 | EU074626 |
| Xystreurys rasile isolate FARG221-06 cytochrome oxidase subunit I (COI) gene, partial cds; mitochondrial | 680 | 0.0 | EU074628 |
| Xystreurys rasile isolate FARG216-06 cytochrome oxidase subunit I (COI) gene, partial cds; mitochondrial | 680 | 0.0 | EU074625 |
| Xystreurys rasile isolate FARG359-07 cytochrome oxidase subunit I (COI) gene, partial cds; mitochondrial | 680 | 0.0 | EU074622 |

| Description | Score | E value | Accession |
|---|---|---|---|
| Paralichthys isosceles [bony fishes ] | | | |
| Paralichthys isosceles voucher HRCB:46871 cytochrome oxidase subunit 1 (COI) gene, partial cds; mitochondrial | 686 | 0.0 | JQ365476 |
| Paralichthys isosceles voucher HRCB:46870 cytochrome oxidase subunit 1 (COI) gene, partial cds; mitochondrial | 686 | 0.0 | JQ365475 |
| Penaeus vannamei (Pacific white shrimp) [crustaceans ] | | | |
| Litopenaeus vannamei voucher DAAPV F14 cytochrome oxidase subunit I (COI) gene, partial cds; mitochondrial | 680 | 0.0 | GU324180 |
| Verasper moseri (barfin flounder) [bony fishes ] | | | |
| Verasper moseri voucher UW 118097 cytochrome c oxidase subunit 1 (COI) gene, complete cds; mitochondrial | 678 | 0.0 | MH032541 |
| Verasper moseri voucher UW 118096 cytochrome c oxidase subunit 1 (COI) gene, complete cds; mitochondrial | 678 | 0.0 | MH032540 |
| Verasper moseri mitochondrion, complete genome | 678 | 0.0 | LC583747 |
| Verasper moseri mitochondrion, complete genome | 678 | 0.0 | NC_008461 |
| Verasper moseri mitochondrion, complete genome | 678 | 0.0 | EF025506 |

- ## Taxonomy

| Taxonomy | Number of hits | Number of Organisms | Description |
|---|---|---|---|
| Bilateria | 126 | 17 | |
| . Actinopterygii | 125 | 16 | |
| .. Pleuronectoidei | 92 | 15 | |
| ... Paralichthys olivaceus x Verasper variegatus | 2 | 1 | Paralichthys olivaceus x Verasper variegatus hits |
| ... Paralichthyidae | 85 | 13 | |
| .... Paralichthys | 76 | 11 | |
| ..... Paralichthys olivaceus | 31 | 1 | Paralichthys olivaceus hits |
| ..... Paralichthys aestuarius | 1 | 1 | Paralichthys aestuarius hits |
| ..... Paralichthys squamilentus | 1 | 1 | Paralichthys squamilentus hits |
| ..... Paralichthys patagonicus | 4 | 1 | Paralichthys patagonicus hits |
| ..... Paralichthys adspersus | 2 | 1 | Paralichthys adspersus hits |
| ..... Paralichthys californicus | 7 | 1 | Paralichthys californicus hits |
| ..... Paralichthys dentatus | 18 | 1 | Paralichthys dentatus hits |
| ..... Paralichthys woolmani | 2 | 1 | Paralichthys woolmani hits |
| ..... Paralichthys albigutta | 6 | 1 | Paralichthys albigutta hits |
| ..... Paralichthys orbignyanus | 2 | 1 | Paralichthys orbignyanus hits |
| ..... Paralichthys isosceles | 2 | 1 | Paralichthys isosceles hits |
| .... Paralichthyidae sp. BOLD:AAO1988 | 1 | 1 | Paralichthyidae sp. BOLD:AAO1988 hits |
| .... Xystreurys rasile | 8 | 1 | Xystreurys rasile hits |
| ... Verasper moseri | 5 | 1 | Verasper moseri hits |
| .. Actinopterygii environmental sample | 33 | 1 | Actinopterygii environmental sample hits |
| . Penaeus vannamei | 1 | 1 | Penaeus vannamei hits |

Top

Follow NCBI

Connect with NLM

National Library of Medicine
8600 Rockville Pike
Bethesda, MD 20894

Web Policies
FOIA
HHS Vulnerability Disclosure

Help
Accessibility
Careers

- NLM
- NIH
- HHS
- USA.gov
