# Supplementary material for: Establishment and Characterization of OFT and OFO Cell Lines from Olive Flounder (Paralichthys olivaceus) for Use as Feeder Cells
Source: Biology (Basel). 2025 Feb 24;14(3):229. doi: 10.3390/biology14030229 (PMC11939788; doi:10.3390/biology14030229)
Supplement: Supplementary file 1 [file biology-14-00229-s001.zip › NCBI Blast_COI sequencing_OFT.pdf]

An official website of the United States government

Here's how you know

The .gov means it’s official.
Federal government websites often end in .gov or .mil. Before sharing sensitive information, make sure you’re on a federal government site.

Feedback

The site is secure.
The **https://** ensures that you are connecting to the official website and that any information you provide is encrypted and transmitted securely.
Access keys NCBI Homepage MyNCBI Homepage Main Content Main Navigation

BLAST ® » blastn suite » results for RID-09E1SU9T013

**Job Title**	FISHR2-01.ab1 ...
**RID**	09E1SU9T013 Search expires on 03-29 08:22 am
**Results for**	3:lcl|Query_20006 VF2-01.ab1(650bp)
**Program**	BLASTN
**Database**	nt
**Query ID**	lcl|Query_20006
**Description**	VF2-01.ab1 ...
**Molecule type**	dna
**Query Length**	650

Descriptions

Description	Scientific Name	Max Score	Total Score	Query Cover	E value	Per. Ident	Acc. Len	Accession
Paralichthys olivaceus x Verasper variegatus mitochondrion, complete genome	Paralichthys olivaceus x Verasper variegatus	1201	1201	100%	0.0	100.00%	16946	NC_082846.1
Paralichthys olivaceus voucher XM073 cytochrome oxidase subunit I (COI) gene, partial cds; mitochondrial	Paralichthys olivaceus	1201	1201	100%	0.0	100.00%	655	KX254476.1
Paralichthys olivaceus voucher HME1 cytochrome oxidase subunit 1 (COI) gene, partial cds; mitochondrial	Paralichthys olivaceus	1201	1201	100%	0.0	100.00%	652	JF952803.1
Paralichthys olivaceus isolate CO1B7 cytochrome oxidase subunit I-like (COI) gene, partial sequence; mitochondrial	Paralichthys olivaceus	1201	1201	100%	0.0	100.00%	713	EU266369.1
Paralichthys olivaceus voucher HUM I-00297 cytochrome c oxidase subunit 1 (COI) gene, complete cds; mitochondrial	Paralichthys olivaceus	1195	1195	100%	0.0	99.85%	1551	MH032483.1
Paralichthys olivaceus voucher XM072 cytochrome oxidase subunit I (COI) gene, partial cds; mitochondrial	Paralichthys olivaceus	1195	1195	100%	0.0	99.85%	655	KX254475.1
Paralichthys olivaceus voucher HUM I-00296 cytochrome c oxidase subunit 1 (COI) gene, complete cds; mitochondrial	Paralichthys olivaceus	1190	1190	100%	0.0	99.69%	1551	MH032482.1
Paralichthys olivaceus voucher XM074 cytochrome oxidase subunit I (COI) gene, partial cds; mitochondrial	Paralichthys olivaceus	1190	1190	100%	0.0	99.69%	655	KX254477.1
Paralichthys olivaceus voucher HME2 cytochrome oxidase subunit 1 (COI) gene, partial cds; mitochondrial	Paralichthys olivaceus	1190	1190	100%	0.0	99.69%	652	JF952804.1
Paralichthys olivaceus isolate CO1B8 cytochrome oxidase subunit I (COI) gene, partial cds; mitochondrial	Paralichthys olivaceus	1190	1190	100%	0.0	99.69%	710	EU266368.1
Paralichthys olivaceus mitochondrion, complete genome	Paralichthys olivaceus	1190	1190	100%	0.0	99.69%	17090	NC_002386.1
Paralichthys olivaceus isolate WJC173 cytochrome oxidase subunit 1 (COI) gene, partial cds; mitochondrial	Paralichthys olivaceus	1173	1173	98%	0.0	99.69%	642	MK617161.1
Paralichthys olivaceus strain West.5.3 cytochrome c	Paralichthys olivaceus	1173	1173	99%	0.0	99.38%	647	MK560569.1

Description	Scientific Name	Max Score	Total Score	Query Cover	E value	Per. Ident	Acc. Len	Accession
oxidase subunit I (COI) gene, partial cds; mitochondrial								
Paralichthys olivaceus voucher IOCASFY-RCB09-Po5 cytochrome oxidase subunit I (COI) gene, partial cds; mitochondrial	Paralichthys olivaceus	1146	1146	95%	0.0	100.00%	620	KU236831.1
Paralichthys olivaceus voucher IOCASFY-RCB09-Po1 cytochrome oxidase subunit I (COI) gene, partial cds; mitochondrial	Paralichthys olivaceus	1134	1134	95%	0.0	99.68%	620	KU236827.1
Paralichthys olivaceus voucher IOCASFY-RCB09-Po7 cytochrome oxidase subunit I (COI) gene, partial cds; mitochondrial	Paralichthys olivaceus	1129	1129	95%	0.0	99.52%	620	KU236833.1
Paralichthys olivaceus isolate j4 cytochrome c oxidase subunit I (COX1) gene, partial cds; mitochondrial	Paralichthys olivaceus	1109	1109	93%	0.0	99.51%	617	MZ317453.1
Paralichthys olivaceus isolate PKU_10872 cytochrome oxidase subunit I (COI) gene, partial cds; mitochondrial	Paralichthys olivaceus	1101	1101	92%	0.0	99.67%	604	KP835312.1
Paralichthys olivaceus isolate ASIZP0914829 cytochrome oxidase subunit I (COI) gene, partial cds; mitochondrial	Paralichthys olivaceus	1003	1003	84%	0.0	99.46%	552	KU945107.1
Paralichthys olivaceus isolate F00223 cytochrome oxidase subunit I (COI) gene, partial cds; mitochondrial	Paralichthys olivaceus	1002	1002	92%	0.0	96.83%	612	JQ738445.1
Paralichthys olivaceus isolate sample_77 cytochrome c oxidase subunit I (COI) gene, partial cds; mitochondrial	Paralichthys olivaceus	965	965	80%	0.0	100.00%	522	MW027188.1
Paralichthys olivaceus isolate PKU 4489 cytochrome oxidase subunit I (COI) gene, partial cds; mitochondrial	Paralichthys olivaceus	946	946	79%	0.0	99.81%	515	KR052269.1
Paralichthys olivaceus mitochondrial COX1 gene for cytochrome c oxidase subunit 1, partial cds, isolate: 005_55	Paralichthys olivaceus	915	915	77%	0.0	99.60%	501	LC126342.1
Paralichthys olivaceus isolate PKU 4490 cytochrome oxidase subunit I gene,	Paralichthys olivaceus	843	843	70%	0.0	100.00%	456	KF965424.1

Description	Scientific Name	Max Score	Total Score	Query Cover	E value	Per. Ident	Acc. Len	Accession
partial cds; mitochondrial								
Paralichthys olivaceus isolate PKU 4489 cytochrome oxidase subunit I gene, partial cds; mitochondrial	Paralichthys olivaceus	837	837	70%	0.0	99.78%	456	KF965438.1
Paralichthys aestuarius voucher SIO 07-157 cytochrome c oxidase subunit 1 (COI) gene, complete cds; mitochondrial	Paralichthys aestuarius	819	819	100%	0.0	89.38%	1551	MH032480.1
Paralichthys olivaceus isolate F00222 cytochrome oxidase subunit I (COI) gene, partial cds; mitochondrial	Paralichthys olivaceus	813	813	68%	0.0	99.55%	448	JQ738444.1
Paralichthys squamilentus voucher KUT 5205 cytochrome oxidase subunit 1 (COI) gene, partial cds; mitochondrial	Paralichthys squamilentus	808	808	99%	0.0	89.18%	652	KF930230.1
Paralichthys patagonicus voucher HRCB:53035 cytochrome oxidase subunit 1 (COI) gene, partial cds; mitochondrial	Paralichthys patagonicus	808	808	100%	0.0	89.08%	652	JQ365477.1
Paralichthys adspersus mitochondrion, complete genome	Paralichthys adspersus	802	802	100%	0.0	88.92%	17060	NC_057273.1
Paralichthys patagonicus voucher DAAPV F28 cytochrome oxidase subunit I (COI) gene, partial cds; mitochondrial	Paralichthys patagonicus	798	798	99%	0.0	88.89%	652	GU324191.1
Paralichthys patagonicus voucher HRCB:53034 cytochrome oxidase subunit 1 (COI) gene, partial cds; mitochondrial	Paralichthys patagonicus	787	787	99%	0.0	88.58%	652	JQ365478.1
Paralichthys californicus voucher SIO 03-51 cytochrome c oxidase subunit 1 (COI) gene, complete cds; mitochondrial	Paralichthys californicus	774	774	100%	0.0	88.15%	1551	MH032481.1
Paralichthys californicus mitochondrion, complete genome	Paralichthys californicus	763	763	100%	0.0	87.85%	16858	MT859134.1
Paralichthys californicus voucher MFC144 cytochrome oxidase subunit 1 (COI) gene, partial cds; mitochondrial	Paralichthys californicus	763	763	100%	0.0	87.85%	652	GU440447.1
Paralichthys dentatus voucher USNM:FISH:431132 cytochrome oxidase subunit 1 (COI) gene,	Paralichthys dentatus	734	734	99%	0.0	87.06%	655	MT455401.1

Description	Scientific Name	Max Score	Total Score	Query Cover	E value	Per. Ident	Acc. Len	Accession
partial cds; mitochondrial								
Actinopterygii environmental sample voucher DE1109058_05 cytochrome oxidase subunit 1 (COI) gene, partial cds; mitochondrial	Actinopterygii environmental sample	734	734	99%	0.0	87.06%	652	KP110988.1
Paralichthys dentatus voucher 07-045 cytochrome oxidase subunit 1 (COI) gene, partial cds; mitochondrial	Paralichthys dentatus	734	734	99%	0.0	87.06%	652	KC015757.1
Paralichthys dentatus voucher 07-123 cytochrome oxidase subunit 1 (COI) gene, partial cds; mitochondrial	Paralichthys dentatus	730	730	99%	0.0	86.90%	652	KC015758.1
Paralichthys dentatus voucher USNM:FISH:423822 cytochrome oxidase subunit 1 (COI) gene, partial cds; mitochondrial	Paralichthys dentatus	728	728	99%	0.0	86.90%	655	MT455243.1
Paralichthys woolmani voucher SIO-09-205 cytochrome c oxidase subunit 1 (COI) gene, complete cds; mitochondrial	Paralichthys woolmani	728	728	99%	0.0	86.90%	1551	MH032484.1
Paralichthys dentatus mitochondrion, complete genome	Paralichthys dentatus	728	728	99%	0.0	86.90%	17033	NC_029476.1
Actinopterygii environmental sample voucher DE1109058_01 cytochrome oxidase subunit 1 (COI) gene, partial cds; mitochondrial	Actinopterygii environmental sample	728	728	99%	0.0	86.90%	652	KP111879.1
Paralichthys dentatus voucher 06-117 cytochrome oxidase subunit 1 (COI) gene, partial cds; mitochondrial	Paralichthys dentatus	728	728	99%	0.0	86.90%	652	KC015755.1
Paralichthys dentatus voucher 09-400 cytochrome oxidase subunit 1 (COI) gene, partial cds; mitochondrial	Paralichthys dentatus	728	728	99%	0.0	86.90%	652	KC015756.1
Paralichthys dentatus voucher USNM:FISH:423832 cytochrome oxidase subunit 1 (COI) gene, partial cds; mitochondrial	Paralichthys dentatus	725	725	99%	0.0	86.86%	649	MT456241.1
Actinopterygii environmental sample voucher DL0706_054_04 cytochrome oxidase subunit 1 (COI) gene, partial cds; mitochondrial	Actinopterygii environmental sample	725	725	99%	0.0	86.96%	646	KP111797.1

Description	Scientific Name	Max Score	Total Score	Query Cover	E value	Per. Ident	Acc. Len	Accession
Paralichthys albigutta voucher FDA 103 cytochrome oxidase subunit 1 (COI) gene, partial cds; mitochondrial	Paralichthys albigutta	725	725	99%	0.0	86.86%	655	KF461215.1
Paralichthys dentatus voucher USNM:FISH:423933 cytochrome oxidase subunit 1 (COI) gene, partial cds; mitochondrial	Paralichthys dentatus	723	723	99%	0.0	86.75%	655	MT455927.1
Paralichthys dentatus voucher USNM:FISH:423811 cytochrome oxidase subunit 1 (COI) gene, partial cds; mitochondrial	Paralichthys dentatus	723	723	99%	0.0	86.84%	648	MT455323.1
Paralichthys albigutta voucher MXV0035 cytochrome oxidase subunit 1 (COI) gene, partial cds; mitochondrial	Paralichthys albigutta	723	723	99%	0.0	86.75%	652	MG837970.1
Actinopterygii environmental sample voucher DE1109067_03 cytochrome oxidase subunit 1 (COI) gene, partial cds; mitochondrial	Actinopterygii environmental sample	723	723	99%	0.0	86.75%	652	KP111488.1
Actinopterygii environmental sample voucher DL0706_054_03 cytochrome oxidase subunit 1 (COI) gene, partial cds; mitochondrial	Actinopterygii environmental sample	721	721	99%	0.0	86.82%	647	KP110873.1
Paralichthys albigutta voucher USNM:FISH:447358 cytochrome oxidase subunit 1 (COI) gene, partial cds; mitochondrial	Paralichthys albigutta	719	719	99%	0.0	86.71%	655	MT455829.1
Paralichthys albigutta voucher USNM:FISH:454751 mitochondrion, complete genome	Paralichthys albigutta	719	719	99%	0.0	86.71%	17035	NC_083031.1
Paralichthys orbignyanus isolate FARG322-07 cytochrome oxidase subunit I (COI) gene, partial cds; mitochondrial	Paralichthys orbignyanus	719	719	99%	0.0	86.71%	652	EU074519.1
Actinopterygii environmental sample voucher DE0210_011_05 cytochrome oxidase subunit 1 (COI) gene, partial cds; mitochondrial	Actinopterygii environmental sample	715	715	98%	0.0	86.76%	644	KP111710.1
Paralichthys albigutta voucher USNM:FISH:451250 cytochrome oxidase subunit 1 (COI) gene, partial cds; mitochondrial	Paralichthys albigutta	713	713	99%	0.0	86.55%	655	MT455012.1

Description	Scientific Name	Max Score	Total Score	Query Cover	E value	Per. Ident	Acc. Len	Accession
Actinopterygii environmental sample voucher DE0210_011_02 cytochrome oxidase subunit 1 (COI) gene, partial cds; mitochondrial	Actinopterygii environmental sample	712	712	98%	0.0	86.72%	642	KP111827.1
Actinopterygii environmental sample voucher DE0210_011_06 cytochrome oxidase subunit 1 (COI) gene, partial cds; mitochondrial	Actinopterygii environmental sample	712	712	98%	0.0	86.72%	642	KP111283.1
Actinopterygii environmental sample voucher DE0210_002_02 cytochrome oxidase subunit 1 (COI) gene, partial cds; mitochondrial	Actinopterygii environmental sample	712	712	98%	0.0	86.72%	642	KP111187.1
Actinopterygii environmental sample voucher DE0210_011_03 cytochrome oxidase subunit 1 (COI) gene, partial cds; mitochondrial	Actinopterygii environmental sample	710	710	97%	0.0	86.89%	635	KP111956.1
Actinopterygii environmental sample voucher DE0210_011_01 cytochrome oxidase subunit 1 (COI) gene, partial cds; mitochondrial	Actinopterygii environmental sample	710	710	98%	0.0	86.70%	641	KP111944.1
Actinopterygii environmental sample voucher DE0911_037_01 cytochrome oxidase subunit 1 (COI) gene, partial cds; mitochondrial	Actinopterygii environmental sample	710	710	97%	0.0	86.89%	635	KP111360.1
Actinopterygii environmental sample voucher DL0711_005_07 cytochrome oxidase subunit 1 (COI) gene, partial cds; mitochondrial	Actinopterygii environmental sample	710	710	97%	0.0	86.89%	635	KP111091.1
Actinopterygii environmental sample voucher DE0210_011_07 cytochrome oxidase subunit 1 (COI) gene, partial cds; mitochondrial	Actinopterygii environmental sample	710	710	98%	0.0	86.60%	644	KP110799.1
Paralichthys dentatus voucher 06-099 cytochrome oxidase subunit 1 (COI) gene, partial cds; mitochondrial	Paralichthys dentatus	710	710	99%	0.0	86.24%	652	KC015754.1
Actinopterygii environmental sample voucher DL0711_005_05 cytochrome oxidase subunit 1 (COI) gene, partial cds; mitochondrial	Actinopterygii environmental sample	706	706	96%	0.0	87.06%	629	KP111700.1

Description	Scientific Name	Max Score	Total Score	Query Cover	E value	Per. Ident	Acc. Len	Accession
Actinopterygii environmental sample voucher AL0410_053_01 cytochrome oxidase subunit 1 (COI) gene, partial cds; mitochondrial	Actinopterygii environmental sample	706	706	96%	0.0	87.06%	633	KP111235.1
Paralichthyidae sp. BOLD:AAO1988 voucher SMSA7168 cytochrome oxidase subunit 1 (COI) gene, partial cds; mitochondrial	Paralichthyidae sp. BOLD:AAO1988	706	706	98%	0.0	86.66%	639	JQ842632.1
Actinopterygii environmental sample voucher DL0711_034_01 cytochrome oxidase subunit 1 (COI) gene, partial cds; mitochondrial	Actinopterygii environmental sample	704	704	97%	0.0	86.73%	635	KP111750.1
Actinopterygii environmental sample voucher DL0711_005_06 cytochrome oxidase subunit 1 (COI) gene, partial cds; mitochondrial	Actinopterygii environmental sample	704	704	97%	0.0	86.73%	635	KP111041.1
Actinopterygii environmental sample voucher DL0711_056_08 cytochrome oxidase subunit 1 (COI) gene, partial cds; mitochondrial	Actinopterygii environmental sample	704	704	97%	0.0	86.73%	635	KP110830.1
Actinopterygii environmental sample voucher AL0410_028_04 cytochrome oxidase subunit 1 (COI) gene, partial cds; mitochondrial	Actinopterygii environmental sample	702	702	97%	0.0	86.71%	634	KP111841.1
Actinopterygii environmental sample voucher AL0410_028_09 cytochrome oxidase subunit 1 (COI) gene, partial cds; mitochondrial	Actinopterygii environmental sample	702	702	97%	0.0	86.71%	634	KP110854.1
Actinopterygii environmental sample voucher DL0711_056_01 cytochrome oxidase subunit 1 (COI) gene, partial cds; mitochondrial	Actinopterygii environmental sample	701	701	96%	0.0	86.90%	631	KP111741.1
Actinopterygii environmental sample voucher DL0711_056_03 cytochrome oxidase subunit 1 (COI) gene, partial cds; mitochondrial	Actinopterygii environmental sample	701	701	96%	0.0	86.90%	627	KP111179.1
Actinopterygii environmental sample voucher DL0711_056_07 cytochrome oxidase subunit 1 (COI) gene,	Actinopterygii environmental sample	701	701	96%	0.0	86.90%	630	KP111103.1

Description	Scientific Name	Max Score	Total Score	Query Cover	E value	Per. Ident	Acc. Len	Accession
partial cds; mitochondrial								
Actinopterygii environmental sample voucher AL0410_028_03 cytochrome oxidase subunit 1 (COI) gene, partial cds; mitochondrial	Actinopterygii environmental sample	699	699	97%	0.0	86.55%	634	KP112075.1
Actinopterygii environmental sample voucher DL0711_056_06 cytochrome oxidase subunit 1 (COI) gene, partial cds; mitochondrial	Actinopterygii environmental sample	699	699	97%	0.0	86.57%	635	KP111884.1
Actinopterygii environmental sample voucher DL0711_005_02 cytochrome oxidase subunit 1 (COI) gene, partial cds; mitochondrial	Actinopterygii environmental sample	697	697	96%	0.0	86.74%	627	KP111731.1
Actinopterygii environmental sample voucher DE0210_011_04 cytochrome oxidase subunit 1 (COI) gene, partial cds; mitochondrial	Actinopterygii environmental sample	697	697	96%	0.0	86.74%	633	KP111115.1
Paralichthys woolmani isolate LPZSF105 cytochrome c oxidase subunit I (COX1) gene, partial cds; mitochondrial	Paralichthys woolmani	693	693	93%	0.0	87.29%	606	MT311625.1
Xystreurys rasile isolate FARG358-07 cytochrome oxidase subunit I (COI) gene, partial cds; mitochondrial	Xystreurys rasile	691	691	99%	0.0	85.94%	652	EU074623.1
Paralichthys dentatus cytochrome oxidase subunit I (COI) gene, partial cds; mitochondrial	Paralichthys dentatus	686	686	96%	0.0	86.31%	629	KX164002.1
Paralichthys californicus voucher JUPA-822 cytochrome oxidase subunit 1 (COI) gene, partial cds; mitochondrial	Paralichthys californicus	686	686	93%	0.0	87.11%	605	KM077536.1
Paralichthys isosceles voucher HRCB:46871 cytochrome oxidase subunit 1 (COI) gene, partial cds; mitochondrial	Paralichthys isosceles	686	686	99%	0.0	85.78%	652	JQ365476.1
Xystreurys rasile isolate FARG218-06 cytochrome oxidase subunit I (COI) gene, partial cds; mitochondrial	Xystreurys rasile	686	686	99%	0.0	85.78%	652	EU074629.1
Xystreurys rasile isolate FARG220-06 cytochrome oxidase subunit I (COI) gene,	Xystreurys rasile	686	686	99%	0.0	85.78%	652	EU074627.1

Description	Scientific Name	Max Score	Total Score	Query Cover	E value	Per. Ident	Acc. Len	Accession
partial cds; mitochondrial								
Xystreurys rasile isolate FARG217-06 cytochrome oxidase subunit I (COI) gene, partial cds; mitochondrial	Xystreurys rasile	686	686	99%	0.0	85.78%	652	EU074624.1
Paralichthys californicus voucher JUPA-823 cytochrome oxidase subunit 1 (COI) gene, partial cds; mitochondrial	Paralichthys californicus	682	682	92%	0.0	87.06%	603	KM019390.1
Paralichthys californicus voucher JUPA-827 cytochrome oxidase subunit 1 (COI) gene, partial cds; mitochondrial	Paralichthys californicus	682	682	92%	0.0	87.17%	602	KM019385.1
Litopenaeus vannamei voucher DAAPV F14 cytochrome oxidase subunit I (COI) gene, partial cds; mitochondrial	Penaeus vannamei	680	680	99%	0.0	85.63%	652	GU324180.1
Xystreurys rasile isolate FARG219-06 cytochrome oxidase subunit I (COI) gene, partial cds; mitochondrial	Xystreurys rasile	680	680	99%	0.0	85.63%	652	EU074626.1
Xystreurys rasile isolate FARG216-06 cytochrome oxidase subunit I (COI) gene, partial cds; mitochondrial	Xystreurys rasile	680	680	99%	0.0	85.63%	652	EU074625.1
Xystreurys rasile isolate FARG359-07 cytochrome oxidase subunit I (COI) gene, partial cds; mitochondrial	Xystreurys rasile	680	680	99%	0.0	85.63%	652	EU074622.1
Verasper moseri voucher UW 118097 cytochrome c oxidase subunit 1 (COI) gene, complete cds; mitochondrial	Verasper moseri	678	678	99%	0.0	85.60%	1557	MH032541.1
Verasper moseri voucher UW 118096 cytochrome c oxidase subunit 1 (COI) gene, complete cds; mitochondrial	Verasper moseri	678	678	99%	0.0	85.60%	1557	MH032540.1
Verasper moseri mitochondrion, complete genome	Verasper moseri	678	678	99%	0.0	85.60%	17443	LC583747.1
Verasper moseri mitochondrion, complete genome	Verasper moseri	678	678	99%	0.0	85.60%	17588	NC_008461.1

Graphic Summary

Distribution of the top 100 Blast Hits on 100 subject sequences

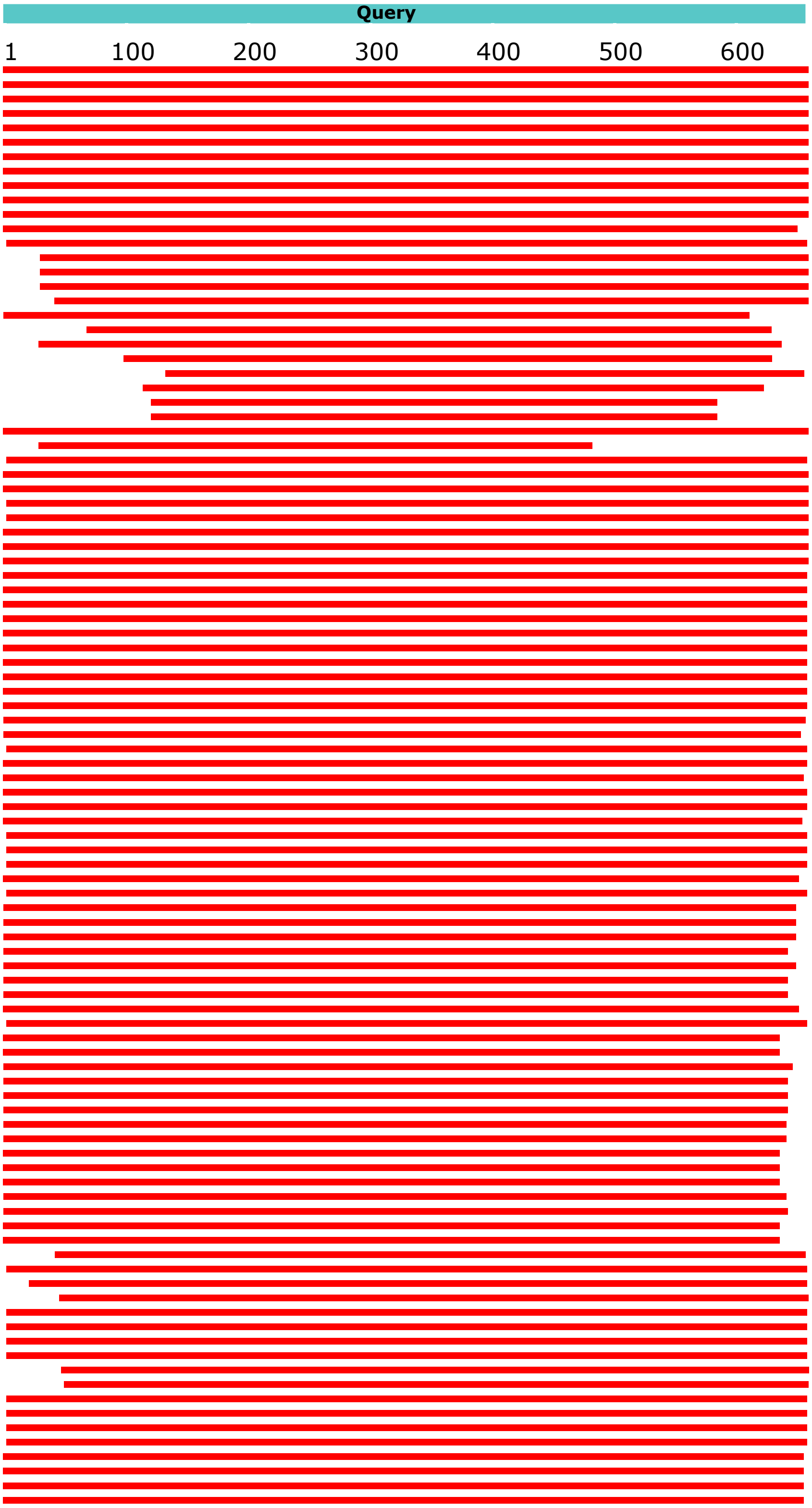

Alignments

Alignment view [Pairwise] ☐ CDS feature [Restore defaults]

Paralichthys olivaceus x Verasper variegatus mitochondrion, complete genome

Sequence ID: **NC_082846.1** Length: 16946 Number of Matches: 1

Range 1: 5552 to 6201

Score	Expect	Identities	Gaps	Strand	Frame
1201 bits(650)	0.0()	650/650(100%)	0/650(0%)	Plus/Plus	

```
Query  1     TCTATCTCGTATTTGGTGCCTGAGCCGGAATAGTGGGGACAGCCCTAAGCCTCCTCATTC  60
             ||||||||||||||||||||||||||||||||||||||||||||||||||||||||||||
Sbjct  5552  TCTATCTCGTATTTGGTGCCTGAGCCGGAATAGTGGGGACAGCCCTAAGCCTCCTCATTC  5611

Query  61    GGGCAGAACTCAGCCAACCTGGTGCTCTCCTAGGGGACGACCAGATTTATAACGTAATCG  120
             ............................................................
```

```
                 ||||||||||||||||||||||||||||||||||||||||||||||||||||||||||||
Sbjct  5612  GGGCAGAACTCAGCCAACCTGGTGCTCTCCTAGGGGACGACCAGATTTATAACGTAATCG  5671

Query  121   TTACCGCACACGCCTTTGTAATAATCTTTTTCATAGTTATACCAATTATGATTGGAGGCT  180
             ||||||||||||||||||||||||||||||||||||||||||||||||||||||||||||
Sbjct  5672  TTACCGCACACGCCTTTGTAATAATCTTTTTCATAGTTATACCAATTATGATTGGAGGCT  5731

Query  181   TTGGCAACTGACTTATCCCCCTGATAATCGGTGCCCCAGACATAGCATTCCCTCGAATAA  240
             ||||||||||||||||||||||||||||||||||||||||||||||||||||||||||||
Sbjct  5732  TTGGCAACTGACTTATCCCCCTGATAATCGGTGCCCCAGACATAGCATTCCCTCGAATAA  5791

Query  241   ATAACATAAGCTTCTGACTTCTACCCCCTTCATTCCTTCTTCTCCTGGCTTCTTCAGGTG  300
             ||||||||||||||||||||||||||||||||||||||||||||||||||||||||||||
Sbjct  5792  ATAACATAAGCTTCTGACTTCTACCCCCTTCATTCCTTCTTCTCCTGGCTTCTTCAGGTG  5851

Query  301   TCGAAGCTGGTGCCGGTACCGGGTGGACTGTCTACCCTCCCCTAGCTAGCAACCTCGCCC  360
             ||||||||||||||||||||||||||||||||||||||||||||||||||||||||||||
Sbjct  5852  TCGAAGCTGGTGCCGGTACCGGGTGGACTGTCTACCCTCCCCTAGCTAGCAACCTCGCCC  5911

Query  361   ATGCTGGAGCCTCAGTAGATCTAACCATCTTTTCACTGCACCTTGCAGGTATTTCATCAA  420
             ||||||||||||||||||||||||||||||||||||||||||||||||||||||||||||
Sbjct  5912  ATGCTGGAGCCTCAGTAGATCTAACCATCTTTTCACTGCACCTTGCAGGTATTTCATCAA  5971

Query  421   TTCTGGGAGCTATCAACTTCATTACTACCATTATTAACATGAAACCCACAACTGTCACAA  480
             ||||||||||||||||||||||||||||||||||||||||||||||||||||||||||||
Sbjct  5972  TTCTGGGAGCTATCAACTTCATTACTACCATTATTAACATGAAACCCACAACTGTCACAA  6031

Query  481   TATACCAAATTCCCCTGTTTGTCTGAGCCGTCCTAATTACGGCTGTCCTGCTGCTCCTCT  540
             ||||||||||||||||||||||||||||||||||||||||||||||||||||||||||||
Sbjct  6032  TATACCAAATTCCCCTGTTTGTCTGAGCCGTCCTAATTACGGCTGTCCTGCTGCTCCTCT  6091

Query  541   CGCTGCCAGTTTTAGCCGCCGGTATTACAATACTGCTTACAGACCGAAACCTTAATACAA  600
             ||||||||||||||||||||||||||||||||||||||||||||||||||||||||||||
Sbjct  6092  CGCTGCCAGTTTTAGCCGCCGGTATTACAATACTGCTTACAGACCGAAACCTTAATACAA  6151

Query  601   CATTCTTTGACCCTGCAGGAGGAGGGGATCCAATCCTCTACCAACACCTG  650
             ||||||||||||||||||||||||||||||||||||||||||||||||||
Sbjct  6152  CATTCTTTGACCCTGCAGGAGGAGGGGATCCAATCCTCTACCAACACCTG  6201
```

Paralichthys olivaceus voucher XM073 cytochrome oxidase subunit I (COI) gene, partial cds; mitochondrial

Sequence ID: **KX254476.1** Length: 655 Number of Matches: 1

Range 1: 6 to 655

Score	Expect	Identities	Gaps	Strand	Frame
1201 bits(650)	0.0()	650/650(100%)	0/650(0%)	Plus/Plus	

```
Query  1    TCTATCTCGTATTTGGTGCCTGAGCCGGAATAGTGGGGACAGCCCTAAGCCTCCTCATTC  60
            ||||||||||||||||||||||||||||||||||||||||||||||||||||||||||||
Sbjct  6    TCTATCTCGTATTTGGTGCCTGAGCCGGAATAGTGGGGACAGCCCTAAGCCTCCTCATTC  65

Query  61   GGGCAGAACTCAGCCAACCTGGTGCTCTCCTAGGGGACGACCAGATTTATAACGTAATCG  120
            ||||||||||||||||||||||||||||||||||||||||||||||||||||||||||||
Sbjct  66   GGGCAGAACTCAGCCAACCTGGTGCTCTCCTAGGGGACGACCAGATTTATAACGTAATCG  125

Query  121  TTACCGCACACGCCTTTGTAATAATCTTTTTCATAGTTATACCAATTATGATTGGAGGCT  180
            ||||||||||||||||||||||||||||||||||||||||||||||||||||||||||||
Sbjct  126  TTACCGCACACGCCTTTGTAATAATCTTTTTCATAGTTATACCAATTATGATTGGAGGCT  185

Query  181  TTGGCAACTGACTTATCCCCCTGATAATCGGTGCCCCAGACATAGCATTCCCTCGAATAA  240
            ||||||||||||||||||||||||||||||||||||||||||||||||||||||||||||
Sbjct  186  TTGGCAACTGACTTATCCCCCTGATAATCGGTGCCCCAGACATAGCATTCCCTCGAATAA  245

Query  241  ATAACATAAGCTTCTGACTTCTACCCCCTTCATTCCTTCTTCTCCTGGCTTCTTCAGGTG  300
            ||||||||||||||||||||||||||||||||||||||||||||||||||||||||||||
Sbjct  246  ATAACATAAGCTTCTGACTTCTACCCCCTTCATTCCTTCTTCTCCTGGCTTCTTCAGGTG  305

Query  301  TCGAAGCTGGTGCCGGTACCGGGTGGACTGTCTACCCTCCCCTAGCTAGCAACCTCGCCC  360
            ||||||||||||||||||||||||||||||||||||||||||||||||||||||||||||
Sbjct  306  TCGAAGCTGGTGCCGGTACCGGGTGGACTGTCTACCCTCCCCTAGCTAGCAACCTCGCCC  365

Query  361  ATGCTGGAGCCTCAGTAGATCTAACCATCTTTTCACTGCACCTTGCAGGTATTTCATCAA  420
            ||||||||||||||||||||||||||||||||||||||||||||||||||||||||||||
Sbjct  366  ATGCTGGAGCCTCAGTAGATCTAACCATCTTTTCACTGCACCTTGCAGGTATTTCATCAA  425

Query  421  TTCTGGGAGCTATCAACTTCATTACTACCATTATTAACATGAAACCCACAACTGTCACAA  480
            ||||||||||||||||||||||||||||||||||||||||||||||||||||||||||||
Sbjct  426  TTCTGGGAGCTATCAACTTCATTACTACCATTATTAACATGAAACCCACAACTGTCACAA  485

Query  481  TATACCAAATTCCCCTGTTTGTCTGAGCCGTCCTAATTACGGCTGTCCTGCTGCTCCTCT  540
            ||||||||||||||||||||||||||||||||||||||||||||||||||||||||||||
Sbjct  486  TATACCAAATTCCCCTGTTTGTCTGAGCCGTCCTAATTACGGCTGTCCTGCTGCTCCTCT  545

Query  541  CGCTGCCAGTTTTAGCCGCCGGTATTACAATACTGCTTACAGACCGAAACCTTAATACAA  600
            ||||||||||||||||||||||||||||||||||||||||||||||||||||||||||||
Sbjct  546  CGCTGCCAGTTTTAGCCGCCGGTATTACAATACTGCTTACAGACCGAAACCTTAATACAA  605

Query  601  CATTCTTTGACCCTGCAGGAGGAGGGGATCCAATCCTCTACCAACACCTG  650
            ||||||||||||||||||||||||||||||||||||||||||||||||||
Sbjct  606  CATTCTTTGACCCTGCAGGAGGAGGGGATCCAATCCTCTACCAACACCTG  655
```

Paralichthys olivaceus voucher HME1 cytochrome oxidase subunit 1 (COI) gene, partial cds; mitochondrial

Sequence ID: **JF952803.1** Length: 652 Number of Matches: 1

Range 1: 3 to 652

Score	Expect	Identities	Gaps	Strand	Frame
1201 bits(650)	0.0()	650/650(100%)	0/650(0%)	Plus/Plus	

```
Query  1    TCTATCTCGTATTTGGTGCCTGAGCCGGAATAGTGGGGACAGCCCTAAGCCTCCTCATTC  60
            ||||||||||||||||||||||||||||||||||||||||||||||||||||||||||||
Sbjct  3    TCTATCTCGTATTTGGTGCCTGAGCCGGAATAGTGGGGACAGCCCTAAGCCTCCTCATTC  62

Query  61   GGGCAGAACTCAGCCAACCTGGTGCTCTCCTAGGGGACGACCAGATTTATAACGTAATCG  120
            ||||||||||||||||||||||||||||||||||||||||||||||||||||||||||||
Sbjct  63   GGGCAGAACTCAGCCAACCTGGTGCTCTCCTAGGGGACGACCAGATTTATAACGTAATCG  122

Query  121  TTACCGCACACGCCTTTGTAATAATCTTTTTCATAGTTATACCAATTATGATTGGAGGCT  180
            ||||||||||||||||||||||||||||||||||||||||||||||||||||||||||||
Sbjct  123  TTACCGCACACGCCTTTGTAATAATCTTTTTCATAGTTATACCAATTATGATTGGAGGCT  182

Query  181  TTGGCAACTGACTTATCCCCCTGATAATCGGTGCCCCAGACATAGCATTCCCTCGAATAA  240
            ||||||||||||||||||||||||||||||||||||||||||||||||||||||||||||
Sbjct  183  TTGGCAACTGACTTATCCCCCTGATAATCGGTGCCCCAGACATAGCATTCCCTCGAATAA  242

Query  241  ATAACATAAGCTTCTGACTTCTACCCCCTTCATTCCTTCTTCTCCTGGCTTCTTCAGGTG  300
            ||||||||||||||||||||||||||||||||||||||||||||||||||||||||||||
Sbjct  243  ATAACATAAGCTTCTGACTTCTACCCCCTTCATTCCTTCTTCTCCTGGCTTCTTCAGGTG  302

Query  301  TCGAAGCTGGTGCCGGTACCGGGTGGACTGTCTACCCTCCCCTAGCTAGCAACCTCGCCC  360
            ||||||||||||||||||||||||||||||||||||||||||||||||||||||||||||
Sbjct  303  TCGAAGCTGGTGCCGGTACCGGGTGGACTGTCTACCCTCCCCTAGCTAGCAACCTCGCCC  362

Query  361  ATGCTGGAGCCTCAGTAGATCTAACCATCTTTTCACTGCACCTTGCAGGTATTTCATCAA  420
            ||||||||||||||||||||||||||||||||||||||||||||||||||||||||||||
Sbjct  363  ATGCTGGAGCCTCAGTAGATCTAACCATCTTTTCACTGCACCTTGCAGGTATTTCATCAA  422

Query  421  TTCTGGGAGCTATCAACTTCATTACTACCATTATTAACATGAAACCCACAACTGTCACAA  480
            ||||||||||||||||||||||||||||||||||||||||||||||||||||||||||||
Sbjct  423  TTCTGGGAGCTATCAACTTCATTACTACCATTATTAACATGAAACCCACAACTGTCACAA  482

Query  481  TATACCAAATTCCCCTGTTTGTCTGAGCCGTCCTAATTACGGCTGTCCTGCTGCTCCTCT  540
            ||||||||||||||||||||||||||||||||||||||||||||||||||||||||||||
Sbjct  483  TATACCAAATTCCCCTGTTTGTCTGAGCCGTCCTAATTACGGCTGTCCTGCTGCTCCTCT  542

Query  541  CGCTGCCAGTTTTAGCCGCCGGTATTACAATACTGCTTACAGACCGAAACCTTAATACAA  600
            ||||||||||||||||||||||||||||||||||||||||||||||||||||||||||||
Sbjct  543  CGCTGCCAGTTTTAGCCGCCGGTATTACAATACTGCTTACAGACCGAAACCTTAATACAA  602

Query  601  CATTCTTTGACCCTGCAGGAGGAGGGGATCCAATCCTCTACCAACACCTG  650
            ||||||||||||||||||||||||||||||||||||||||||||||||||
Sbjct  603  CATTCTTTGACCCTGCAGGAGGAGGGGATCCAATCCTCTACCAACACCTG  652
```

Paralichthys olivaceus isolate CO1B7 cytochrome oxidase subunit I-like (COI) gene, partial sequence; mitochondrial
Sequence ID: **EU266369.1** Length: 713 Number of Matches: 1
Range 1: 31 to 680

Score	Expect	Identities	Gaps	Strand	Frame
1201 bits(650)	0.0()	650/650(100%)	0/650(0%)	Plus/Plus	

```
Query  1    TCTATCTCGTATTTGGTGCCTGAGCCGGAATAGTGGGGACAGCCCTAAGCCTCCTCATTC  60
            ||||||||||||||||||||||||||||||||||||||||||||||||||||||||||||
Sbjct  31   TCTATCTCGTATTTGGTGCCTGAGCCGGAATAGTGGGGACAGCCCTAAGCCTCCTCATTC  90

Query  61   GGGCAGAACTCAGCCAACCTGGTGCTCTCCTAGGGGACGACCAGATTTATAACGTAATCG  120
            ||||||||||||||||||||||||||||||||||||||||||||||||||||||||||||
Sbjct  91   GGGCAGAACTCAGCCAACCTGGTGCTCTCCTAGGGGACGACCAGATTTATAACGTAATCG  150

Query  121  TTACCGCACACGCCTTTGTAATAATCTTTTTCATAGTTATACCAATTATGATTGGAGGCT  180
            ||||||||||||||||||||||||||||||||||||||||||||||||||||||||||||
Sbjct  151  TTACCGCACACGCCTTTGTAATAATCTTTTTCATAGTTATACCAATTATGATTGGAGGCT  210

Query  181  TTGGCAACTGACTTATCCCCCTGATAATCGGTGCCCCAGACATAGCATTCCCTCGAATAA  240
            ||||||||||||||||||||||||||||||||||||||||||||||||||||||||||||
Sbjct  211  TTGGCAACTGACTTATCCCCCTGATAATCGGTGCCCCAGACATAGCATTCCCTCGAATAA  270

Query  241  ATAACATAAGCTTCTGACTTCTACCCCCTTCATTCCTTCTTCTCCTGGCTTCTTCAGGTG  300
            ||||||||||||||||||||||||||||||||||||||||||||||||||||||||||||
Sbjct  271  ATAACATAAGCTTCTGACTTCTACCCCCTTCATTCCTTCTTCTCCTGGCTTCTTCAGGTG  330

Query  301  TCGAAGCTGGTGCCGGTACCGGGTGGACTGTCTACCCTCCCCTAGCTAGCAACCTCGCCC  360
            ||||||||||||||||||||||||||||||||||||||||||||||||||||||||||||
Sbjct  331  TCGAAGCTGGTGCCGGTACCGGGTGGACTGTCTACCCTCCCCTAGCTAGCAACCTCGCCC  390

Query  361  ATGCTGGAGCCTCAGTAGATCTAACCATCTTTTCACTGCACCTTGCAGGTATTTCATCAA  420
            ||||||||||||||||||||||||||||||||||||||||||||||||||||||||||||
Sbjct  391  ATGCTGGAGCCTCAGTAGATCTAACCATCTTTTCACTGCACCTTGCAGGTATTTCATCAA  450

Query  421  TTCTGGGAGCTATCAACTTCATTACTACCATTATTAACATGAAACCCACAACTGTCACAA  480
            ||||||||||||||||||||||||||||||||||||||||||||||||||||||||||||
Sbjct  451  TTCTGGGAGCTATCAACTTCATTACTACCATTATTAACATGAAACCCACAACTGTCACAA  510

Query  481  TATACCAAATTCCCCTGTTTGTCTGAGCCGTCCTAATTACGGCTGTCCTGCTGCTCCTCT  540
            ||||||||||||||||||||||||||||||||||||||||||||||||||||||||||||
Sbjct  511  TATACCAAATTCCCCTGTTTGTCTGAGCCGTCCTAATTACGGCTGTCCTGCTGCTCCTCT  570

Query  541  CGCTGCCAGTTTTAGCCGCCGGTATTACAATACTGCTTACAGACCGAAACCTTAATACAA  600
            ||||||||||||||||||||||||||||||||||||||||||||||||||||||||||||
Sbjct  571  CGCTGCCAGTTTTAGCCGCCGGTATTACAATACTGCTTACAGACCGAAACCTTAATACAA  630

Query  601  CATTCTTTGACCCTGCAGGAGGAGGGGATCCAATCCTCTACCAACACCTG  650
```

```
               ||||||||||||||||||||||||||||||||||||||||||||||||||
Sbjct  631  CATTCTTTGACCCTGCAGGAGGAGGGGATCCAATCCTCTACCAACACCTG  680
```

Paralichthys olivaceus voucher HUM I-00297 cytochrome c oxidase subunit 1 (COI) gene, complete cds; mitochondrial
Sequence ID: **MH032483.1** Length: 1551 Number of Matches: 1
Range 1: 53 to 702

Score	Expect	Identities	Gaps	Strand	Frame
1195 bits(647)	0.0()	649/650(99%)	0/650(0%)	Plus/Plus	

```
Query  1    TCTATCTCGTATTTGGTGCCTGAGCCGGAATAGTGGGGACAGCCCTAAGCCTCCTCATTC  60
            ||||||||||||||||||||||||||||||||||||||||||||||||||||||||||||
Sbjct  53   TCTATCTCGTATTTGGTGCCTGAGCCGGAATAGTGGGGACAGCCCTAAGCCTCCTCATTC  112

Query  61   GGGCAGAACTCAGCCAACCTGGTGCTCTCCTAGGGGACGACCAGATTTATAACGTAATCG  120
            ||||||||||||||||||||||||||||||||||||||||||||||||||||||||||||
Sbjct  113  GGGCAGAACTCAGCCAACCTGGTGCTCTCCTAGGGGACGACCAGATTTATAACGTAATCG  172

Query  121  TTACCGCACACGCCTTTGTAATAATCTTTTTCATAGTTATACCAATTATGATTGGAGGCT  180
            ||||||||||||||||||||||||||||||||||||||||||||||||||||||||||||
Sbjct  173  TTACCGCACACGCCTTTGTAATAATCTTTTTCATAGTTATACCAATTATGATTGGAGGCT  232

Query  181  TTGGCAACTGACTTATCCCCCTGATAATCGGTGCCCCAGACATAGCATTCCCTCGAATAA  240
            ||||||||||||||||||||||||||||||||||||||||||||||||||||||||||||
Sbjct  233  TTGGCAACTGACTTATCCCCCTGATAATCGGTGCCCCAGACATAGCATTCCCTCGAATAA  292

Query  241  ATAACATAAGCTTCTGACTTCTACCCCCTTCATTCCTTCTTCTCCTGGCTTCTTCAGGTG  300
            ||||||||||||||||||||||||||||||||||||||||||||||||||||||||||||
Sbjct  293  ATAACATAAGCTTCTGACTTCTACCCCCTTCATTCCTTCTTCTCCTGGCTTCTTCAGGTG  352

Query  301  TCGAAGCTGGTGCCGGTACCGGGTGGACTGTCTACCCTCCCCTAGCTAGCAACCTCGCCC  360
            ||||||||||||||||||||||||||||||||||||||||||||||||||||||||||||
Sbjct  353  TCGAAGCTGGTGCCGGTACCGGGTGGACTGTCTACCCTCCCCTAGCTAGCAACCTCGCCC  412

Query  361  ATGCTGGAGCCTCAGTAGATCTAACCATCTTTTCACTGCACCTTGCAGGTATTTCATCAA  420
            ||||||||||||||||||||||||||||||||||||||||||||||||||||||||||||
Sbjct  413  ATGCTGGAGCCTCAGTAGATCTAACCATCTTTTCACTGCACCTTGCAGGTATTTCATCAA  472

Query  421  TTCTGGGAGCTATCAACTTCATTACTACCATTATTAACATGAAACCCACAACTGTCACAA  480
            ||||||||||||||||||| ||||||||||||||||||||||||||||||||||||||||
Sbjct  473  TTCTGGGAGCTATCAACTTTATTACTACCATTATTAACATGAAACCCACAACTGTCACAA  532

Query  481  TATACCAAATTCCCCTGTTTGTCTGAGCCGTCCTAATTACGGCTGTCCTGCTGCTCCTCT  540
            ||||||||||||||||||||||||||||||||||||||||||||||||||||||||||||
Sbjct  533  TATACCAAATTCCCCTGTTTGTCTGAGCCGTCCTAATTACGGCTGTCCTGCTGCTCCTCT  592

Query  541  CGCTGCCAGTTTTAGCCGCCGGTATTACAATACTGCTTACAGACCGAAACCTTAATACAA  600
            ||||||||||||||||||||||||||||||||||||||||||||||||||||||||||||
Sbjct  593  CGCTGCCAGTTTTAGCCGCCGGTATTACAATACTGCTTACAGACCGAAACCTTAATACAA  652

Query  601  CATTCTTTGACCCTGCAGGAGGAGGGGATCCAATCCTCTACCAACACCTG  650
            ||||||||||||||||||||||||||||||||||||||||||||||||||
Sbjct  653  CATTCTTTGACCCTGCAGGAGGAGGGGATCCAATCCTCTACCAACACCTG  702
```

Taxonomy

Reports

- ### Lineage

Organism	Blast Name	Score	Number of Hits	Description
Bilateria	animals		126	
.Actinopterygii	bony fishes		125	
..Pleuronectoidei	bony fishes		92	
...Paralichthys olivaceus x Verasper variegatus	bony fishes	1201	2	**Paralichthys olivaceus x Verasper variegatus hits**
...Paralichthys olivaceus	bony fishes	1201	31	**Paralichthys olivaceus hits**
...Paralichthys aestuarius	bony fishes	819	1	**Paralichthys aestuarius hits**
...Paralichthys squamilentus	bony fishes	808	1	**Paralichthys squamilentus hits**
...Paralichthys patagonicus	bony fishes	808	4	**Paralichthys patagonicus hits**
...Paralichthys adspersus	bony fishes	802	2	**Paralichthys adspersus hits**
...Paralichthys californicus	bony fishes	774	7	**Paralichthys californicus hits**
...Paralichthys dentatus	bony fishes	734	18	**Paralichthys dentatus hits**
...Paralichthys woolmani	bony fishes	728	2	**Paralichthys woolmani hits**
...Paralichthys albigutta	bony fishes	725	6	**Paralichthys albigutta hits**
...Paralichthys orbignyanus	bony fishes	719	2	**Paralichthys orbignyanus hits**
...Paralichthyidae sp. BOLD:AAO1988	bony fishes	706	1	**Paralichthyidae sp. BOLD:AAO1988 hits**

...Xystreurys rasile	bony fishes	691	8	Xystreurys rasile hits
...Paralichthys isosceles	bony fishes	686	2	Paralichthys isosceles hits
...Verasper moseri	bony fishes	678	5	Verasper moseri hits
..Actinopterygii environmental sample	bony fishes	734	33	Actinopterygii environmental sample hits
.Penaeus vannamei	crustaceans	680	1	Penaeus vannamei hits

- **Organism**

Description	Score	E value	Accession
Paralichthys olivaceus x Verasper variegatus [bony fishes]			
Paralichthys olivaceus x Verasper variegatus mitochondrion, complete genome	1201	0.0	NC_082846
Paralichthys olivaceus x Verasper variegatus mitochondrion, complete genome	1201	0.0	OR353704
Paralichthys olivaceus (Japanese flounder) [bony fishes]			
Paralichthys olivaceus voucher XM073 cytochrome oxidase subunit I (COI) gene, partial cds; mitochondrial	1201	0.0	KX254476
Paralichthys olivaceus voucher HME1 cytochrome oxidase subunit 1 (COI) gene, partial cds; mitochondrial	1201	0.0	JF952803
Paralichthys olivaceus isolate CO1B7 cytochrome oxidase subunit I-like (COI) gene, partial sequence; mitochondrial	1201	0.0	EU266369
Paralichthys olivaceus voucher HUM I-00297 cytochrome c oxidase subunit 1 (COI) gene, complete cds; mitochondrial	1195	0.0	MH032483
Paralichthys olivaceus voucher XM072 cytochrome oxidase subunit I (COI) gene, partial cds; mitochondrial	1195	0.0	KX254475
Paralichthys olivaceus voucher TWS 029 cytochrome oxidase subunit I (COI) gene, partial cds; mitochondrial	1195	0.0	MG220576
Paralichthys olivaceus voucher HUM I-00296 cytochrome c oxidase subunit 1 (COI) gene, complete cds; mitochondrial	1190	0.0	MH032482
Paralichthys olivaceus voucher XM074 cytochrome oxidase subunit I (COI) gene, partial cds; mitochondrial	1190	0.0	KX254477
Paralichthys olivaceus voucher HME2 cytochrome oxidase subunit 1 (COI) gene, partial cds; mitochondrial	1190	0.0	JF952804
Paralichthys olivaceus isolate CO1B8 cytochrome oxidase subunit I (COI) gene, partial cds; mitochondrial	1190	0.0	EU266368
Paralichthys olivaceus mitochondrion, complete genome	1190	0.0	NC_002386
Paralichthys olivaceus mitochondrial DNA, complete genome	1190	0.0	AB028664
Paralichthys olivaceus isolate WJC173 cytochrome oxidase subunit 1 (COI) gene, partial cds; mitochondrial	1173	0.0	MK617161
Paralichthys olivaceus strain West.5.3 cytochrome c oxidase subunit I (COI) gene, partial cds; mitochondrial	1173	0.0	MK560569
Paralichthys olivaceus voucher IOCASFY-RCB09-Po5 cytochrome oxidase subunit I (COI) gene, partial cds; mitochondrial	1146	0.0	KU236831
Paralichthys olivaceus voucher IOCASFY-RCB09-Po1 cytochrome oxidase subunit I (COI) gene, partial cds; mitochondrial	1134	0.0	KU236827
Paralichthys olivaceus voucher IOCASFY-RCB09-Po2 cytochrome oxidase subunit I (COI) gene, partial cds; mitochondrial	1134	0.0	KU236828
Paralichthys olivaceus voucher IOCASFY-RCB09-Po3 cytochrome oxidase subunit I (COI) gene, partial cds; mitochondrial	1134	0.0	KU236829
Paralichthys olivaceus voucher IOCASFY-RCB09-Po4 cytochrome oxidase subunit I (COI) gene, partial cds; mitochondrial	1134	0.0	KU236830
Paralichthys olivaceus voucher IOCASFY-RCB09-Po6 cytochrome oxidase subunit I (COI) gene, partial cds; mitochondrial	1134	0.0	KU236832
Paralichthys olivaceus voucher IOCASFY-RCB09-Po7 cytochrome oxidase subunit I (COI) gene, partial cds; mitochondrial	1129	0.0	KU236833
Paralichthys olivaceus isolate j4 cytochrome c oxidase subunit I (COX1) gene, partial cds; mitochondrial	1109	0.0	MZ317453
Paralichthys olivaceus isolate PKU_10872 cytochrome oxidase subunit I (COI) gene, partial cds; mitochondrial	1101	0.0	KP835312
Paralichthys olivaceus isolate ASIZP0914829 cytochrome oxidase subunit I (COI) gene, partial cds; mitochondrial	1003	0.0	KU945107
Paralichthys olivaceus isolate F00223 cytochrome oxidase subunit I (COI) gene, partial cds;	1002	0.0	JQ738445

Description	Score	E value	Accession
mitochondrial			
Paralichthys olivaceus isolate sample_77 cytochrome c oxidase subunit I (COI) gene, partial cds; mitochondrial	965	0.0	MW027188
Paralichthys olivaceus isolate PKU 4489 cytochrome oxidase subunit I (COI) gene, partial cds; mitochondrial	946	0.0	KR052269
Paralichthys olivaceus mitochondrial COX1 gene for cytochrome c oxidase subunit 1, partial cds, isolate: 005_55	915	0.0	LC126342
Paralichthys olivaceus isolate PKU 4490 cytochrome oxidase subunit I gene, partial cds; mitochondrial	843	0.0	KF965424
Paralichthys olivaceus isolate PKU 4489 cytochrome oxidase subunit I gene, partial cds; mitochondrial	837	0.0	KF965438
Paralichthys olivaceus isolate F00222 cytochrome oxidase subunit I (COI) gene, partial cds; mitochondrial	813	0.0	JQ738444
Paralichthys aestuarius (Cortez flounder) [bony fishes]			
Paralichthys aestuarius voucher SIO 07-157 cytochrome c oxidase subunit 1 (COI) gene, complete cds; mitochondrial	819	0.0	MH032480
Paralichthys squamilentus (broad flounder) [bony fishes]			
Paralichthys squamilentus voucher KUT 5205 cytochrome oxidase subunit 1 (COI) gene, partial cds; mitochondrial	808	0.0	KF930230
Paralichthys patagonicus (Patagonian flounder) [bony fishes]			
Paralichthys patagonicus voucher HRCB:53035 cytochrome oxidase subunit 1 (COI) gene, partial cds; mitochondrial	808	0.0	JQ365477
Paralichthys patagonicus voucher DAAPV F28 cytochrome oxidase subunit I (COI) gene, partial cds; mitochondrial	798	0.0	GU324191
Paralichthys patagonicus voucher HRCB:53034 cytochrome oxidase subunit 1 (COI) gene, partial cds; mitochondrial	787	0.0	JQ365478
Paralichthys patagonicus voucher LBPV53033 cytochrome oxidase subunit 1 (COI) gene, partial cds; mitochondrial	787	0.0	JX124847
Paralichthys adspersus (fine flounder) [bony fishes]			
Paralichthys adspersus mitochondrion, complete genome	802	0.0	NC_057273
Paralichthys adspersus mitochondrion, complete genome	802	0.0	MW288827
Paralichthys californicus (California flounder) [bony fishes]			
Paralichthys californicus voucher SIO 03-51 cytochrome c oxidase subunit 1 (COI) gene, complete cds; mitochondrial	774	0.0	MH032481
Paralichthys californicus mitochondrion, complete genome	763	0.0	MT859134
Paralichthys californicus voucher MFC144 cytochrome oxidase subunit 1 (COI) gene, partial cds; mitochondrial	763	0.0	GU440447
Paralichthys californicus voucher KUT 456 cytochrome oxidase subunit 1 (COI) gene, partial cds; mitochondrial	763	0.0	KF930225
Paralichthys californicus voucher JUPA-822 cytochrome oxidase subunit 1 (COI) gene, partial cds; mitochondrial	686	0.0	KM077536
Paralichthys californicus voucher JUPA-823 cytochrome oxidase subunit 1 (COI) gene, partial cds; mitochondrial	682	0.0	KM019390
Paralichthys californicus voucher JUPA-827 cytochrome oxidase subunit 1 (COI) gene, partial cds; mitochondrial	682	0.0	KM019385
Paralichthys dentatus (summer flounder) [bony fishes]			
Paralichthys dentatus voucher USNM:FISH:431132 cytochrome oxidase subunit 1 (COI) gene, partial cds; mitochondrial	734	0.0	MT455401
Paralichthys dentatus voucher 07-045 cytochrome oxidase subunit 1 (COI) gene, partial cds; mitochondrial	734	0.0	KC015757
Paralichthys dentatus voucher 09-399 cytochrome oxidase subunit 1 (COI) gene, partial cds; mitochondrial	734	0.0	KC015760
Paralichthys dentatus voucher 07-123 cytochrome oxidase subunit 1 (COI) gene, partial cds; mitochondrial	730	0.0	KC015758
Paralichthys dentatus voucher USNM:FISH:423822 cytochrome oxidase subunit 1 (COI) gene, partial cds; mitochondrial	728	0.0	MT455243
Paralichthys dentatus voucher USNM:FISH:423835 cytochrome oxidase subunit 1 (COI) gene, partial cds; mitochondrial	728	0.0	MT455651

Description	Score	E value	Accession
Paralichthys dentatus voucher USNM:FISH:423812 cytochrome oxidase subunit 1 (COI) gene, partial cds; mitochondrial	728	0.0	MT455665
Paralichthys dentatus voucher USNM:FISH:423932 cytochrome oxidase subunit 1 (COI) gene, partial cds; mitochondrial	728	0.0	MT455883
Paralichthys dentatus mitochondrion, complete genome	728	0.0	NC_029476
Paralichthys dentatus mitochondrion, complete genome	728	0.0	KU053334
Paralichthys dentatus voucher 06-117 cytochrome oxidase subunit 1 (COI) gene, partial cds; mitochondrial	728	0.0	KC015755
Paralichthys dentatus voucher 07-092 cytochrome oxidase subunit 1 (COI) gene, partial cds; mitochondrial	728	0.0	KC015759
Paralichthys dentatus voucher 09-400 cytochrome oxidase subunit 1 (COI) gene, partial cds; mitochondrial	728	0.0	KC015756
Paralichthys dentatus voucher USNM:FISH:423832 cytochrome oxidase subunit 1 (COI) gene, partial cds; mitochondrial	725	0.0	MT456241
Paralichthys dentatus voucher USNM:FISH:423933 cytochrome oxidase subunit 1 (COI) gene, partial cds; mitochondrial	723	0.0	MT455927
Paralichthys dentatus voucher USNM:FISH:423811 cytochrome oxidase subunit 1 (COI) gene, partial cds; mitochondrial	723	0.0	MT455323
Paralichthys dentatus voucher 06-099 cytochrome oxidase subunit 1 (COI) gene, partial cds; mitochondrial	710	0.0	KC015754
Paralichthys dentatus cytochrome oxidase subunit I (COI) gene, partial cds; mitochondrial	686	0.0	KX164002
Actinopterygii environmental sample [bony fishes]			
Actinopterygii environmental sample voucher DE1109058_05 cytochrome oxidase subunit 1 (COI) gene, partial cds; mitochondrial	734	0.0	KP110988
Actinopterygii environmental sample voucher DE1109067_02 cytochrome oxidase subunit 1 (COI) gene, partial cds; mitochondrial	734	0.0	KP111602
Actinopterygii environmental sample voucher DE1109057_01 cytochrome oxidase subunit 1 (COI) gene, partial cds; mitochondrial	734	0.0	KP111781
Actinopterygii environmental sample voucher DE1109057_03 cytochrome oxidase subunit 1 (COI) gene, partial cds; mitochondrial	734	0.0	KP112034
Actinopterygii environmental sample voucher DE1109058_01 cytochrome oxidase subunit 1 (COI) gene, partial cds; mitochondrial	728	0.0	KP111879
Actinopterygii environmental sample voucher DE1109058_02 cytochrome oxidase subunit 1 (COI) gene, partial cds; mitochondrial	728	0.0	KP110848
Actinopterygii environmental sample voucher DE1109067_01 cytochrome oxidase subunit 1 (COI) gene, partial cds; mitochondrial	728	0.0	KP111573
Actinopterygii environmental sample voucher DL0706_054_04 cytochrome oxidase subunit 1 (COI) gene, partial cds; mitochondrial	725	0.0	KP111797
Actinopterygii environmental sample voucher DE1109067_03 cytochrome oxidase subunit 1 (COI) gene, partial cds; mitochondrial	723	0.0	KP111488
Actinopterygii environmental sample voucher DL0706_054_03 cytochrome oxidase subunit 1 (COI) gene, partial cds; mitochondrial	721	0.0	KP110873
Actinopterygii environmental sample voucher DE0210_011_05 cytochrome oxidase subunit 1 (COI) gene, partial cds; mitochondrial	715	0.0	KP111710
Actinopterygii environmental sample voucher DE0210_011_02 cytochrome oxidase subunit 1 (COI) gene, partial cds; mitochondrial	712	0.0	KP111827
Actinopterygii environmental sample voucher DE0210_011_06 cytochrome oxidase subunit 1 (COI) gene, partial cds; mitochondrial	712	0.0	KP111283
Actinopterygii environmental sample voucher DE0210_002_02 cytochrome oxidase subunit 1 (COI) gene, partial cds; mitochondrial	712	0.0	KP111187
Actinopterygii environmental sample voucher DE0210_011_03 cytochrome oxidase subunit 1 (COI) gene, partial cds; mitochondrial	710	0.0	KP111956
Actinopterygii environmental sample voucher DE0210_011_01 cytochrome oxidase subunit 1 (COI) gene, partial cds; mitochondrial	710	0.0	KP111944
Actinopterygii environmental sample voucher DE0911_037_01 cytochrome oxidase subunit 1 (COI) gene, partial cds; mitochondrial	710	0.0	KP111360
Actinopterygii environmental sample voucher DL0711_005_07 cytochrome oxidase subunit 1 (COI) gene, partial cds; mitochondrial	710	0.0	KP111091
Actinopterygii environmental sample voucher DE0210_011_07 cytochrome oxidase subunit 1 (COI) gene, partial cds; mitochondrial	710	0.0	KP110799

Description	Score	E value	Accession
Actinopterygii environmental sample voucher DL0711_005_05 cytochrome oxidase subunit 1 (COI) gene, partial cds; mitochondrial	706	0.0	KP111700
Actinopterygii environmental sample voucher AL0410_053_01 cytochrome oxidase subunit 1 (COI) gene, partial cds; mitochondrial	706	0.0	KP111235
Actinopterygii environmental sample voucher DL0711_034_01 cytochrome oxidase subunit 1 (COI) gene, partial cds; mitochondrial	704	0.0	KP111750
Actinopterygii environmental sample voucher DL0711_005_06 cytochrome oxidase subunit 1 (COI) gene, partial cds; mitochondrial	704	0.0	KP111041
Actinopterygii environmental sample voucher DL0711_056_08 cytochrome oxidase subunit 1 (COI) gene, partial cds; mitochondrial	704	0.0	KP110830
Actinopterygii environmental sample voucher AL0410_028_04 cytochrome oxidase subunit 1 (COI) gene, partial cds; mitochondrial	702	0.0	KP111841
Actinopterygii environmental sample voucher AL0410_028_09 cytochrome oxidase subunit 1 (COI) gene, partial cds; mitochondrial	702	0.0	KP110854
Actinopterygii environmental sample voucher DL0711_056_01 cytochrome oxidase subunit 1 (COI) gene, partial cds; mitochondrial	701	0.0	KP111741
Actinopterygii environmental sample voucher DL0711_056_03 cytochrome oxidase subunit 1 (COI) gene, partial cds; mitochondrial	701	0.0	KP111179
Actinopterygii environmental sample voucher DL0711_056_07 cytochrome oxidase subunit 1 (COI) gene, partial cds; mitochondrial	701	0.0	KP111103
Actinopterygii environmental sample voucher AL0410_028_03 cytochrome oxidase subunit 1 (COI) gene, partial cds; mitochondrial	699	0.0	KP112075
Actinopterygii environmental sample voucher DL0711_056_06 cytochrome oxidase subunit 1 (COI) gene, partial cds; mitochondrial	699	0.0	KP111884
Actinopterygii environmental sample voucher DL0711_005_02 cytochrome oxidase subunit 1 (COI) gene, partial cds; mitochondrial	697	0.0	KP111731
Actinopterygii environmental sample voucher DE0210_011_04 cytochrome oxidase subunit 1 (COI) gene, partial cds; mitochondrial	697	0.0	KP111115
Paralichthys woolmani (speckled flounder) [bony fishes]			
Paralichthys woolmani voucher SIO-09-205 cytochrome c oxidase subunit 1 (COI) gene, complete cds; mitochondrial	728	0.0	MH032484
Paralichthys woolmani isolate LPZSF105 cytochrome c oxidase subunit I (COX1) gene, partial cds; mitochondrial	693	0.0	MT311625
Paralichthys albigutta (Gulf flounder) [bony fishes]			
Paralichthys albigutta voucher FDA 103 cytochrome oxidase subunit 1 (COI) gene, partial cds; mitochondrial	725	0.0	KF461215
Paralichthys albigutta voucher MXV0035 cytochrome oxidase subunit 1 (COI) gene, partial cds; mitochondrial	723	0.0	MG837970
Paralichthys albigutta voucher USNM:FISH:447358 cytochrome oxidase subunit 1 (COI) gene, partial cds; mitochondrial	719	0.0	MT455829
Paralichthys albigutta voucher USNM:FISH:454751 mitochondrion, complete genome	719	0.0	NC_083031
Paralichthys albigutta voucher USNM:FISH:454751 mitochondrion, complete genome	719	0.0	OR546183
Paralichthys albigutta voucher USNM:FISH:451250 cytochrome oxidase subunit 1 (COI) gene, partial cds; mitochondrial	713	0.0	MT455012
Paralichthys orbignyanus [bony fishes]			
Paralichthys orbignyanus isolate FARG322-07 cytochrome oxidase subunit I (COI) gene, partial cds; mitochondrial	719	0.0	EU074519
Paralichthys orbignyanus isolate FARG286-07 cytochrome oxidase subunit I (COI) gene, partial cds; mitochondrial	719	0.0	EU074520
Paralichthyidae sp. BOLD:AAO1988 [bony fishes]			
Paralichthyidae sp. BOLD:AAO1988 voucher SMSA7168 cytochrome oxidase subunit 1 (COI) gene, partial cds; mitochondrial	706	0.0	JQ842632
Xystreurys rasile [bony fishes]			
Xystreurys rasile isolate FARG358-07 cytochrome oxidase subunit I (COI) gene, partial cds; mitochondrial	691	0.0	EU074623
Xystreurys rasile isolate FARG218-06 cytochrome oxidase subunit I (COI) gene, partial cds; mitochondrial	686	0.0	EU074629
Xystreurys rasile isolate FARG220-06 cytochrome oxidase subunit I (COI) gene, partial cds; mitochondrial	686	0.0	EU074627

Description	Score	E value	Accession
Xystreurys rasile isolate FARG217-06 cytochrome oxidase subunit I (COI) gene, partial cds; mitochondrial	686	0.0	EU074624
Xystreurys rasile isolate FARG219-06 cytochrome oxidase subunit I (COI) gene, partial cds; mitochondrial	680	0.0	EU074626
Xystreurys rasile isolate FARG221-06 cytochrome oxidase subunit I (COI) gene, partial cds; mitochondrial	680	0.0	EU074628
Xystreurys rasile isolate FARG216-06 cytochrome oxidase subunit I (COI) gene, partial cds; mitochondrial	680	0.0	EU074625
Xystreurys rasile isolate FARG359-07 cytochrome oxidase subunit I (COI) gene, partial cds; mitochondrial	680	0.0	EU074622
Paralichthys isosceles [bony fishes]			
Paralichthys isosceles voucher HRCB:46871 cytochrome oxidase subunit 1 (COI) gene, partial cds; mitochondrial	686	0.0	JQ365476
Paralichthys isosceles voucher HRCB:46870 cytochrome oxidase subunit 1 (COI) gene, partial cds; mitochondrial	686	0.0	JQ365475
Penaeus vannamei (Pacific white shrimp) [crustaceans]			
Litopenaeus vannamei voucher DAAPV F14 cytochrome oxidase subunit I (COI) gene, partial cds; mitochondrial	680	0.0	GU324180
Verasper moseri (barfin flounder) [bony fishes]			
Verasper moseri voucher UW 118097 cytochrome c oxidase subunit 1 (COI) gene, complete cds; mitochondrial	678	0.0	MH032541
Verasper moseri voucher UW 118096 cytochrome c oxidase subunit 1 (COI) gene, complete cds; mitochondrial	678	0.0	MH032540
Verasper moseri mitochondrion, complete genome	678	0.0	LC583747
Verasper moseri mitochondrion, complete genome	678	0.0	NC_008461
Verasper moseri mitochondrion, complete genome	678	0.0	EF025506

- ## Taxonomy

Taxonomy	Number of hits	Number of Organisms	Description
Bilateria	126	17	
. Actinopterygii	125	16	
.. Pleuronectoidei	92	15	
... Paralichthys olivaceus x Verasper variegatus	2	1	Paralichthys olivaceus x Verasper variegatus hits
... Paralichthyidae	85	13	
.... Paralichthys	76	11	
..... Paralichthys olivaceus	31	1	Paralichthys olivaceus hits
..... Paralichthys aestuarius	1	1	Paralichthys aestuarius hits
..... Paralichthys squamilentus	1	1	Paralichthys squamilentus hits
..... Paralichthys patagonicus	4	1	Paralichthys patagonicus hits
..... Paralichthys adspersus	2	1	Paralichthys adspersus hits
..... Paralichthys californicus	7	1	Paralichthys californicus hits
..... Paralichthys dentatus	18	1	Paralichthys dentatus hits
..... Paralichthys woolmani	2	1	Paralichthys woolmani hits
..... Paralichthys albigutta	6	1	Paralichthys albigutta hits
..... Paralichthys orbignyanus	2	1	Paralichthys orbignyanus hits
..... Paralichthys isosceles	2	1	Paralichthys isosceles hits
.... Paralichthyidae sp. BOLD:AAO1988	1	1	Paralichthyidae sp. BOLD:AAO1988 hits
.... Xystreurys rasile	8	1	Xystreurys rasile hits
... Verasper moseri	5	1	Verasper moseri hits
.. Actinopterygii environmental sample	33	1	Actinopterygii environmental sample hits
. Penaeus vannamei	1	1	Penaeus vannamei hits

Top
Follow NCBI

Connect with NLM

National Library of Medicine
8600 Rockville Pike
Bethesda, MD 20894

Web Policies
FOIA
HHS Vulnerability Disclosure

Help
Accessibility
Careers

- NLM
- NIH
- HHS
- USA.gov
